# Metabolic risk factors attributed burden in Iran at national and subnational levels, 1990 to 2019

**DOI:** 10.3389/fpubh.2023.1149719

**Published:** 2023-06-01

**Authors:** Soroush Moradi, Amirhossein Parsaei, Sahar Saeedi Moghaddam, Armin Aryannejad, Sina Azadnajafabad, Negar Rezaei, Baharnaz Mashinchi, Zahra Esfahani, Parnian Shobeiri, Nazila Rezaei, GBD 2019 Iran MRF Collaborators, Soroush Moradi, Mohsen Naghavi, Bagher Larijani, Farshad Farzadfar

**Affiliations:** ^1^Non-Communicable Diseases Research Center, Endocrinology and Metabolism Population Sciences Institute, Tehran University of Medical Sciences, Tehran, Iran; ^2^Experimental Medicine Research Center, Tehran University of Medical Sciences, Tehran, Iran; ^3^Endocrinology and Metabolism Research Center, Endocrinology and Metabolism Clinical Sciences Institute, Tehran University of Medical Sciences, Tehran, Iran; ^4^School of Medicine, Tehran University of Medical Sciences, Tehran, Iran; ^5^Institute for Health Metrics and Evaluation, University of Washington, Seattle, WA, United States; ^6^Department of Health Metrics Sciences, School of Medicine, University of Washington, Seattle, WA, United States

**Keywords:** Global Burden of Disease, cardiometabolic risk factors, hypertension, hyperglycemia, obesity, hyperlipidemia, Iran

## Abstract

**Introduction:**

Metabolic risk factors (MRFs) predispose populations to a variety of chronic diseases with a huge burden globally. With the increasing burden of these risk factors in Iran, in this study, we aimed to report the estimated burden attributed to MRFs at national and subnational scales in Iran, from 1990 to 2019.

**Methods:**

Based on the comparative risk assessment method of the Global Burden of Disease (GBD) Study 2019, data of deaths and disability-adjusted life years (DALYs) attributable to four top MRFs in Iran including high systolic blood pressure (SBP), high fasting plasma glucose (FPG), high body mass index (BMI), and high low-density lipoprotein (LDL) for the 1990–2019 period, were extracted. The socio-demographic index (SDI) was used to report the data based on the corresponding socio-economic stratifications. The results were reported in national and subnational 31 provinces of Iran to discover disparities regarding the attributable burden to MRFs. Furthermore, we reported the causes of diseases to which the attributable burden to MRFs was related.

**Results:**

Overall, the age-standardized high LDL, high SBP, high BMI, and high FPG-attributed death rate changed by −45.1, −35.6, +2.8, and +19.9% from 1990 to 2019, respectively. High SBP was the leading risk factor regarding attributed age-standardized death rates reaching 157.8 (95% uncertainty interval: 135.3–179.1) and DALY rates reaching 2973.4 (2652.2–3280.2) per 100,000 person-years, in 2019. All rates increased with aging, and men had higher rates except for the +70 years age group. At the subnational level, provinces in the middle SDI quintile had the highest death and DALY rates regarding all four MRFs. Total deaths, DALYs, YLLs and YLDs number by the causes of diseases linked to MRFs increased over the study period. Cardiovascular diseases, diabetes mellitus, and kidney diseases were the main causes of burden of disease attributable to MRFs.

**Conclusion:**

Herein, we found divergent patterns regarding the burden of MRFs as well as disparities in different regions, sex, and age groups for each risk factor and related causes. This could provide policymakers with a clearer vision toward more appropriate decision-making and resource allocation to prevent the burden of MRFs in Iran.

## 1. Introduction

Being among the most leading causes of mortality and premature deaths around the world, non-communicable diseases (NCDs) were previously known to be mostly limited to developed and high-income countries (1, 2). However, they have also become a major public health concern in developing regions of the world mainly due to epidemiological transitions in such countries during the past decades (1). Iran is also a developing country experiencing an epidemiological transitional period toward modernization in the contemporary age and is dealing with the increasing burden of various NCDs and the responsible risk factors (3, 4). The most burdensome NCDs nationally in Iran have been ischemic heart disease, stroke, and diabetes mellitus based on the most recent estimations, with diverse epidemiology subnationally and for different socio-demographic stratifications of the Iranian population (5–8). Furthermore, among these top causes of NCDs burden in the country, ischemic heart disease was the prominent cause of burden in all provinces showing the heavy ongoing burden of this cause in the country (6).

The four main metabolic risk factors (MRFs), including high systolic blood pressure (SBP), high fasting plasma glucose (FPG), high body mass index (BMI), and high low-dense lipoproteins (LDL) account for a large proportion of deaths related to a wide range of diseases causing a remarkable burden worldwide and also in Iran (9, 10). Based on the 2019 Global Burden of Diseases (GBD) study, high SBP and high LDL were responsible for the greatest number of cardiovascular disease (CVD) mortalities. The high FPG and high BMI also accounted for a large number of CVD and diabetes mellitus and kidney disease (DM/KD) mortalities (9). Previous reports on cardio-metabolic annual deaths attributed to MRFs in the Middle East region (including Iran) represent more than 400 thousand annual cardio-metabolic and diabetic deaths attributed to high SBP and more than 100 thousand deaths attributed to each of the three other mentioned MRFs (11).

It is worth mentioning that the diverse population of Iran comprises different ethnicities with distinct lifestyles and genetic backgrounds, which jointly might cause disparities in this country (4, 5, 12, 13). Therefore, it would be of great importance to report and evaluate the burden of different diseases and risk factors in this country and compare results among its provinces to elucidate possible differences and inequalities and gain a clearer understanding of possible disparities. Recent publications have provided the evidence on burden of diseases and risk factor trends in Iran and its provinces showing various disparities in this regard (5, 12, 14); however, a closer look at the attributable burden to MRFs is essential to reveal the trends and disparities.

In the current study, we aimed to obtain GBD 2019 estimations on the four abovementioned MRFs to demonstrate and compare their attributable deaths and disability-adjusted life years (DALYs), years of lost life (YLLs), and years lived with disability (YLDs) among 31 provinces of Iran over a 30-year period from 1990 to 2019, for the first time. The results of the present study could provide policymakers and health authorities with a clearer vision guiding them toward more appropriate decision-making to prevent the burden of MRFs and related disorders as well as rational and proper allocation of resources among the population in the future.

## 2. Materials and methods

### 2.1. Data source

This research has been conducted as part of the Global Burden of Diseases, Injuries, and Risk Factors Study (GBD), coordinated by the Institute for Health Metrics and Evaluation and reported the results based on the Guidelines for Accurate and Transparent Health Estimates Reporting (the GATHER statement) (15). Details of the GBD data estimation framework for the burden of diseases, injuries, and risk factors are published and accessible elsewhere (9, 16). The entry data were obtained through the GBD results tool which is publicly available (17) using the top four metabolic risk factors in Iran based on the literature (10), namely, high SBP, high FPG, high BMI, and high LDL cholesterol (18). The data source for the estimation of the attributable burden to these risk factors in Iran was extracted via the global health data exchange system (GHDx) query online tool (17). The findings of this query revealed that the GBD study has obtained the primary data from surveys, scientific pieces of literature, reports, and cross-sectional studies conducted in Iran from 1990 to 2019 (Supplementary Tables 1–4).

### 2.2. Risk factor estimation framework

The 2019 GBD study used a six-step comparative risk assessment (CRA) to estimate the burden of risk factors: 1. risk-outcome pair inclusion, 2. exposure risk estimation, 3. exposure level estimation, 4. determining the counterfactual level of exposure, 5. estimation of theoretical minimum risk exposure level value (TMREL), and 6. calculation of population-attributable fractions (PAFs) and summary exposure values (SEVs) for the attributable burden to each risk factor. The selection of risk-outcome pairs was performed based on published systematic reviews to extract relative risks (RRs) for each risk factor (9). Meta-analysis of RRs from these studies was conducted as a function of exposure (9). Then, for the newly proposed or evaluated risk-outcome pair, a *p*-value of < 0.05 was considered a significant association after taking into account sources of potential bias. Estimation of risk exposure also was reached through a systematic analysis of the previously published studies and surveys (9). PAFs were eventually calculated—the process of PAF calculation is described elsewhere (9)—representing the proportion of a specific risk that would be reduced in a specific year if the exposure to that risk factor in the past was within ideal exposure. The rates of risk-related deaths, DALYs, YLLs, and YLDs were ultimately calculated by multiplying the rates in each age and sex combination and location by the PAF (ranging from 0 to 1) calculated for each risk factor (9).

### 2.3. Variable definition

MRFs evaluated in the present study include high SBP, high FPG, high BMI, and high LDL. The definition of each risk factor by their values has been assigned based on the TMREL as the values higher than these ranges were the definition of that risk factor, as follows: high FPG is defined as FPG higher than 4.8–5.4 mmol/L; high SBP is defined as brachial SBP >110 to 115 mmHg as the TMREL for SBP. For high BMI, in adults (≥20 years old), the cutoff was considered BMI >20 to 25 kg/m^2^, and for younger population (<20 years old), it was defined based on the definition of the International Obesity Task Force standards (19). The TMREL for high LDL among the population was 0.7–1.3 mmol/L (9).

The socio-demographic index (SDI) was used as an instrument to pin each area at the subnational level on the scale of development categorizing provinces into five quintiles, namely, low, low-middle, middle, high-middle, and high SDI. This index is calculated based on income per capita, education (based on the average schooling years for people over 15 years old), and fertility rates (for women under 25 years) (20). Finally, we utilized SDI to compare the subnational burden attributable to each risk factor between different quintiles.

Accordingly, the number of deaths, DALYs, YLLs, and YLDs (all ages) and the age-standardized rates were used to report the attributed burden of the four MRFs to each risk factor annually based on different locations and SDI quintiles, age groups, and sex (male, female, and both), as well as 22 related causes of deaths. DALYs were defined as the number of years lost due to disease, disability, or death, which is estimated as the sum of YLLs and YLDs. Thirty-one provinces of Iran were the geographic scale for subnational data reporting in this study and for investigating disparities by the epidemiologic measures of the burden attributable to the MRFs.

### 2.4. Statistical analysis

Age-standardized rates per 100,000 person-years (PY) were used to report the standardized data based on the national population to make the results comparable among provinces at the subnational scale. The values in this article were reported in point estimation accompanied by the 95% uncertainty intervals (95% UI) extracted using the 25th and 975th ranked draws of the uncertainty distribution by taking 1,000 samples from the posterior distribution (9). Percent change was calculated between 1990 and 2019 and represented only the change between the beginning and ending years of the period. The visualization, presentation, and analysis of data in the current study were performed using R studio software v4.2 and STATA v.13.1 for Windows.

## 3. Results

### 3.1. National and subnational deaths attributable to metabolic risk factors

**Figure 1:**
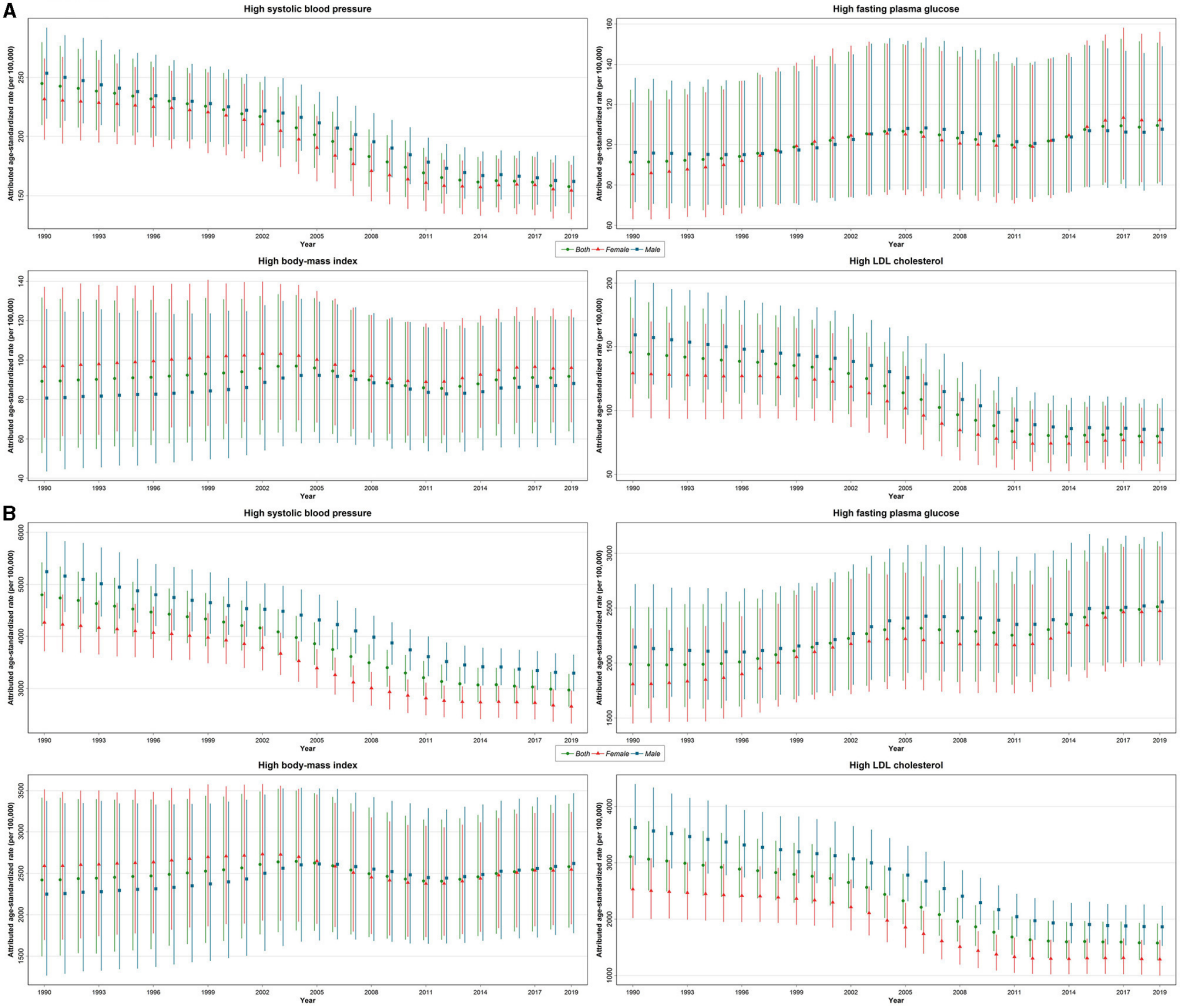
Time trends of age-standardized death **(A)** and DALY **(B)** rates (per 100,000 person-year) attributable to metabolic risk factors including high SBP, high FPG, high BMI, and high LDL by year and sex with 95% uncertainty interval from 1990 to 2019.

At the national level, high SBP-attributed death rates were found to be constantly higher than the other risk factors. However, the high SBP-attributed death rates per 100,000 PY decreased by −35.6% (95% UI: −42.3 to −31.3%) from 244.8 (209.5–279.6) in 1990 to 157.8 (135.3–179.1) per 100,000 PY in 2019. Regarding high FPG, the death rate increased by 19.9% (7.9–35.2%), from 91.4 (68.4–127.3) in 1990 to 109.5 (80.8–150.7) per 100,000 PY in 2019. In the case of high BMI, the death rates increased slightly by 2.8% (−13.1–32.6%) from 89.2 (52.9–131.7) in 1990 to 91.7 (63.9–122.1) per 100,000 PY in 2019. Regarding high LDL, the death rates decreased by −45.1% (−50.7 to −40.3%) from 145.5 (109.3–188.8) in 1990 to 79.9 (58.2–105.1) per 100,000 PY in 2019 (Table 1; Figure 1A). Additional information on the number of deaths is available in Appendix 1.

**Table 1:** Age-standardized death, DALY, YLL, and YLD rates (per 100,000 person-year) attributable to metabolic risk factors including high SBP, high FPG, high BMI, and high LDL and percent changes between 1990 and 2019 for male subjects, female subjects, and both.

**Risk factor**	**Measure**	Attributed age-standardized rate (per 100,000 person-year)^*^						**% Change (1990–2019)** ^*^		
		**1990**			**2019**					
		**Both**	**Female**	**Male**	**Both**	**Female**	**Male**	**Both**	**Female**	**Male**
High systolic blood pressure	Deaths	244.8 (209.5 to 279.6)	231.5 (197.1 to 265.8)	253.4 (214.8 to 291.8)	157.8 (135.3 to 179.1)	154.5 (130.2 to 176)	162.1 (140.5 to 183.4)	−35.6 (−42.3 to −31.3)	−33.3 (−43.2 to −26.6)	−36 (−41.2 to −30.6)
	DALYs	4,800.2 (4,200.5 to 5,423.5)	4,266.4 (3,714.5 to 4,858)	5,247.5 (4,540.8 to 6,009.4)	2,973.4 (2,652.2 to 3,280.2)	2,657.4 (2,331.5 to 2,961.1)	3,295.7 (2,949.8 to 3,650.7)	−38.1 (−43.8 to −33.8)	−37.7 (−45.8 to −32)	−37.2 (−42.4 to −31.8)
	YLLs	4,541.8 (3,955.1 to 5,155.6)	3,993.1 (3,451.4 to 4,555.1)	5,003.6 (4,314 to 5,766.3)	2,710.5 (2,400.8 to 3,010.1)	2,392.7 (2,095.2 to 2,680.7)	3,034.7 (2,707.9 to 3,380.3)	−40.3 (−46.2 to −36.1)	−40.1 (−48.5 to −34.2)	−39.4 (−44.7 to −33.9)
	YLDs	258.4 (185.4 to 336.9)	273.3 (197.3 to 355)	243.9 (175.6 to 320.5)	262.9 (190.1 to 342.3)	264.7 (191 to 342.2)	261.1 (190.1 to 342.3)	1.7 (−1.7 to 5.5)	−3.2 (−7.6 to 2)	7.1 (2.6 to 11.4)
High fasting plasma glucose	Deaths	91.4 (68.4 to 127.3)	85.4 (63 to 120.9)	96.2 (71.4 to 133.2)	109.5 (80.8 to 150.7)	112.2 (81.5 to 156.1)	107.6 (79.8 to 148.9)	19.9 (7.9 to 35.2)	31.3 (14.8 to 52.5)	11.9 (−1.4 to 28.5)
	DALYs	1,988.1 (1,601.4 to 2,516)	1,808.2 (1,450 to 2,314.3)	2,144.1 (1,708.5 to 2,720)	2,511.2 (2,017.4 to 3,108.7)	2,472.5 (1,979.6 to 3,062.7)	2,555.6 (2,028.4 to 3,194.5)	26.3 (14.9 to 39.2)	36.7 (22.5 to 53.4)	19.2 (7.5 to 33.8)
	YLLs	1,595.7 (1,244.9 to 2,107.8)	1,419.3 (1,108.4 to 1,890.8)	1,748.7 (1,345.9 to 2,291)	1,791.5 (1,401.4 to 2,303.1)	1,726.3 (1,337 to 2,224.3)	1,862.4 (1,447.5 to 2,429.4)	12.3 (0.6 to 25.6)	21.6 (6.2 to 40.6)	6.5 (−6.5 to 22.9)
	YLDs	392.4 (276.4 to 528.6)	388.9 (273.5 to 530)	395.4 (277.7 to 530.3)	719.7 (504.3 to 966.4)	746.1 (523.1 to 998)	693.2 (484.3 to 928.1)	83.4 (76.1 to 91.5)	91.8 (83.7 to 100.7)	75.3 (67.5 to 84.2)
High body–mass index	Deaths	89.2 (52.9 to 131.7)	96.7 (60.6 to 137.2)	80.7 (43.5 to 125.8)	91.7 (63.9 to 122.1)	96.1 (68.5 to 125.6)	88.1 (57.9 to 121.5)	2.8 (−13.1 to 32.6)	−0.7 (−16.6 to 28.3)	9.1 (−10.8 to 57.2)
	DALYs	2,419.6 (1,497.6 to 3,414.7)	2,589.6 (1,695.4 to 3,516.8)	2,248.7 (1,265.1 to 3,376.4)	2,580.9 (1,845.1 to 3,337.3)	2,545.9 (1,886.3 to 3,238.4)	2,617.7 (1,778.2 to 3,468.9)	6.7 (−9.5 to 35.6)	−1.7 (−15.3 to 22.5)	16.4 (−4.9 to 68.8)
	YLLs	2,026.8 (1,245.4 to 2,881.7)	2,115 (1,381.9 to 2,897.2)	1,932.3 (1,082.7 to 2,909.5)	1,864.2 (1,337.1 to 2,413.9)	1,755.7 (1,304.5 to 2,232.3)	1,974.1 (1,353.9 to 2,637.9)	−8 (−21.9 to 17)	−17 (−29.1 to 4)	2.2 (−17.1 to 48.1)
	YLDs	392.7 (225.8 to 598.4)	474.6 (284.9 to 701.5)	316.4 (168.6 to 509.9)	716.8 (462.2 to 1,021.5)	790.1 (523 to 1,113.5)	643.6 (404.7 to 931.8)	82.5 (59 to 125.9)	66.5 (47.6 to 99.2)	103.4 (68.9 to 188.4)
High LDL cholesterol	Deaths	145.5 (109.3 to 188.8)	129.2 (94.5 to 172.3)	159.2 (120.9 to 202.5)	79.9 (58.2 to 105.1)	75.1 (52.4 to 101.7)	85.2 (63.9 to 109.5)	−45.1 (−50.7 to −40.3)	−41.9 (−48.6 to −34)	−46.5 (−53 to −41.2)
	DALYs	3,106.6 (2,528.4 to 3,789.1)	2,528.9 (2,017.7 to 3,120.2)	3,620.9 (2,960.9 to 4,398.9)	1,574.5 (1,268.6 to 1,922.4)	1,287.6 (996.7 to 1,626.7)	1,862.3 (1,523.2 to 2,233.1)	−49.3 (−54.7 to −45.1)	−49.1 (−55.1 to −43.1)	−48.6 (−54.3 to −43.5)
	YLLs	3,012.3 (2,443.7 to 3,665.1)	2,428.7 (1,936.9 to 3,000.6)	3,532.2 (2,897.4 to 4,293.3)	1,489.5 (1,194.6 to 1,823.9)	1,202.6 (929.7 to 1,514.5)	1,777.3 (1,453.1 to 2,130)	−50.6 (−56 to −46.1)	−50.5 (−56.7 to −44.3)	−49.7 (−55.4 to −44.5)
	YLDs	94.3 (62.9 to 135.2)	100.2 (66.4 to 146.8)	88.7 (59.2 to 125.4)	85 (56.4 to 121.5)	85 (56.3 to 124.3)	85.1 (56.7 to 120.4)	−9.9 (−12.6 to −7.4)	−15.2 (−18 to −12.3)	−4.1 (−7.7 to −1.1)

* Data in parentheses are 95% Uncertainty Intervals (95% UIs).

**Figure 2:**
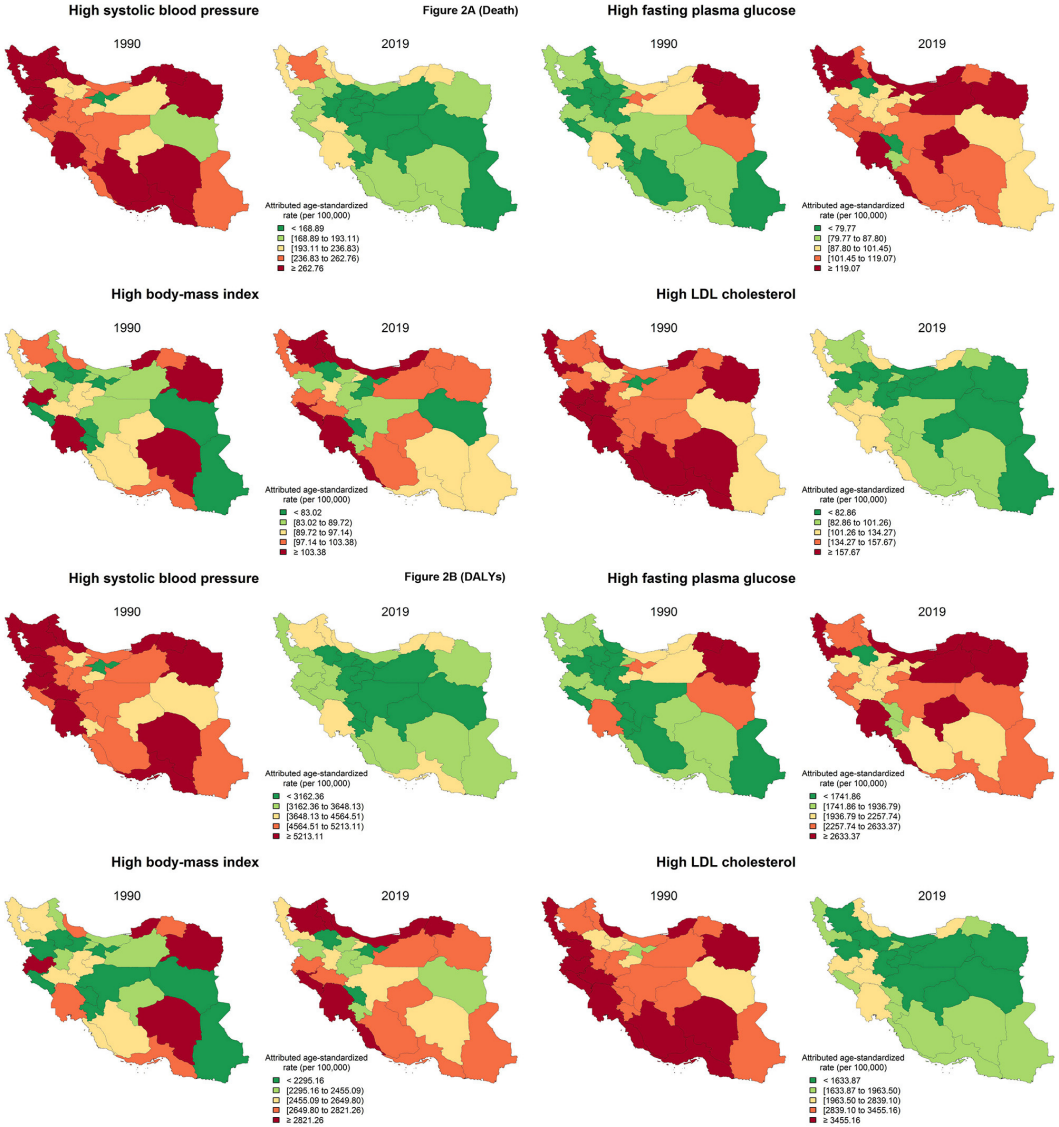
Age-standardized death **(A)** and DALY **(B)** rates (per 100,000 person-year) attributable to metabolic risk factors including high SBP, high FPG, high BMI, and high LDL in 31 provinces of Iran in 1990 and 2019 for both sexes.

At the subnational level (Figure 2A), considering high SBP age-standardized death rates in 1990 and 2019, central provinces had relatively lower rates compared to the others. In 1990, the lowest to highest death rates attributable to high SBP among 31 provinces of Iran were from 129.6 (Tehran) to 310.9 per 100,000 PY (Khorasan-e-Razavi), while in 2019, the lowest to highest death rate was from 68.9 (Tehran) to 237.1 per 100,000 PY (East Azerbaijan). Regarding high FPG, provinces located in the west and southeast had lower death rates. In 1990, the lowest to highest high FPG attributed to age-standardized death rates among provinces reported from 58.9 (Zanjan) to 140.8 per 100,000 PY (Khorasan-e-Razavi), while in 2019, it was from 76.2 (Zanjan) to 164.3 per 100,000 PY (Khuzestan). In terms of high BMI, from 1990 to 2019, central provinces of Iran had relatively lower death rates than other provinces. In 1990, the lowest to highest death rate attributable to high BMI was from 64.1 (Chaharmahal and Bakhtiari) to 110.3 per 100,000 PY (Golestan), and in 2019, it was from 63.6 (Tehran) to 126.5 per 100,000 PY (Golestan). Considering high LDL, in 1990, provinces located in the south and southwest were in a worse condition than the others, while central and eastern provinces had relatively lower death rates. In 2019, the condition of southern provinces improved, but southwestern provinces still had the highest death rates. The lowest to highest death rates attributable to high LDL in 1990 were from 81.0 (Tehran) to 176.4 per 100,000 PY (Gilan), while in 2019, death rates ranged from 42.8 (Tehran) to 115.9 per 100,000 PY (Golestan) (Supplementary Figure 1A; Supplementary Tables 5–8).

### 3.2. National and subnational DALYs, YLLs, and YLDs attributable to metabolic risk factors

Among the four MRFs, high SBP was steadily the top leading risk factor regarding attributed age-standardized DALY rates from 1990 to 2019. For both sexes, the DALY rate decreased by −38.1% (−43.8 to −33.8%) from 4,800.2 (4,200.5–5,423.5, YLLs/DALYs = 94.6%) in 1990 to 2,973.4 (2,652.2–3,280.2, YLLs/DALYs = 91.1%) per 100,000 PY in 2019. For both sexes, YLL rates followed the same pattern, decreasing over this time period, while YLD rates slightly increased only in male subjects and decreased in female subjects (Table 1; Figure 1B; Supplementary Figure 2). Additional information on numbers is available in Appendix 1.

High FPG-attributed DALY rates increased by 26.3% (14.9–39.2%) from 1,988.1 (1,601.4–2,516.0, YLLs/DALYs = 80.2%) in 1990 to 2,511.2 (2,017.4–3108.7, YLLs/DALYs = 71.3%) per 100,000 PY in 2019 (Figure 1B). With reference to high BMI, the attributed DALY rate was 2,419.6 (1,497.6–3,414.7, YLLs/DALYs = 83.7%) in 1990, which increased by 6.7% (−9.5–35.6%) to 2,580.9 (1,845.1–3,337.3, YLLs/DALYs = 72.2%) per 100,000 PY in 2019. Concerning the high LDL-attributed burden, DALY rates decreased by −49.3% (−54.7 to −45.1%) from 3,106.6 (2,528.4–3,789.1, YLLs/DALYs = 96.9%) in 1990 to 1,574.5 (1,268.6–1,922.4, YLLs/DALYs = 94.5%) per 100,000 PY in 2019 (Table 1; Figure 1B; Supplementary Figure 2).

At the subnational level (Figure 2B), similar to death rates, the high SBP-attributed DALY rates from 1990 to 2019 in central provinces remained relatively lower than in other provinces. In 1990, the lowest to highest DALY rates attributable to high SBP ranged from 2,401.7 (Tehran) to 6,179.4 per 100,000 PY (Hormozgan), while it ranged from 1,323.0 (Tehran) to 4,518.3 per 100,000 PY (Golestan) in 2019. Regarding high FPG, the attributed DALY rates were lower in western and southeastern provinces in 1990 and 2019. In 1990, the lowest to highest high FPG-attributed DALY rates ranged from 1,330.5 (Zanjan) to 2,881.6 per 100,000 PY (Khorasan-e-Razavi) compared to 1,698.5 (Zanjan) to 3,759.4 per 100,000 PY (Khuzestan) in 2019. Considering high BMI, central provinces had a better status over this period. The lowest to highest DALY rates attributable to high BMI ranged from 1,722.5 (Chaharmahal and Bakhtiari) to 3,061.7 per 100,000 PY (Golestan) and ranged from 1,887.5 (Chaharmahal and Bakhtiari) to 3,281.9 per 100,000 PY (Khuzestan) in 2019. In terms of high LDL-attributed DALY rates, central provinces remained at a better status than the others, and in 2019, western provinces reached the top of the list in the case of higher rates. In 1990, the lowest to highest DALYs ranged from 1,644.7 (Tehran) to 4,146.7 per 100,000 PY (Golestan) compared to 825.1 (Tehran) to 2,488.8 per 100,000 PY (Golestan) in 2019 (Supplementary Figures 1B, 3). Additional information on YLLs and YLDs of provinces are shown in Supplementary Tables 5–8.

### 3.3. Metabolic risk factors burden based on SDI regions

Considering high SBP, between 1990 and 2019, the death rates of all SDI quintiles decreased. In 2019, the highest death rates belonged to low-middle and middle SDI quintiles (~115/100,000 PY) and the lowest was for high-SDI and low-SDI quintiles (~200/100,000 PY). DALYs attributable to high SBP also followed a similar pattern. Regarding high FPG, the death rates increased in all SDI quintiles, and the rates in middle, high-middle, and high-SDI quintiles (~115/100,000 PY) were slightly higher than low-middle and low-SDI quintiles (~105/100,000 PY). With respect to high BMI, the death rates in 2019 were slightly higher than in 1990 in all SDI quintiles. By 2019, the middle SDI quintile had the highest death rates (~100/100,000 PY) compared to the lowest death rate reported for the low-SDI quintile (~90/100,000 PY). Concerning high LDL, between 1990 and 2019, the death rates in all SDI quintiles decreased, and the middle SDI quintile had the highest death rates (~100/100,000 PY).

Similar to death rates, for all four MRFs, the DALYs burden in provinces with the middle SDI quintile was the highest. Considering high SBP, between 1990 and 2019, the burden of DALYs decreased for all SDI quintiles. In 2019, the highest DALY rates were in the middle SDI (~3,500/100,000 PY) and the lowest in low-SDI and high-SDI quintiles (~2,700/100,000 PY). Considering high FPG, in 2019, the highest DALY rates were reported in the middle and high-SDI quintiles (~2,500/100,000 PY), and the least DALY rates were reported in the low-middle SDI quintile (~2,300/100,000 PY). Regarding high BMI, by 2019, the DALY rate of different quintiles showed less disparity than in 1990. In the year 2019, the most DALY rates were in the middle SDI quintile (~2,700/100,000 PY), while the least was in the low-SDI quintile (~2,500/100,000 PY). Regarding high LDL, between 1990 and 2019, DALYs decreased in all SDI quintiles. The highest was in the middle SDI (~2,000/100,000 PY) and the lowest was in the high SDI (~1,500/100,000 PY).

### 3.4. Age-related burden attributable to metabolic risk factors

**Figure 3:**
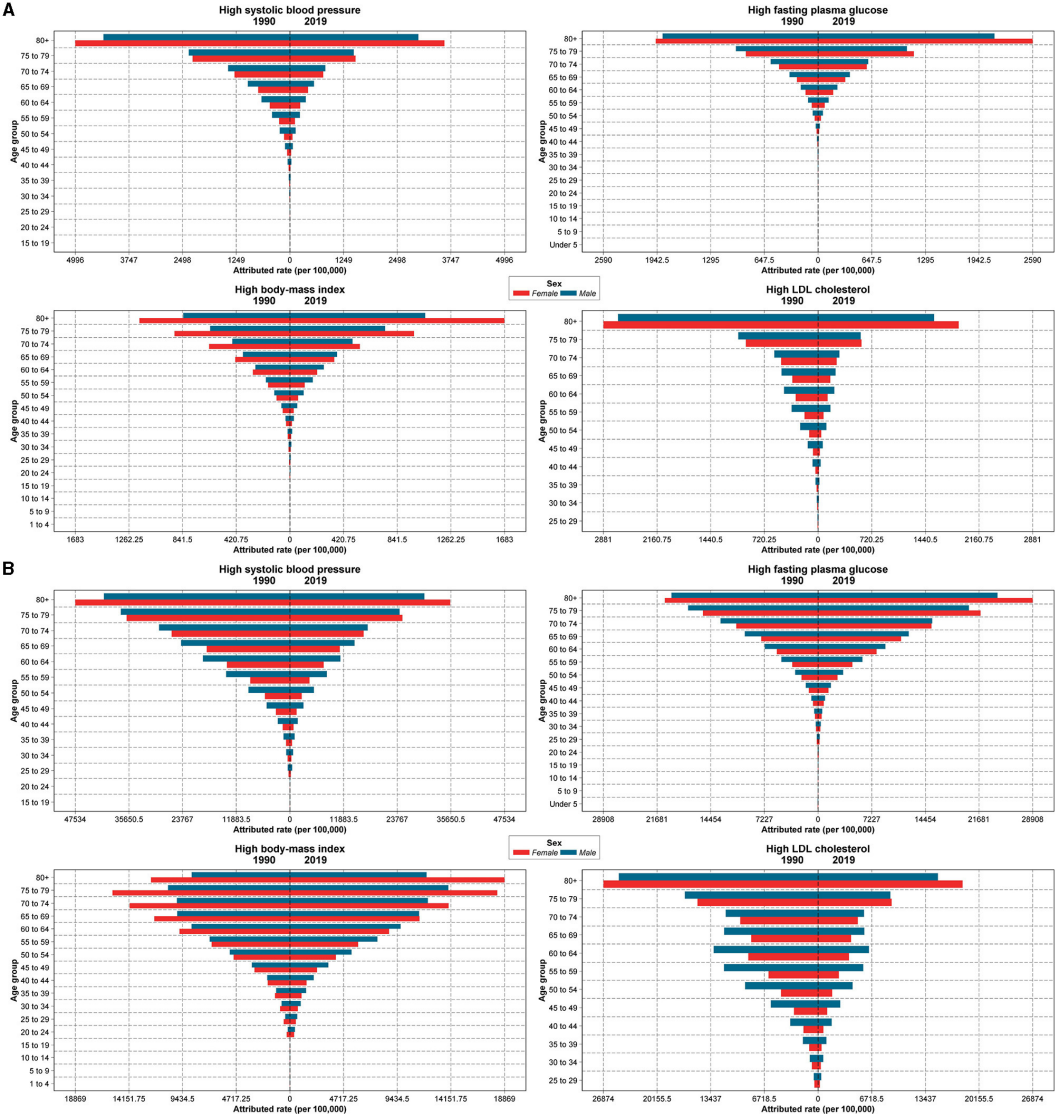
Death **(A)** and DALY **(B)** rates (per 100,000 person-year) attributable to metabolic risk factors including high SBP, high FPG, high BMI, and high LDL based on age groups and sex (red, females; blue, males) in 1990 and 2019.

In terms of age-related deaths, the rates attributable to all four MRFs increased with age, and the rates were always higher in male subjects in the under 75-year-old population and reversed after this age, except for high BMI, where the rates for men and women changed at the age of 70 years and over (Figure 3). The high SBP and high LDL-attributed death rates decreased in almost all age groups from 1990 to 2019. In terms of DALYs, all MRFs had a similar trend of death rates increasing by age in both sexes. Compared to 1990, YLL rates attributable to high SBP were less in all age groups in 2019 and increased by age, while YLD rates were higher in 2019 than in 1990 in all age groups. Moreover, YLD rates were more in female subjects than male subjects in almost all age groups. Considering high FPG, YLL rates increased in both sexes by 2019 and increased by age. YLD rates in 2019 were also noticeably higher in all age groups than in 1990, higher in female subjects and increased by age. In terms of high BMI, from 1990 to 2019, attributed YLL rates increased with age in both 1990 and 2019. Compared to men, YLL rates were higher in women >60 years in 1990, and among women >70 years in 2019. YLD rates increased over time in all age groups, and female subjects had higher YLD rates than male subjects in all age groups. High LDL-attributed YLL rates decreased in both sexes by 2019, and in the +80 age groups, YLL rates were higher in women than men. YLD rates in both 2019 and 1990 were higher in older age groups, while, generally, YLD rates decreased slightly in each age group compared to 1990 (Supplementary Figure 4).

### 3.5. Metabolic risk factors burden by causes

**Figure 4:**
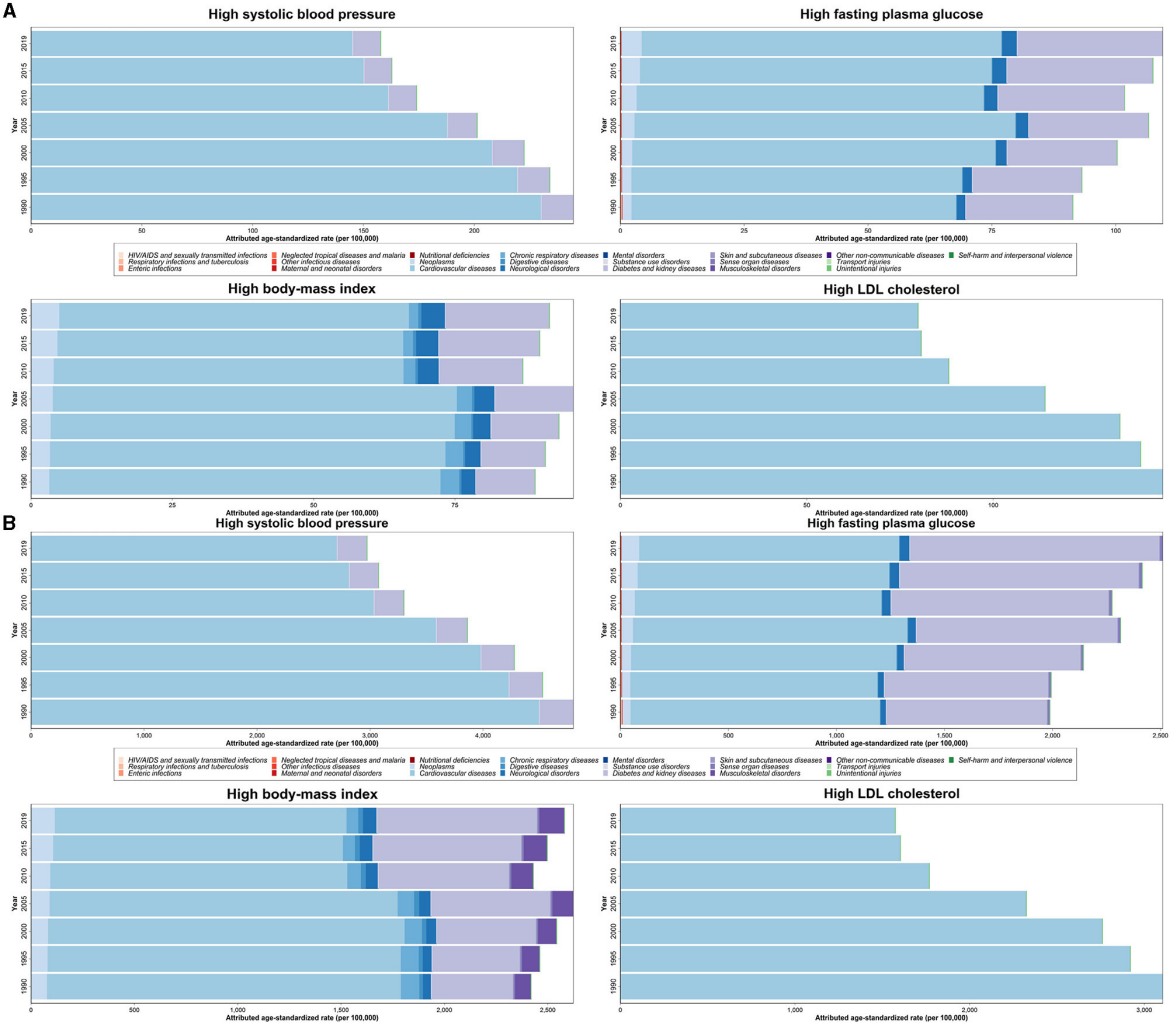
Age-standardized death **(A)** and DALY **(B)** rates (per 100,000 person-year) attributable to metabolic risk factors including high SBP, high FPG, high BMI, and high LDL by 22 main causes of deaths in 1990 through 2019 for both sexes.

From 1990 to 2019, the total number of deaths, DALYs, YLLs, and YLDs caused by MRFs increased over time. The highest attributed rate of deaths, DALYs, and YLLs in all four risk factors was due to cardiovascular diseases (CVDs). Diabetes and kidney diseases (DM/KDs) were the second most prevalent cause of deaths, DALYs, and YLLs rate attributable to all risk factors, except for high LDL, in which attributed deaths, DALYs, YLLs, and YLDs rate were almost only associated with CVDs. Considering YLDs attributable to high BMI, DM/KDs and musculoskeletal disorders were the most common causes. However, in high FPG-attributed YLDs, DM/KDs and CVDs were the most common causes. High SBP-attributed YLDs were caused mostly by CVDs followed by DM/KDs (Figure 4; Supplementary Figure 5).

## 4. Discussion

The results of the present study revealed that generally, the high SBP-attributed death and DALY rates were higher than three other MRFs in the entire period from 1990 to 2019. The age-standardized high LDL, high SBP, high BMI, and high FPG-attributed death rates changed by −45.1, −35.6, +2.8, and +19.9% from 1990 to 2019, respectively. Age-related death and DALY rates showed increasing trends in aging in both sexes. In addition, male subjects had higher rates of death and DALY attributable to MRFs than female subjects most of the time, especially among <70 years age groups. Provinces in the middle SDI quintile had highest burden of deaths and DALYs; however, we did not find any discernible pattern between MRFs attributed burden and SDI levels. A subnational analysis demonstrated disparities among provinces of Iran regarding deaths and DALYs attributed to MRFs, mostly for high FPG and high BMI but less difference was found regarding high SBP and high LDL-attributed rates based on geographical locations. Eventually, a cause-specific analysis marked CVDs and DM/KDs as the main causes of mortality attributed to these MRFs.

By 2017, deaths due to CVDs and DM among the Iranian population aged 45 or older comprised the largest number of deaths. This necessitated the control of high SBP, FPG, BMI, and LDL to prevent a large proportion of death among adults in Iran (9, 21). In addition, other studies in Iran found that high FPG, BMI, and total cholesterol were responsible for approximately one-third to one-half of high SBP-attributed deaths (3, 22). Previous studies reported that 26.4% of adults were dealing with high SBP worldwide, which is expected to increase to 29.2% by 2025 globally, accounting for 10.4 million (9.39–11.5) deaths and 218 million (198–237) DALYs worldwide in 2017 (23). In addition, among Iranians aged 25–70 years, 24.1% were facing high SBP in 2011 (24). The results from the National and Subnational Burden of Diseases (NASBOD) Study in 2016 demonstrated that from 1990 to 2016, the number of adults with high SBP increased from 1.8 to 13.6 million and the national age-standardized prevalence of hypertension increased from 8.7 to 28.8% in women, and from 8.0 to 28.8% in men. Moreover, the mean systolic and diastolic blood pressures have shown similar increasing trends. In contrast, our study showed that the age-standardized death rates due to high SBP decreased by−35.6% in a quite similar time period (25). The reduction in SBP-attributed death rates (despite the increase in prevalence) is a result of efforts conducted according to the National Action Plan on reducing mortality attributed to non-communicable diseases. Target 4 of this action plan was to reduce average salt intake by 4.3% in the population via education and long-term health policies (2).

High LDL cholesterol, another major risk factor for atherosclerosis and CVDs, also had decreasing trends regarding attributed death and DALY age-standardized rates. Recent studies reported that serum total cholesterol has reduced in adult Iranian individuals over the past decade; however, improvement in other serum lipoprotein levels was not significant and still needs to be managed in the population, especially regarding LDL (1, 26). Over the last decade, worldwide morbidity and mortality attributable to high LDL have increased by 26.9 and 28.0%, respectively (27). The results of our study showed that death and DALY rates significantly decreased over time from 1990 to 2019 by age. Death and DALY rates increased considerably in both sexes with the highest rate estimated for the over 80 years group, and women had higher rates than men over 75 years. Concerning related causes, CVDs were the only cause with the highest proportion in the case of YLLs and YLDs. However, both rates due to CVDs decreased over time from 1990 to 2019. Similarly, according to results from NASBOD Study, the occurrence of deaths due to CVDs has shown decreasing trend from 2001 to 2015 at both national and subnational levels (28). The decrease in death rates due to CVDs attributable to high LDL is concordant with Iranian National Action Plan Targets on NCDs and SDGs. These targets include using zero trans fatty acids in food and oily products and a 1.25% relative reduction in premature deaths from CVDs (2).

High BMI, in contrast, has also become a common health issue all over the world. Based on previous reports, the age-standardized rate of high BMI-attributed deaths remained stable for female subjects, and increased by 14.5% only for male subjects. Moreover, the global age-standardized rate of DALYs increased by 12.7% for female subjects and 26.8% for male subjects (29). According to the findings of STEPS survey in 2016, the prevalence of normal weight, obesity, and overweight among Iranian adults was 36.7, 22.7, and 59.3%, respectively. In that study, high BMI was significantly more prevalent among women; the 55–64 and the 18–24 age groups had the highest and lowest prevalence of high BMI, respectively. In addition, the mentioned study found a geographical pattern at the subnational level of Iran, where the level of BMI increased among the population from the southeastern to the northwestern regions (30). Furthermore, according to the recently published results of Iran STEPS Survey 2021, the national prevalence of normal weight, obesity, and overweight/obesity in Iranian adults was estimated at about 33.61, 24.96, and 63.02%, respectively (31, 32). The recent results from NASBOD on obesity showed that, from 1990 to 2016 the mean BMI, and the age-standardized prevalence of overweight has increased (33). Moreover, the prevalence of obesity at the national scale has faced a 3-fold increase during this period. In 2016, the age-standardized prevalence rates of obesity were 40.4% in female population and 35.0% in male population. Considering the mentioned pattern in prevalence and deaths attributed to BMI, the SDGs seem far from being achieved in due time. This issue urges the need for immediate action and strategies for further control of BMI in the population of Iran (33).

DM resulting from high FPG is among the leading causes associated with considerable DALY rates among NCDs in Iran, with 767,461 DALYs being exerted to the healthcare system in 2016 (34). Due to the increasing trends in prevalence and DALYs attributed to diabetes, many nations are taking possible actions to address its health and financial burden (35, 36). Based on our study, the rates slightly increased over the period. Concerning geographical disparities, western and southeastern provinces had lower death and DALY rates. Considering the National action plan, the first target is a 1.25% relative reduction in the risk of premature deaths due to DM. This target is concordant with the SDGs target by 2030 to reduce by one-third premature mortality from NCDs through prevention and treatment (2).

Considering SDI quintiles, we found similar patterns regarding high SBP, high BMI, and high LDL-attributed death and DALY rates, in which the middle SDI region had the highest rates; the rates decreased by raising or lowering the SDI level. Moreover, between 1990 and 2019, the death rates of all SDI quintiles decreased. However, high FPG rates revealed a distinct pattern that middle, high-middle, and high-SDI regions had jointly higher rates than low and low-middle SDI quintiles so a positive association between the rates and SDI levels was observed. A recent study evaluating the association between SDI and high-BMI-attributed DALYs at a global scale also suggested that the lowest age-standardized DALY rates were found in the low-SDI quintile and high-SDI quintile, and over time, age-standardized DALY rates increased in low-SDI regions, but decreased in regions with the highest SDI (29). The progress in controlling the burden of these risks is achievable by increasing SDI through enhancing education and gross domestic product (GDP) per capita. Policies and national programs alongside socioeconomic developments play a prominent role in this process (9). During recent decades, the socio-economic development and improved primary healthcare acted by increasing SDI and decreasing deaths attributed to these risk factors, particularly in the low-SDI regions; these actions are so-called revolutionary in healthcare in Iran (2). Concerning geographical disparities, high FPG and high BMI-attributed deaths and DALY rates showed the highest discrepancy among provinces. This discrete pattern could be explained by the fact that Iran is a vast country with a diverse population consisting of various ethnicities and lifestyles which might affect their susceptibility to MRFs and related diseases (4). In addition, some regions might not have proper access to healthcare and laboratory facilities to perform routine check-ups and workups, which might result in underdiagnosis of potential risk factors and diseases.

Many challenges exist on the path of controlling the burden of MRFs in Iran. Currently, Iran is dealing with an epidemiological transitional period making it a vital region experiencing an alarming increase in the burden of NCDs (3). MRFs are critical risk factors associated with NCDs which are mostly modifiable through screening, education, and treatment. Healthy lifestyle habits, such as smoking cessation, physical exercise, and a healthy dietary plan could affect these risk factors (2, 3, 24, 37). For instance, reducing the amount of salt intake can be useful to reduce the SBP level in the population (3, 38, 39). As Iranians consume a noticeable amount of bread in their diet, cutting on the salt in the process of bread might be a step toward modifying salt intake along with raising the general awareness (38). Mass education and evidence-based healthcare programs could also effectively help to control behavioral habits and to reduce the negative outcomes of MRFs (24). Thus, early education, especially among youngsters about healthy diet, physical activity, and symptoms of disorders related to MRFs, mainly DM and hypertension, along with well-timed screening and treatment may help to limit the burden of these risk factors among the population. Considering high FPG, the quality of treatment was previously found to be effective in limiting attributed DALYs and appropriate control of plasma glucose could prevent possible complications (35). Developing a proper plan for screening, routine workups, and on-time treatment initiation by clinicians may also benefit the burden of MRFs. Considering high LDL, using statins for individuals with abnormal values can reduce the amount of total cholesterol and its subtypes, thus it can be useful for reducing the burden of its complications (1). In terms of high BMI, community-based education aiming at lifestyle modification is the key measure, including physical activity and healthy diet which have been found to be beneficial in reducing high BMI-attributed burden (40). With regards to the burden of MRFs, policymakers could investigate the results of the present study to design and utilize proper actions to achieve better MRFs management aiming at expanding equity among the population, diminishing disparities, preventing diseases, and consequently improving the health status of the Iranian population. The practical implication of the findings of the current study could be lending a hand to health policymakers by providing a big picture of the status of MRFs' burden at the national and subnational levels. Despite utilizing various effective methods in the 2019 GBD study to analyze and integrate the results of several studies, this study had some limitations. First, the difference in the selection of samples in previous studies and surveys might bias the final results. Moreover, differences in laboratory facilities that were applied for measuring these risk factors across the nation in different time periods may lead to mislabeling the population to some extent. In addition, many developing regions as well as Iran lack a systematic registry program for causes of deaths, which could limit results around attributed deaths by causes since data obtained from registry systems play a pivotal role in the GBD study. All limitations around the 2019 GBD study would also be applicable to our study including the data scarcity in some regions as this study has subnational data reported from Iran causing biased results estimated for regions with limited data (9). Considering all these limitations, the presented data provide a comprehensive image of the burden of MRFs in Iran and facilitate the path for researchers and health authorities to expand the knowledge in the field with a final goal of controlling of the burden of NCDs in the country.

## 5. Conclusion

The present study revealed that the burden of four metabolic risk factors had distinct patterns over the study period. The age-standardized rates of death and DALY remarkably decreased regarding high SBP and high LDL; in contrast, the burden attributed to high FPG and high BMI had slightly accelerated. The highest SBP attribute burden was constantly higher than the others from 1990 to 2019. We also found disparities regarding different subnational regions and sex and age groups for each risk factor and related causes of diseases. These findings could provide policymakers and health authorities with a clearer vision guiding them toward more appropriate decision-making to prevent the burden of metabolic risk factors and related disorders as well as rational and proper allocation of resources among the population in the future.

## Data availability statement

Publicly available datasets were analyzed in this study. This data can be found here: https://vizhub.healthdata.org/gbd-results/.

## Ethics statement

The studies involving human participants were reviewed and approved by Endocrinology and Metabolism Research Institute at Tehran University of Medical Sciences (IR.TUMS.EMRI.REC.1400.026). Written informed consent for participation was not required for this study in accordance with the national legislation and the institutional requirements.

## Author contributions

Please see Appendix (pp 69–70) for more detailed information about individual author contributions to the research, divided into the following categories: providing data or critical feedback on data sources, developing methods or computational machinery. providing critical feedback on methods or results, drafting the manuscript or revising it critically for important intellectual content, and management of the overall research enterprise. Members of the core research team for this topic area had full access to the underlying data used to generate estimates presented in this article. All other authors had access to and reviewed estimates as part of the research evaluation process, which includes additional stages of formal review.

## GBD 2019 Iran Metabolic Risk Factors Collaborators

Soroush Moradi^1^, Amirhossein Parsaei^1^, Sahar Saeedi Moghaddam^1^, Armin Aryannejad^1, 2^, Sina Azadnajafabad^1^, Negar Rezaei^1, 3^, Baharnaz Mashinchi^1, 2^, Zahra Esfahani^1^, Parnian Shobeiri^1, 4^, Nazila Rezaei^1^, Amirali Aali^5^, Mohsen Abbasi-Kangevari^1^, Zeinab Abbasi-Kangevari^1, 6^, Shima Abdollahi^7^, Abdorrahim Absalan^8, 9^, Siamak Afaghi^10^, Ali Ahmadi^11, 12^, Amir Moghadam Ahmadi^13, 14^, Sepideh Ahmadi^15^, Marjan Ajami^16, 17^, Meisam Akhlaghdoust^18, 19^, Sudabeh Alatab^20^, Yousef Alimohamadi^21^, Mehrdad Amir-Behghadami^22, 23^, Sohrab Amiri^24^, Davood Anvari^25, 26^, Jalal Arabloo^27^, Elaheh Askari^28^, Seyyed Shamsadin Athari^29^, Abolfazl Avan^30^, Samad Azari^31^, Hassan Babamohamadi^32^, Nayereh Baghcheghi^33^, Sara Bagherieh^34^, Prof Hamid Reza Baradaran^35, 36^, Azadeh Bashiri^37^, Mostafa Dianatinasab^38, 39^, Shirin Djalalinia^40^, Milad Dodangeh^41^, Mahsa Dolatshahi^42^, Sareh Edalati^43^, Hossein Farrokhpour^1, 4^, Ali Fatehizadeh^44^, Fataneh Ghadirian^45^, Ahmad Ghashghaee^46^, Ali Gholami^47, 48^, Pouya Goleij^49^, Nima Hafezi-Nejad^4, 50^, Hamidreza Hasani^51, 52^, Soheil Hassanipour^53, 54^, Mahsa Heidari-Foroozan^1, 55^, Kamran Hessami^56, 57^, Kaveh Hosseini^58, 59^, Mohammad-Salar Hosseini^60^, Seyed Kianoosh Hosseini^61^, Soodabeh Hoveidamanesh^62^, Farideh Iravanpour^63^, Rana Irilouzadian^55, 64^, Zahra Jamalpoor^65^, Tannaz Jamialahmadi^66, 67^, Ali Kabir^68^, Neda Kaydi^69^, Sina Kazemian^70, 71^, Mohammad Keykhaei^1, 72^, Morteza Abdullatif Khafaie^73^, Shaghayegh Khanmohammadi^1, 4^, Sorour Khateri^74^, Farzad Kompani^75^, Hamid Reza Koohestani^76^, Prof Soleiman Mahjoub^77, 78^, Prof Ata Mahmoodpoor^79^, Marzieh Mahmoudimanesh^80^, Prof Elaheh Malakan Rad^81^, Mohammad-Reza Malekpour^1^, Prof Reza Malekzadeh^20, 82^, Mohammad Ali Mansournia^83^, Abdoljalal Marjani^84^, Esmaeil Mohammadi^1, 4^, Sara Momtazmanesh^1, 4^, Maryam Moradi^85^, Maziar Moradi-Lakeh^86^, Farhad Moradpour^87^, Negar Morovatdar^88^, Seyed Aria Nejadghaderi^1, 55^, Maryam Noori^89^, Prof Hasti Nouraei^90^, Ali Nowroozi^4^, Hassan Okati-Aliabad^91^, Prof Akram Pourshams^20^, Mehran Rahimi^92^, Shayan Rahmani^1, 55^, Vahid Rahmanian^93^, Sina Rashedi^1, 59^, Mohammad-Mahdi Rashidi^1, 6^, Iman Razeghian-Jahromi^94^, Malihe Rezaee^95, 58^, Leila Sabzmakan^96^, Erfan Sadeghi^97^, Prof Masoumeh Sadeghi^98^, Saeid Sadeghian^99^, Amirhossein Sahebkar^100, 101^, Hamideh Salimzadeh^20^, Saman Sargazi^102^, Prof Nizal Sarrafzadegan^103, 104^, Sadaf G Sepanlou^20, 82^, Melika Shafeghat^4^, Mahan Shafie^105^, Ataollah Shahbandi^4^, Fariba Shahraki-Sanavi^91^, Mehran Shams-Beyranvand^106^, Athena Sharifi-Razavi^107^, Seyed Afshin Shorofi^108, 109^, Seyed-Amir Tabatabaeizadeh^110^, Alireza Tahamtan^111^, Majid Taheri^112, 113^, Seyed Mohammad Vahabi^4^, Prof Siavash Vaziri^114^, Fereshteh Yazdanpanah^115, 116^, Mazyar Zahir^117^, Moein Zangiabadian^55^, Iman Zare^118^, Zahra Zareshahrabadi^90^, Prof Mohsen Naghavi^119, 120^, Prof Bagher Larijani^3^, Prof Farshad Farzadfar^1, 3^

## Affiliations

^1^Non-Communicable Diseases Research Center, Endocrinology and Metabolism Population Sciences Institute, Tehran University of Medical Sciences, Tehran, Iran

^2^Experimental Medicine Research Center, Tehran University of Medical Sciences, Tehran, Iran

^3^Endocrinology and Metabolism Research Center, Endocrinology and Metabolism Clinical Sciences Institute, Tehran University of Medical Sciences, Tehran, Iran

^4^School of Medicine, Tehran University of Medical Sciences, Tehran, Iran

^5^Faculty of Medicine, Mashhad University of Medical Sciences, Mashhad, Iran

^6^Social Determinants of Health Research Center, Shahid Beheshti University of Medical Sciences, Tehran, Iran

^7^Department of Nutrition, North Khorasan University of Medical Sciences, Bojnurd, Iran

^8^Department of Medical Laboratory Sciences, Khomein University of Medical Sciences, Khomein, Iran

^9^Department of Research and Development, Satras Biotechnology Company, Tehran, Iran

^10^Department of Internal Medicine, Shahid Beheshti University of Medical Sciences, Tehran, Iran

^11^Department of Epidemiology and Biostatistics, Shahrekord University of Medical Sciences, Shahrekord, Iran

^12^Department of Epidemiology, Shahid Beheshti University of Medical Sciences, Tehran, Iran

^13^Department of Neurology, Rafsanjan University of Medical Sciences, Rafsanjan, Iran

^14^Non-Communicable Diseases Research Center, Rafsanjan University of Medical Sciences, Rafsanjan, Iran

^15^School of Advanced Technologies in Medicine, Shahid Beheshti University of Medical Sciences, Tehran, Iran

^16^Department of Food and Nutrition Policy and Planning Research, National Institute of Nutrition, Tehran, Iran

^17^National Nutrition and Food Technology Research Institute, Shahid Beheshti University of Medical Sciences, Tehran, Iran

^18^Functional Neurosurgery Research Center, Shahid Beheshti University of Medical Sciences, Tehran, Iran

^19^International Federation of Inventors' Associations, Geneva, Eswatini

^20^Digestive Diseases Research Institute, Tehran University of Medical Sciences, Tehran, Iran

^21^Health Research Center, Baqiyatallah University of Medical Sciences, Tehran, Iran

^22^Road Traffic Injury Research Center, Tabriz University of Medical Sciences, Tabriz, Iran

^23^Department of Health Service Management, Iranian Center of Excellence in Health Management, Tabriz, Iran

^24^Quran and Hadith Research Center, Baqiyatallah University of Medical Sciences, Tehran, Iran

^25^Department of Parasitology, Mazandaran University of Medical Sciences, Sari, Iran

^26^Department of Parasitology, Iranshahr University of Medical Sciences, Iranshahr, Iran

^27^Health Management and Economics Research Center, Iran University of Medical Sciences, Tehran, Iran

^28^Department of Nutrition, Lorestan University of Medical Sciences, Khorramabad, Iran

^29^Department of Immunology, Zanjan University of Medical Sciences, Zanjan, Iran

^30^Department of Public Health, Mashhad University of Medical Sciences, Mashhad, Iran

^31^Hospital Management Research Center, Iran University of Medical Sciences, Tehran, Iran

^32^Department of Nursing, Semnan University of Medical Sciences and Health Services, Semnan, Iran

^33^Department of Nursing, Saveh University of Medical Sciences, Saveh, Iran

^34^School of Medicine, Isfahan University of Medical Sciences, Isfahan, Iran

^35^Department of Epidemiology, Iran University of Medical Sciences, Tehran, Iran

^36^Ageing Clinical & Experimental Research (ACER), Institute of Applied Health Sciences, University of Aberdeen, Aberdeen, United Kingdom

^37^Department of Health Information Management, Shiraz University of Medical Sciences, Shiraz, Iran

^38^Department of Epidemiology, Maastricht University, Maastricht, Netherlands

^39^Department of Epidemiology, Shiraz University of Medical Sciences, Shiraz, Iran

^40^Development of Research and Technology Center, Ministry of Health and Medical Education, Tehran, Iran

^41^School of Medicine, Iran University of Medical Sciences, Tehran, Iran

^42^Department of Radiology, Tehran University of Medical Sciences, Saint Louis, Iran

^43^Department of Community Nutrition, Shahid Beheshti University of Medical Sciences, Tehran, Iran

^44^Department of Environmental Health Engineering, Isfahan University of Medical Sciences, Isfahan, Iran

^45^Psychiatric Nursing and Management Department, Shahid Beheshti University of Medical Sciences, Tehran, Iran

^46^School of Public Health, Qazvin University of Medical Sciences, Qazvin, Iran

^47^Department of Epidemiology and Biostatistics, Neyshabur University of Medical Sciences, Neyshabur, Iran

^48^Non-Communicable Diseases Research Center, Neyshabur University of Medical Sciences, Neyshabur, Iran

^49^Department of Genetics, Sana Institute of Higher Education, Sari, Iran

^50^Department of Radiology and Radiological Science, Johns Hopkins University, Baltimore, MD, United States

^51^Department of Ophthalmology, Iran University of Medical Sciences, Karaj, Iran

^52^Ophthalmic Research Center, Tehran, Iran

^53^Gastrointestinal and Liver Diseases Research Center, Guilan University of Medical Sciences, Rasht, Iran

^54^Caspian Digestive Disease Research Center, Guilan University of Medical Sciences, Rasht, Iran

^55^School of Medicine, Shahid Beheshti University of Medical Sciences, Tehran, Iran

^56^Maternal Fetal Care Center, Harvard University, Boston, MA, United States

^57^Maternal Fetal Medicine Research Center, Shiraz University of Medical Sciences, Shiraz, Iran

^58^Tehran Heart Center, Tehran University of Medical Sciences, Tehran, Iran

^59^Department of Cardiology, Tehran University of Medical Sciences, Tehran, Iran

^60^Student Research Committee, Tabriz University of Medical Sciences, Tabriz, Iran

^61^Department of Interventional Cardiology, Hamedan University of Medical Sciences, Hamadan, Iran

^62^Burn Research Center, Shahid Motahari Hospital, Tehran, Iran

^63^Shiraz Neuroscience Research Center, Shiraz University of Medical Sciences, Shiraz, Iran

^64^Burn Research Center, Iran University of Medical Sciences, Tehran, Iran

^65^Trauma Research Center, Aja University of Medical Sciences, Tehran, Iran

^66^Department of Nutrition, Mashhad University of Medical Sciences, Mashhad, Iran

^67^Department of Food Science and Technology, Islamic Azad University, Quchan, Iran

^68^Minimally Invasive Surgery Research Center, Iran University of Medical Sciences, Tehran, Iran

^69^Environmental Health Department, Ahvaz Jundishapur University of Medical Sciences, Ahvaz, Iran

^70^Cardiac Primary Prevention Research Center, Tehran University of Medical Sciences, Tehran, Iran

^71^Department of Cardiac Electrophysiology, Tehran Heart Center, Tehran University of Medical Sciences, Tehran, Iran

^72^Students' Scientific Research Center (SSRC), Tehran University of Medical Sciences, Tehran, Iran

^73^Social Determinants of Health Research Center, Ahvaz Jundishapur University of Medical Sciences, Ahvaz, Iran

^74^School of Medicine, Kurdistan University of Medical Sciences, Sanandaj, Iran

^75^Children's Medical Center, Tehran University of Medical Sciences, Tehran, Iran

^76^Social Determinants of Health Research Center, Saveh University of Medical Sciences, Saveh, Iran

^77^Cellular and Molecular Biology Research Center, Babol University of Medical Sciences, Babol, Iran

^78^Department of Clinical Biochemistry, Babol University of Medical Sciences, Babol, Iran

^79^Department of Anesthesiology and Critical Care, Tabriz University of Medical Sciences, Tabriz, Iran

^80^Department of Biostatistics and Epidemiology, Kerman University of Medical Sciences, Kerman, Iran

^81^Department of Pediatric Cardiology, Tehran University of Medical Sciences, Tehran, Iran

^82^Non-Communicable Diseases Research Center, Shiraz University of Medical Sciences, Shiraz, Iran

^83^Department of Epidemiology and Biostatistics, Tehran University of Medical Sciences, Tehran, Iran

^84^Department of Biochemistry, Golestan University of Medical Sciences, Gorgan, Iran

^85^Iran University of Medical Sciences, Tehran, Iran

^86^Preventive Medicine and Public Health Research Center, Iran University of Medical Sciences, Tehran, Iran

^87^Social Determinants of Health Research Center, Kurdistan University of Medical Sciences, Sanandaj, Iran

^88^Clinical Research Development Unit, Mashhad University of Medical Sciences, Mashhad, Iran

^89^Student Research Committee, Iran University of Medical Sciences, Tehran, Iran

^90^Department of Medical Mycology and Parasitology, Shiraz University of Medical Sciences, Shiraz, Iran

^91^Health Promotion Research Center, Zahedan University of Medical Sciences, Zahedan, Iran

^92^Cardiovascular Research Center, Tabriz University of Medical Sciences, Tabriz, Iran

^93^Department of Community Medicine, Jahrom University of Medical Sciences, Jahrom, Iran

^94^Cardiovascular Research Center, Shiraz University of Medical Sciences, Shiraz, Iran

^95^Pharmacology Department, Shahid Beheshti University of Medical Sciences, Tehran, Iran

^96^Non-Communicable Diseases Research Center, Alborz University of Medical Sciences, Karaj, Iran

^97^Research Consultation Center (RCC), Shiraz University of Medical Sciences, Shiraz, Iran

^98^Cardiac Rehabilitation Research Center, Isfahan University of Medical Sciences, Isfahan, Iran

^99^Department of Pediatric Neurology, Ahvaz Jundishapur University of Medical Sciences, Ahvaz, Iran

^100^Applied Biomedical Research Center, Mashhad University of Medical Sciences, Mashhad, Iran

^101^Biotechnology Research Center, Mashhad University of Medical Sciences, Mashhad, Iran

^102^Department of Biochemistry, Zahedan University of Medical Sciences, Zahedan, Iran

^103^Isfahan Cardiovascular Research Institute, Isfahan University of Medical Sciences, Isfahan, Iran

^104^School of Population and Public Health, University of British Columbia, Vancouver, BC, Canada

^105^Department of Neurology, Tehran University of Medical Sciences, Tehran, Iran

^106^School of Medicine, Alborz University of Medical Sciences, Karaj, Iran

^107^Department of Neurology, Mazandaran University of Medical Sciences, Sari, Iran

^108^Department of Medical-Surgical Nursing, Mazandaran University of Medical Sciences, Sari, Iran

^109^Department of Nursing and Health Sciences, Flinders University, Adelaide, SA, Australia

^110^Department of Nutrition Sciences, Varastegan Institute for Medical Sciences, Mashhad, Iran

^111^Department of Microbiology, Golestan University of Medical Sciences, Gorgan, Iran

^112^Trauma and Injury Research Center, Iran University of Medical Sciences, Tehran, Iran

^113^Medical Ethics and Law Research Center, Shahid Beheshti University of Medical Sciences, Tehran, Iran

^114^Department of Infectious Disease, Kermanshah University of Medical Sciences, Kermanshah, Iran

^115^Department of Pediatric Allergy and Immunology, Tabriz University of Medical Sciences, Tabriz, Iran

^116^Department of Pediatric Allergy and Immunology, Tehran University of Medical Sciences, Tehran, Iran

^117^Urology and Nephrology Research Center, Shahid Beheshti University of Medical Sciences, Tehran, Iran

^118^Research and Development Department, Sina Medical Biochemistry Technologies, Shiraz, Iran

^119^Institute for Health Metrics and Evaluation, University of Washington, Seattle, WA, United States

^120^Department of Health Metrics Sciences, School of Medicine, University of Washington, Seattle, WA, United States

## Supplementary material

Supplementary Figure 1 Age-standardized death **(A)** and DALY **(B)** rates attributable to metabolic risk factors including high SBP, high FPG, high BMI, and high LDL (per 100,000 person-year) based on rankings in Iran and its 31 provinces in 1990 and 2019, for both sexes.

Supplementary Figure 2 Time trends of age-standardized YLL **(A)** and YLD **(B)** rates (per 100,000 person-year) attributable to metabolic risk factors including high SBP, high FPG, high BMI, and high LDL by year and sex with 95% uncertainty interval from 1990 to 2019.

Supplementary Figure 3 Age-standardized YLL **(A)** and YLD **(B)** rates (per 100,000 person-year) attributable to metabolic risk factors including high SBP, high FPG, high BMI, and high LDL in 31 provinces of Iran in 1990 and 2019 for both sexes.

Supplementary Figure 4 Age-standardized YLL and YLD rates (per 100,000 person-year) attributable to metabolic risk factors including high SBP **(A)**, high FPG **(B)**, high BMI **(C)**, and high LDL **(D)** in 1990 and 2019 for both sexes (left: for Iran and its 31 provinces; right: based on age groups).

Supplementary Figure 5 Age-standardized YLL **(A)** and YLD **(B)** Bill & Melinda Gates Foundation rates (per 100,000 person-year) attributable to metabolic risk factors including high SBP, high FPG, high BMI, and high LDL by 22 main causes of deaths in 1990 and 2019 for both sexes.

Supplementary Table 1 Data sources of high SBP in the GBD study 2019 regarding surveys, scientific literature, reports, and cross-sectional studies conducted in Iran from 1990 to 2019.

Supplementary Table 2 Data sources of high FPG in the GBD study 2019 regarding surveys, scientific literature, reports, and cross-sectional studies conducted in Iran from 1990 to 2019.

Supplementary Table 3 Data sources of high BMI in the GBD study 2019 regarding surveys, scientific literature, reports, and cross-sectional studies conducted in Iran from 1990 to 2019.

Supplementary Table 4 Data sources of high LDL in the GBD study 2019 regarding surveys, scientific literature, reports, and cross-sectional studies conducted in Iran from 1990 to 2019.

Supplementary Table 5 Age-standardized death, DALY, YLL, and YLD rates attributable to high SBP (per 100,000 person-year) and percent changes between 1990 and 2019 for male subjects, female subjects, and both, at the subnational level.

Supplementary Table 6 Age-standardized death, DALY, YLL, and YLD rates attributable to high FPG (per 100,000 person-year) and percent changes between 1990 and 2019 for male subjects, female subjects, and both, at the subnational level.

Supplementary Table 7 Age-standardized death, DALY, YLL, and YLD rates attributable to high BMI (per 100,000 person-year) and percent changes between 1990 and 2019 for male subjects, female subjects, and both, at the subnational level.

Supplementary Table 8 Age-standardized death, DALY, YLL, and YLD rates attributable to high LDL (per 100,000 person-year) and percent changes between 1990 and 2019 for male subjects, female subjects, and both, at the subnational level.

## References

[1] Aryan Z Mahmoudi N Sheidaei A Rezaei S Mahmoudi Z Gohari K. The prevalence, awareness, and treatment of lipid abnormalities in Iranian adults: surveillance of risk factors of noncommunicable diseases in Iran 2016. J Clin Lipidol. (2018) 12:1471–81. e4. 10.1016/j.jacl.2018.08.001 30195823

[2] Peykari N Hashemi H Dinarvand R Haji-Aghajani M Malekzadeh R Sadrolsadat A. National action plan for non-communicable diseases prevention and control in Iran; a response to emerging epidemic. J Diabetes Metab Disord. (2017) 16:1–7. 10.1186/s40200-017-0288-4 28127543 PMC5260033

[3] Farzadfar F Danaei G Namdaritabar H Rajaratnam JK Marcus JR Khosravi A. National and subnational mortality effects of metabolic risk factors and smoking in Iran: a comparative risk assessment. Popul Health Metr. (2011) 9:1–11. 10.1186/1478-7954-9-55 21989074 PMC3229448

[4] Danaei G Farzadfar F Kelishadi R Rashidian A Rouhani OM Ahmadnia S. Iran in transition. Lancet. (2019) 393:1984–2005. 10.1016/S0140-6736(18)33197-0 31043324

[5] GBD 2019 Iran Collaborators. Health system performance in Iran: a systematic analysis for the Global Burden of Disease Study 2019. Lancet. (2022) 399:1625–45. 10.1016/s0140-6736(21)02751-3 35397236 PMC9023870

[6] Koolaji S Sharifnejad Tehrani Y Azadnajafabad S Saeedi Moghaddam S Shahin S Ghamari A. A 30-year trend of ischemic heart disease burden in a developing country; a systematic analysis of the global burden of disease study 2019 in Iran. Int J Cardiol. (2023) 379:127–33. 10.1016/j.ijcard.2023.03.012 36898585

[7] Fallahzadeh A Esfahani Z Sheikhy A Keykhaei M Moghaddam SS Tehrani YS. National and subnational burden of stroke in Iran from 1990 to 2019. Ann Clin Transl Neurol. (2022) 9:669–83. 10.1002/acn3.51547 35395141 PMC9082377

[8] Mousavi SF Peimani M Moghaddam SS Tabatabaei-Malazy O Ghasemi E Shobeiri P. National and subnational survey on diabetes burden and quality of care index in Iran: a systematic analysis of the global burden of disease study 1990-2019. J Diabetes Metab Disord. (2022) 21:1599–608. 10.1007/s40200-022-01108-x 36404869 PMC9672253

[9] Murray CJ Aravkin AY Zheng P Abbafati C Abbas KM Abbasi-Kangevari M. Global burden of 87 risk factors in 204 countries and territories, 1990–2019: a systematic analysis for the Global Burden of Disease Study 2019. Lancet. (2020) 396:1223–49. 10.1016/S0140-6736(20)30752-2 33069327 PMC7566194

[10] Azadnajafabad S Mohammadi E Aminorroaya A Fattahi N Rezaei S Haghshenas R. Non-communicable diseases' risk factors in Iran; a review of the present status and action plans. J Diabetes Metab Disord. (2021) 1–9. 10.1007/s40200-020-00709-8 33500879 PMC7821170

[11] Afshin A Micha R Khatibzadeh S Fahimi S Shi P Powles J. The impact of dietary habits and metabolic risk factors on cardiovascular and diabetes mortality in countries of the Middle East and North Africa in 2010: a comparative risk assessment analysis. BMJ Open. (2015) 5:e006385. 10.1136/bmjopen-2014-006385 25995236 PMC4442236

[12] Ramezankhani A Azizi F Hadaegh F. Gender differences in changes in metabolic syndrome status and its components and risk of cardiovascular disease: a longitudinal cohort study. Cardiovasc Diabetol. (2022) 21:227. 10.1186/s12933-022-01665-8 36324143 PMC9632145

[13] Balali P Nasserinejad M Azadnajafabad S Ahmadi N Delavari F Rashidian L. Is elevated ALT associated with lifestyle risk factors? A population-based survey. J Diabetes Metab Disord. (2022). 10.1007/s40200-022-01137-6 36404851 PMC9672187

[14] Mehran L Honarvar M Masoumi S Khalili D Amouzegar A Azizi F. Weight fluctuation, mortality, and cardiovascular disease in adults in 18 years of follow-up: Tehran Lipid and Glucose Study. J Endocrinol Invest. (2023) 46:37–49. 10.1007/s40618-022-01881-9 35921037

[15] Stevens GA Alkema L Black RE Boerma JT Collins GS Ezzati M. Guidelines for accurate and transparent health estimates reporting: the GATHER statement. Lancet. (2016) 388:e19–23. 10.1016/S0140-6736(16)30388-9 27371184

[16] GBD 2019 Diseases and Injuries Collaborators. Global burden of 369 diseases and injuries in 204 countries and territories, 1990-2019: a systematic analysis for the Global Burden of Disease Study 2019. Lancet. (2020) 396:1204–22. 10.1016/s0140-6736(20)30925-9 33069326 PMC7567026

[17] Institute for Health Metrics Evaluation (IHME). GBD Compare Data Visualization. Seattle, WA: IHME, University of Washington (2020). Available online at: http://vizhub.healthdata.org/gbd-compare (accessed January 20, 2023).

[18] Institute for Health Metrics Evaluation. GBD Compare. University of Washington (2021). Available online at: https://vizhub.healthdata.org/gbd-compare/ (accessed January 20, 2023).

[19] Cole TJ Bellizzi MC Flegal KM Dietz WH. Establishing a standard definition for child overweight and obesity worldwide: international survey. BMJ. (2000) 320:1240–3. 10.1136/bmj.320.7244.1240 10797032 PMC27365

[20] GBD 2019 Demographics Collaborators. Global age-sex-specific fertility, mortality, healthy life expectancy (HALE), and population estimates in 204 countries and territories, 1950-2019: a comprehensive demographic analysis for the Global Burden of Disease Study 2019. Lancet. (2020) 396:1160–203. 10.1016/s0140-6736(20)30977-6 33069325 PMC7566045

[21] Mokdad AH Mensah GA Krish V Glenn SD Miller-Petrie MK Lopez AD. Global, regional, national, and subnational big data to inform health equity research: perspectives from the Global Burden of Disease Study 2017. Ethn Dis. (2019) 29:159. 10.18865/ed.29.S1.159 30906165 PMC6428171

[22] Keykhaei M Rezaei N Roshani S Montazeri F Nasserinejad M Azadnajafabad S. Population attributable fraction estimates of cardiovascular diseases in different blood pressure levels in a large-scale cross-sectional study: a focus on prevention strategies and treatment coverage. Blood Press Monit. (2023) 28:1–10. 10.1097/MBP.0000000000000612 36606475

[23] Gakidou E Afshin A Abajobir AA Abate KH Abbafati C Abbas KM. Global, regional, and national comparative risk assessment of 84 behavioural, environmental and occupational, and metabolic risks or clusters of risks, 1990–2016: a systematic analysis for the Global Burden of Disease Study 2016. Lancet. (2017) 390:1345–422. 10.1016/S0140-6736(17)32366-8 28919119 PMC5614451

[24] Mahdavi M Parsaeian M Mohajer B Modirian M Ahmadi N Yoosefi M. Insight into blood pressure targets for universal coverage of hypertension services in Iran: the 2017 ACC/AHA versus JNC 8 hypertension guidelines. BMC Public Health. (2020) 20:1–9. 10.1186/s12889-020-8450-1 32183754 PMC7076938

[25] Sepanlou SG Mehdipour P Ghanbari A Djalalinia S Peykari N Kasaeian A. Levels and trends of hypertension at National and Subnational Scale in Iran from 1990 to 2016: a systematic review and pooled analysis. Arch Iran Med. (2021) 24:306–16. 10.34172/aim.2021.43 34196191

[26] Azadnajafabad S Karimian M Roshani S Rezaei N Mohammadi E Saeedi Moghaddam S. Population attributable fraction estimates of cardiovascular diseases in different levels of plasma total cholesterol in a large-scale cross-sectional study: a focus on prevention strategies and treatment coverage. J Diabetes Metab Disord. (2020) 19:1453–63. 10.1007/s40200-020-00673-3 33520846 PMC7843742

[27] Forouzanfar MH Alexander L Anderson HR Bachman VF Biryukov S Brauer M. Global, regional, and national comparative risk assessment of 79 behavioural, environmental and occupational, and metabolic risks or clusters of risks in 188 countries, 1990-2013: a systematic analysis for the Global Burden of Disease Study 2013. Lancet. (2015) 386:2287–323. 10.1016/S0140-6736(15)00128-2 26364544 PMC4685753

[28] Djalalinia S Saeedi Moghaddam S Rezaei N Rezaei N Mansouri A Abdolhamidi E. National and sub-national patterns of mortality from stroke in the Iranian population (1990–2015): Complementary results from the NASBOD study. Int J Stroke. (2020) 15:132–48. 10.1177/1747493018799974 30226449

[29] Dai H Alsalhe TA Chalghaf N Riccò M Bragazzi NL Wu J. The global burden of disease attributable to high body mass index in 195 countries and territories, 1990–2017: an analysis of the Global Burden of Disease Study. PLoS Med. (2020) 17:e1003198. 10.1371/journal.pmed.1003198 32722671 PMC7386577

[30] Djalalinia S Saeedi Moghaddam S Sheidaei A Rezaei N Naghibi Iravani SS Modirian M. Patterns of obesity and overweight in the Iranian Population: findings of STEPs 2016. Front Endocrinol. (2020) 11:42. 10.3389/fendo.2020.00042 32174887 PMC7055062

[31] Djalalinia S Yoosefi M Shahin S Ghasemi E Rezaei N Ahmadi N. The levels of BMI and patterns of obesity and overweight during the COVID-19 pandemic: experience from the Iran STEPs 2021 survey. Front Endocrinol. (2022) 13:1043894. 10.3389/fendo.2022.1043894 36589796 PMC9798439

[32] Djalalinia S Azadnajafabad S Ghasemi E Yoosefi M Rezaei N Farzi Y. Protocol design for surveillance of risk factors of non–communicable diseases during the COVID-19 pandemic: an experience from Iran STEPS Survey 2021. Arch Iran Med. (2022) 25:634–46. 10.34172/aim.2022.99 PMC10685773 37543889

[33] Djalalinia S Mehdipour P Mohajer B Mohebi F Larijani B Sepanlou SG. Levels and trends of BMI, obesity, and overweight at National and Sub-national Levels in Iran from 1990 to 2016; a comprehensive pooled analysis of half a million individuals. Arch Iran Med. (2021) 24:344–53. 10.34172/aim.2021.51 34196199

[34] Hay SI Abajobir AA Abate KH Abbafati C Abbas KM Abd-Allah F. Global, regional, and national disability-adjusted life-years (DALYs) for 333 diseases and injuries and healthy life expectancy (HALE) for 195 countries and territories, 1990–2016: a systematic analysis for the Global Burden of Disease Study 2016. Lancet. (2017) 390:1260–344. 10.1016/S0140-6736(17)32130-X 28919118 PMC5605707

[35] Ebrahimi H Pishgar F Yoosefi M Moradi S Rezaei N Djalalinia S. Insulin pen use and diabetes treatment goals: a study from Iran STEPS 2016 survey. PLoS ONE. (2019) 14:e0221462. 10.1371/journal.pone.0221462 31461470 PMC6713357

[36] Mohammadi E Morasa FS Roshani S Rezaei N Azadnajafabad S Moghaddam SS. Estimating the attributable risk of vascular disorders in different ranges of fasting plasma glucose and assessing the effectiveness of anti-diabetes agents on risk reduction; questioning the current diagnostic criteria. J Diabetes Metab Disord. (2020) 19:1423–30. 10.1007/s40200-020-00663-5 33520844 PMC7843770

[37] Mohebi F Mohajer B Yoosefi M Sheidaei A Zokaei H Damerchilu B. Physical activity profile of the Iranian population: STEPS survey, 2016. BMC Public Health. (2019) 19:1–17. 10.1186/s12889-019-7592-5 31519165 PMC6743153

[38] Azadnajafabad S Ebrahimi N Mohammadi E Ghasemi E Saeedi Moghaddam S Aminorroaya A. Disparities and spatial variations of high salt intake in Iran: a subnational study of districts based on the small area estimation method. Public Health Nutr. (2021) 24:6281–91. 10.1017/S1368980021002986 34261565 PMC11148577

[39] Rezaei S Mahmoudi Z Sheidaei A Aryan Z Mahmoudi N Gohari K. Salt intake among Iranian population: the first national report on salt intake in Iran. J Hypertens. (2018) 36:2380–9. 10.1097/HJH.0000000000001836 30005027

[40] Muennig P Lubetkin E Jia H Franks P. Gender and the burden of disease attributable to obesity. Am J Public Health. (2006) 96:1662–8. 10.2105/AJPH.2005.068874 16873748 PMC1551950

