# Supplementary material for: Metabolic risk factors attributed burden in Iran at national and subnational levels, 1990 to 2019
Source: Front Public Health. 2023 Jun 1;11:1149719. doi: 10.3389/fpubh.2023.1149719 (PMC10268245; doi:10.3389/fpubh.2023.1149719)
Supplement: Supplementary Figure 1 — Age-standardized death (A) and DALY (B) rates attributable to metabolic risk factors including high SBP, high FPG, high BMI, and high LDL (per 100,000 person-year) based on rankings in Iran and its 31 provinces in 1990 and 2019, for both sexes. [file Data_Sheet_1.PDF]

Appendix 1

National and subnational burden of deaths attributable to metabolic risk factors

High SBP attributed deaths number doubled from 47 thousand (95% UI: 41–53 thousand) in 1990, to 99 thousand (89–112 thousand) in 2019. The high SBP attributed deaths rate in females decreased by 33.3% from 231.5 (197.1–265.8) in 1990 to 154.5 (130.2–176.0) in 2019. While, in males it decreased by 36.0% from 253.4 (214.8–291.8) in 1990 to 162.1 (140.5–183.4) in 2019; regarding high FPG, the number of deaths in 2019 was four folds more than that of 1990. The high FPG attributed deaths rate in males increased by 11.9% from 96.2 (71.4–133.2) in 1990 to 107.6 (79.8–148.9) in 2019, compared to females from 85.4 (63.0–120.9) in 1990 to 112.2 (81.5–156.1) in 2019 (31.3% increase).In case of high BMI the number of deaths was three folded by 2019. However, high BMI attributed deaths rate was more in females than males in both 1990 and 2019. It changed for females from 96.7 (60.6–137.2) in 1990 to 96.1 (68.5–125.6) in 2019 (-0.7%); and for males from 80.7 (43.5–125.8) in 1990 to 88.1 (57.9–121.5) in 2019 (+9.1%). In case of high LDL deaths number almost doubled from 1990 to 2019, while Comparing females and males, the high LDL attributed deaths rate per 100,000 PY was more in males than females over the study period, while the changes were more in males (-46.5% *vs.* -41.9%) (Table 1; Figure 1).

National and subnational burden of DALYs, YLLs, and YLDs attributable to metabolic risk factors

Although the high SBP attributed DALYs numbers doubled from 1.2 million (1.0–1.3) in 1990 to 2.1 million (1.9–2.3) in 2019 The DALYs rate in males was constantly higher over the study period changed from 5247.5 (4540.8 – 6009.4) to 3295.7 (2949.8–3650.7) and 4266.4 (3714.5–4858) to 2657.4 (2331.5–2961.1), Regarding high FPG the number increased around three folds from 492.0 thousand (400.0–607.0) in 1990 to 1.7 million (1.4–2.1) in 2019. Moreover, the DALYs The percent change of DALYs rate was more in females (36.7%) than males (19.2%), while the rates was constantly higher in males. In case of high BMI, the number almost doubled from 710.0 thousand (443.0–990.0) in 1990 to 1.9 million (1.4–2.5) in 2019. High BMI attributed DALYs The DALYs rate in females changed (-1.7%) from 2589.6 (1695.4–3516.8) in 1990 to 2545.9 (1886.3–3238.4) in 2019 compared to males increasing from 2248.7 (1265.1–3376.4) in 1990 to 2617.7 (1778.2–3468.9) in 2019 (+16.4%).number increased from 848 thousand (712–999) in 1990 to 1.2 million (0.9–1.4) in 2019, and DALYs The DALYs rate in females changed from 2528.9 (2017.7–3120.2) in 1990 to 1287.6 (996.7–1626.7) in 2019, and males changed from 3620.9 (2960.9–4398.9) to 1862.3 (1523.2–2233.1) (Table 1; Figure 1-B; Supplementary Figure 1).

Supplementary Figure 1-A

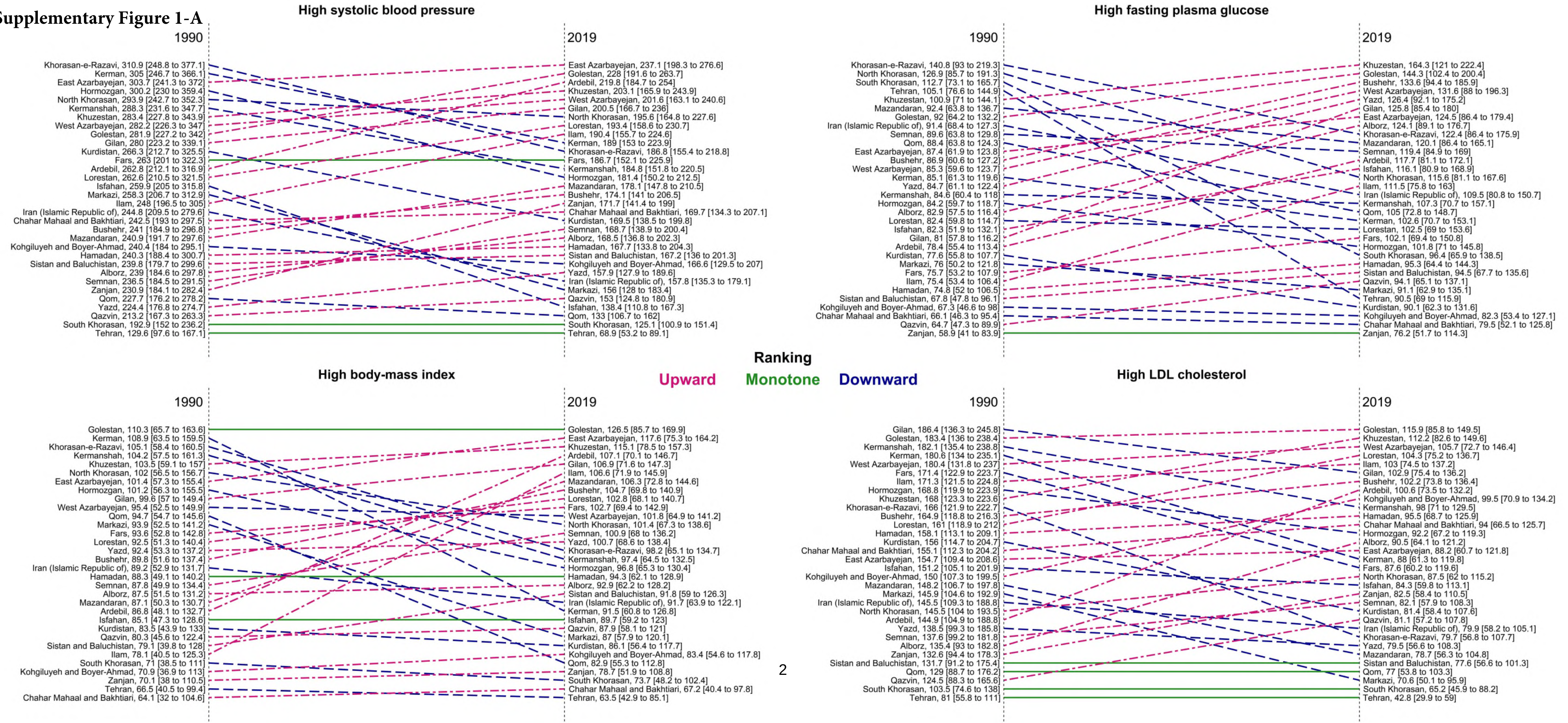

Supplementary Figure 1-B

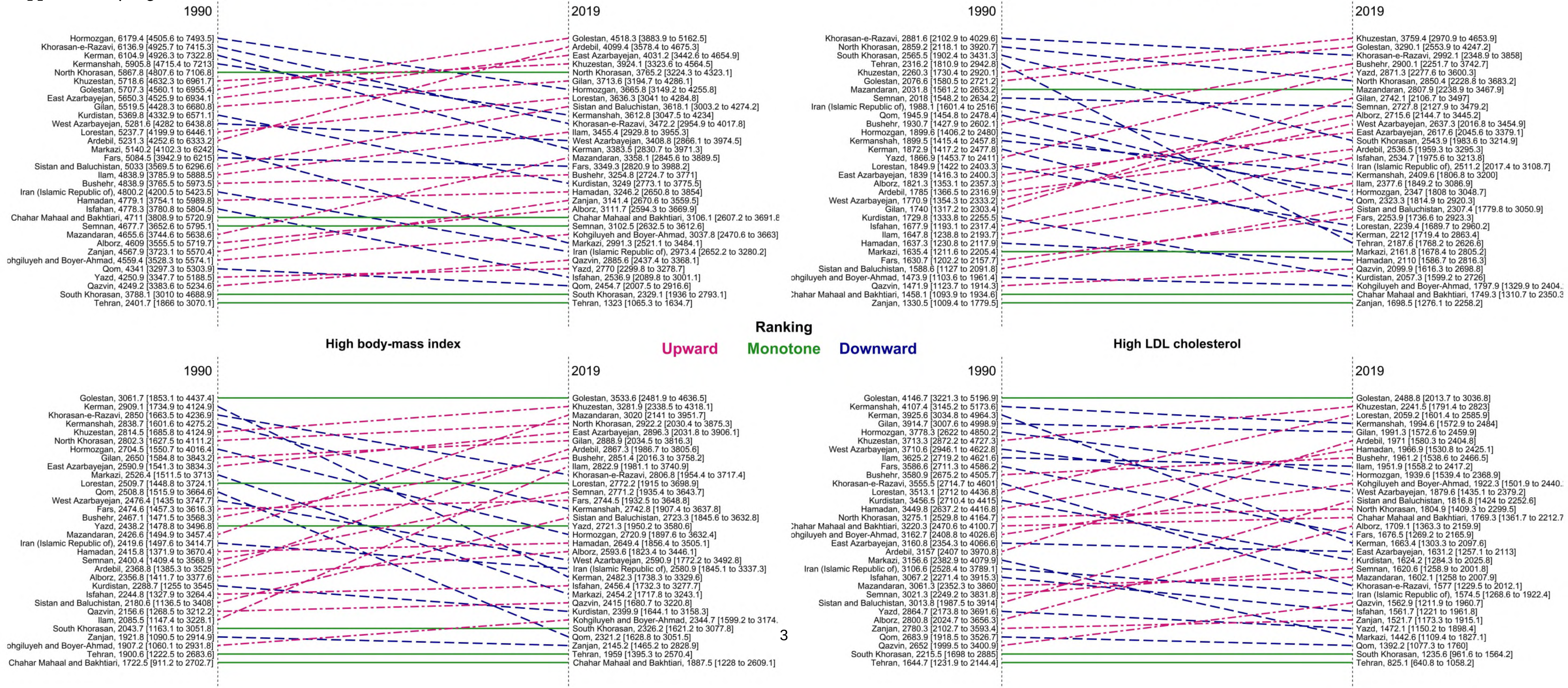

Supplementary Figure 2-A

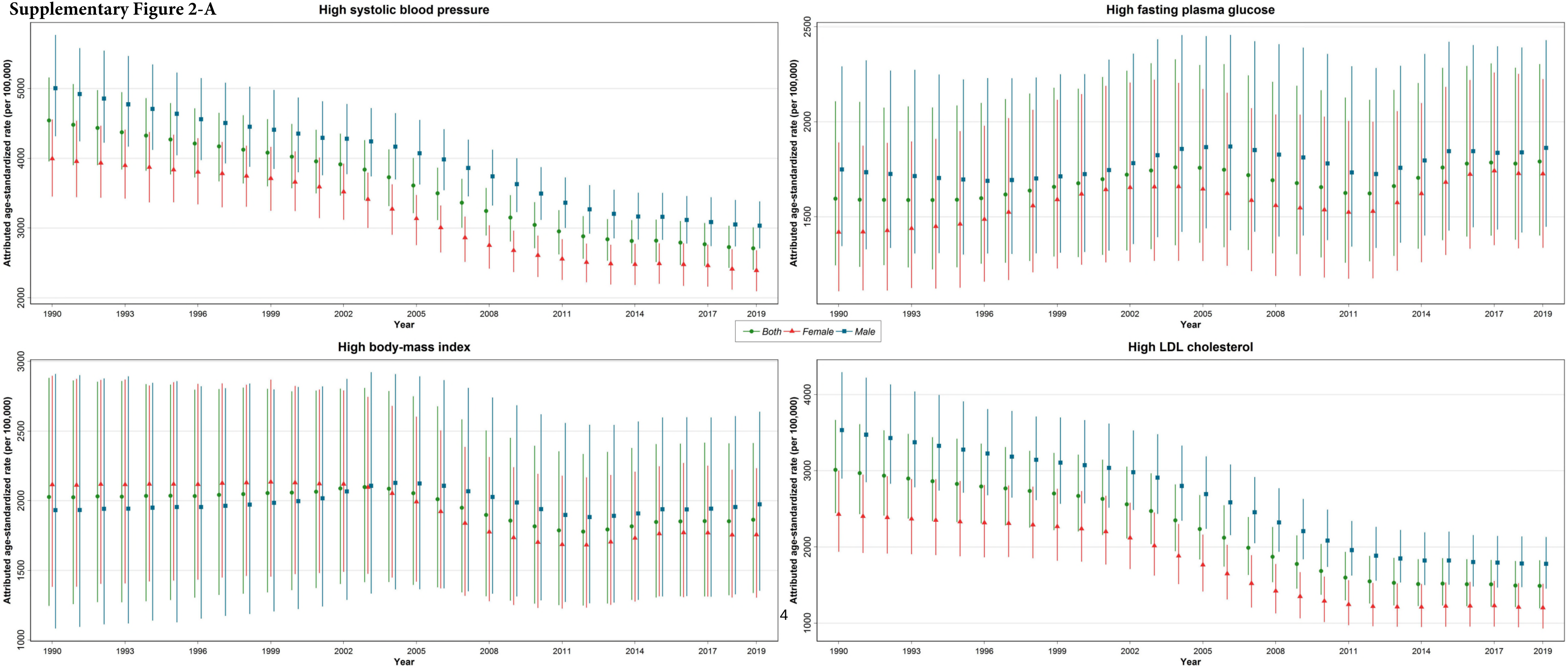

Supplementary Figure 2-B

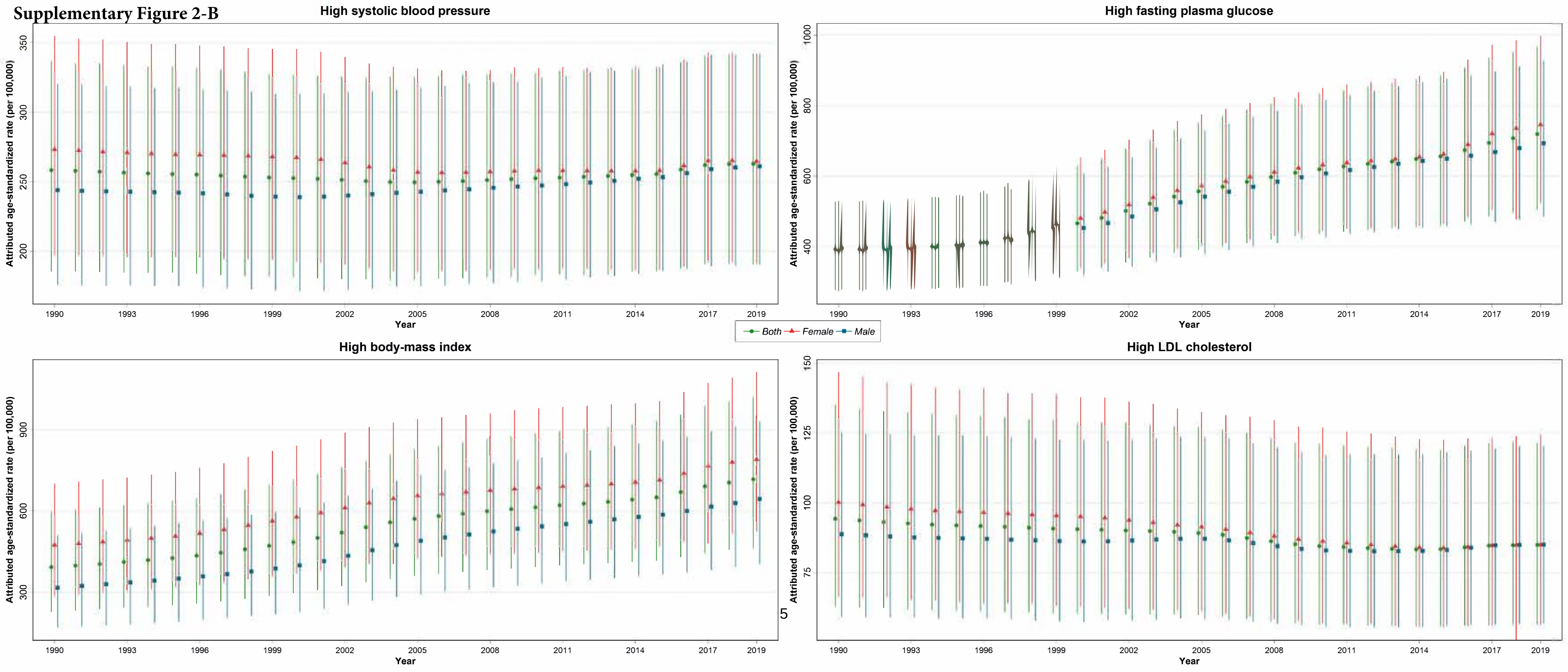

Supplementary Figure 3-A

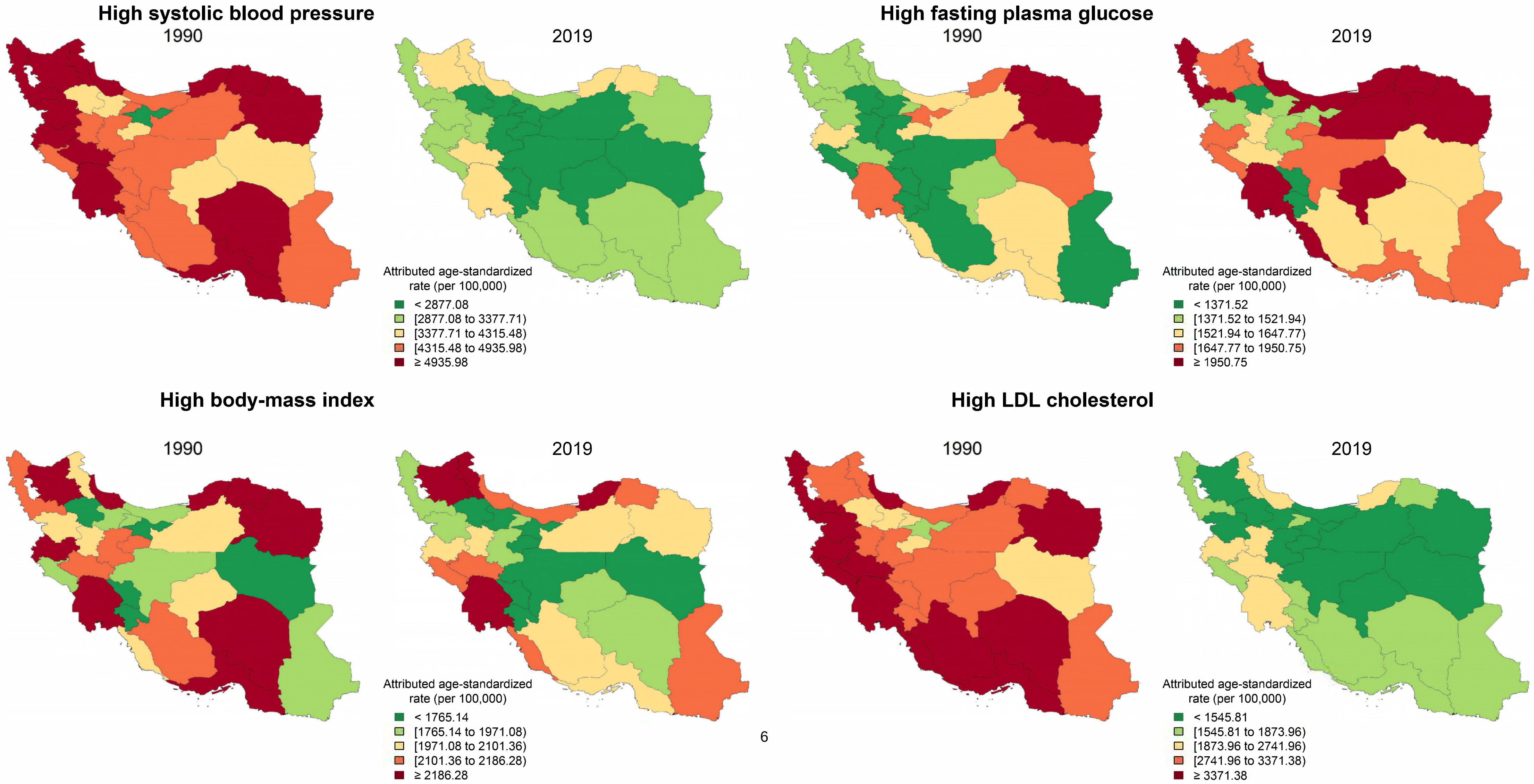

Supplementary Figure 3-B

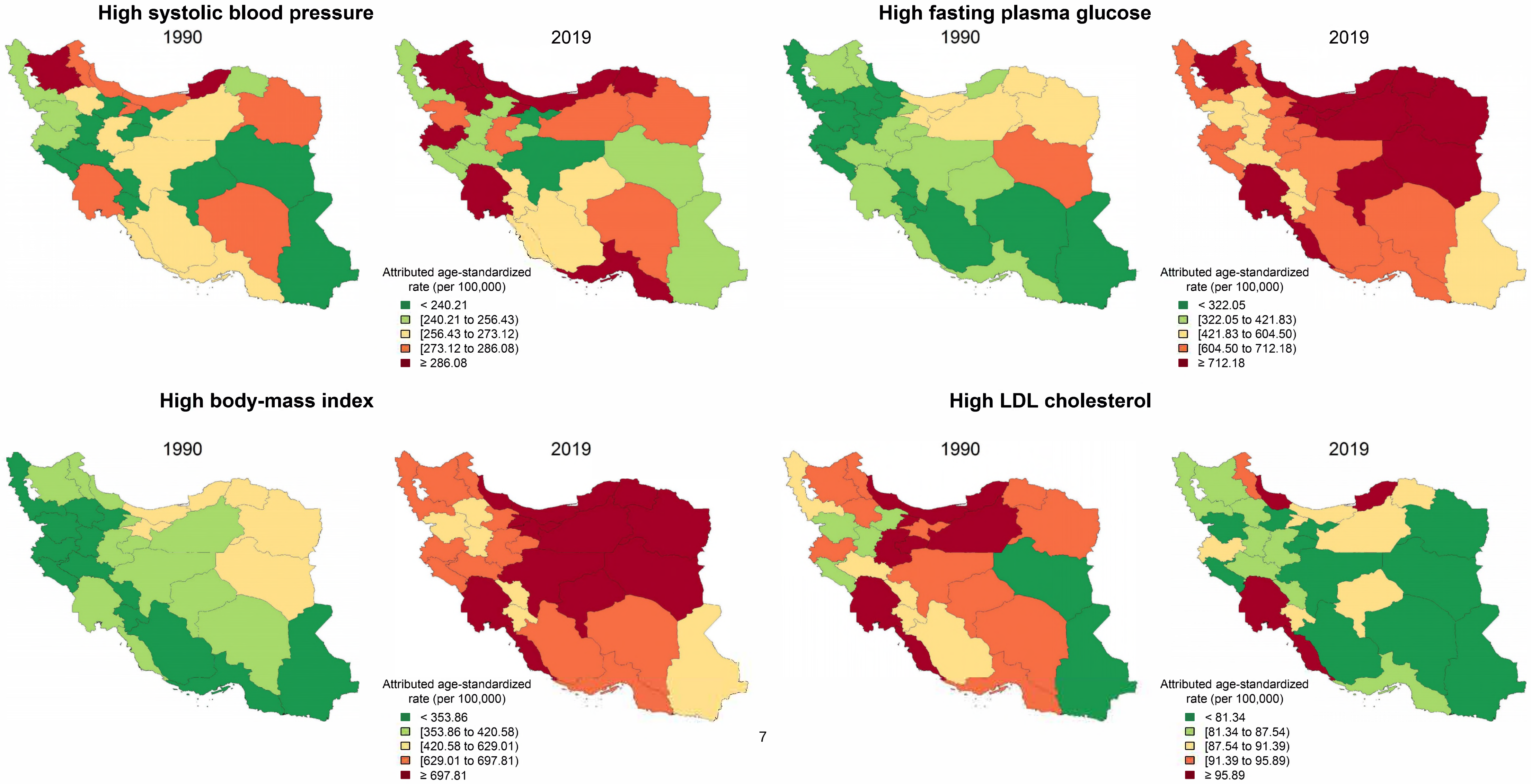

Supplementary Figure 4-A

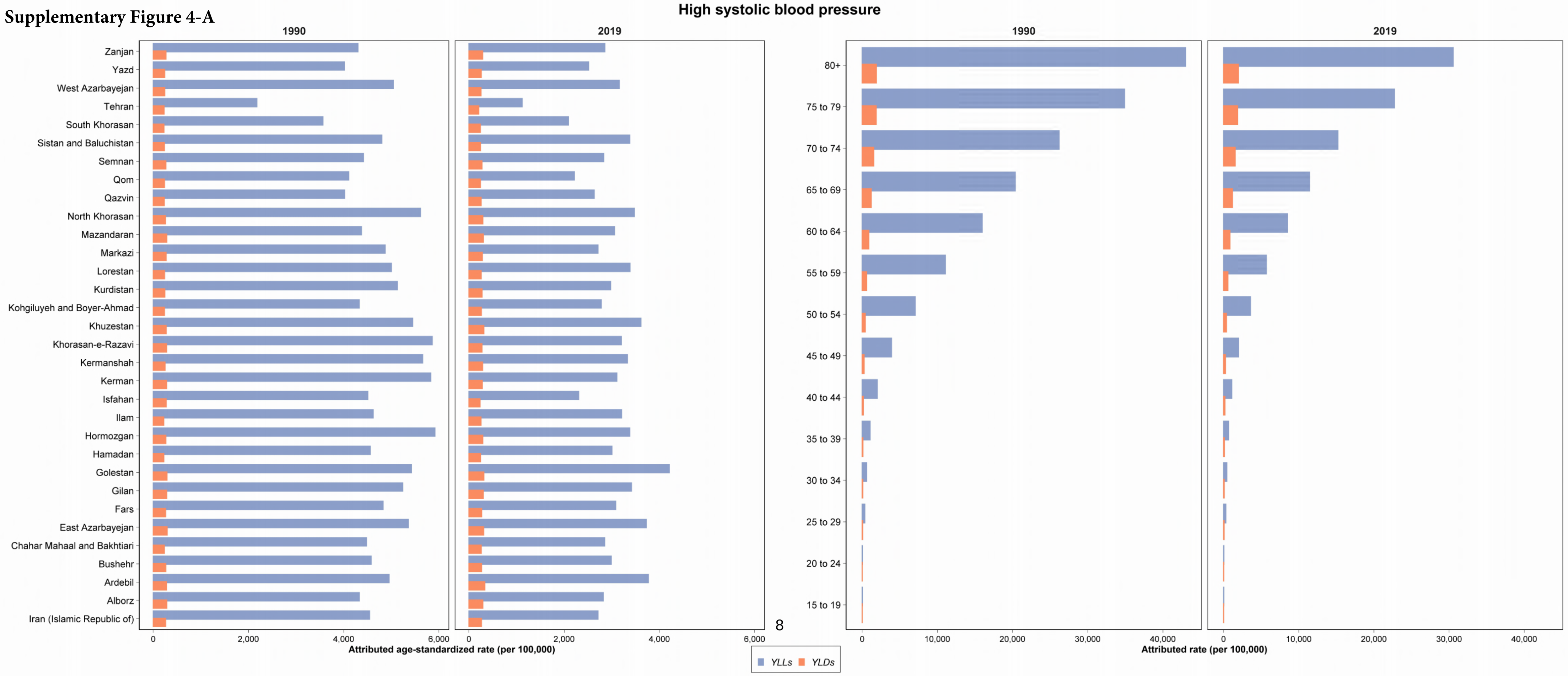

Supplementary Figure 4-B

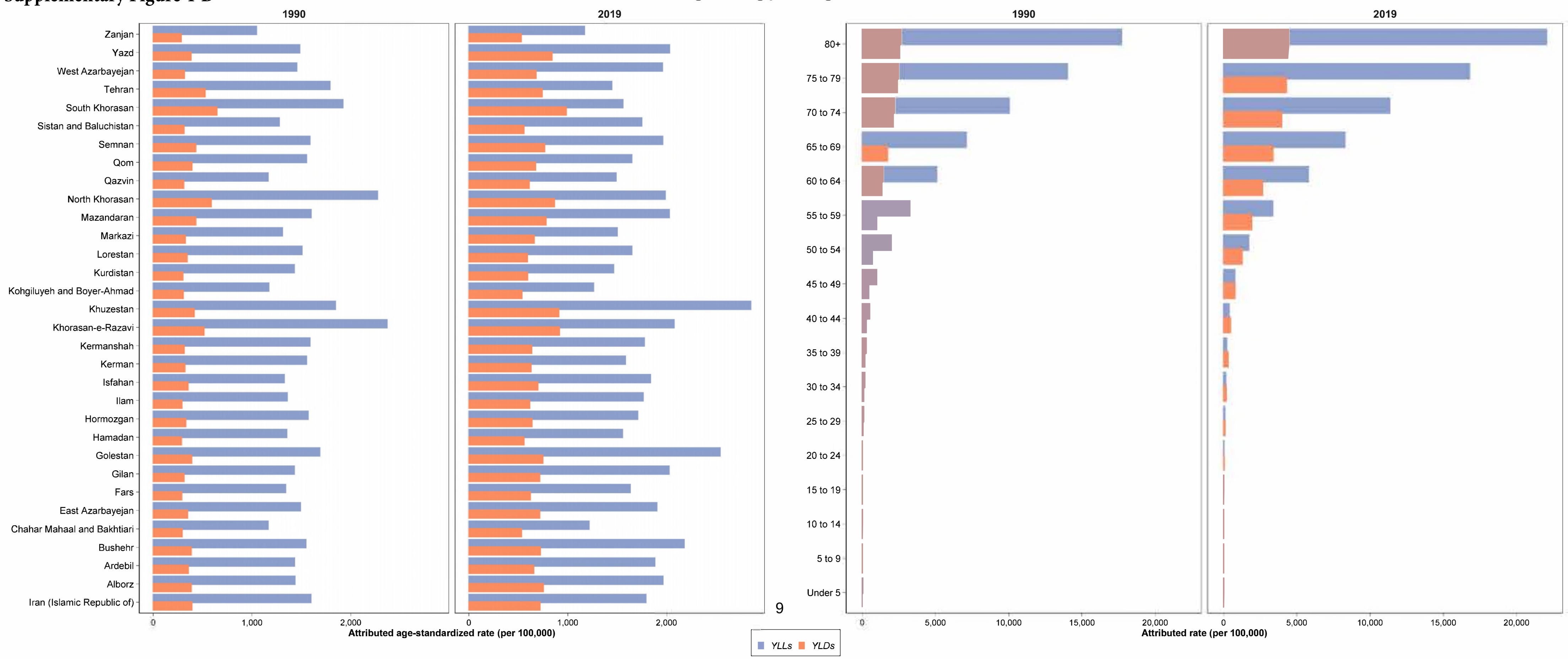

Supplementary Figure 4-C

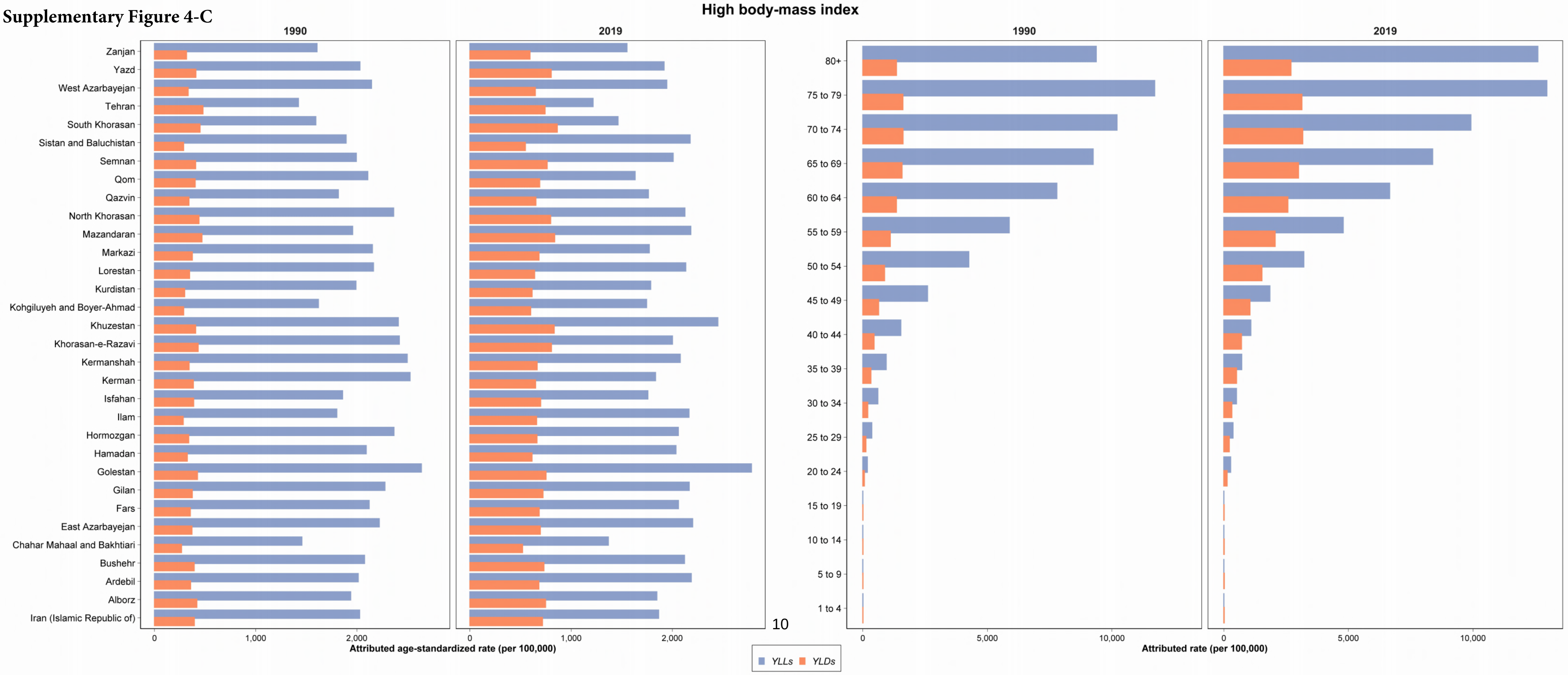

Supplementary Figure 4-D

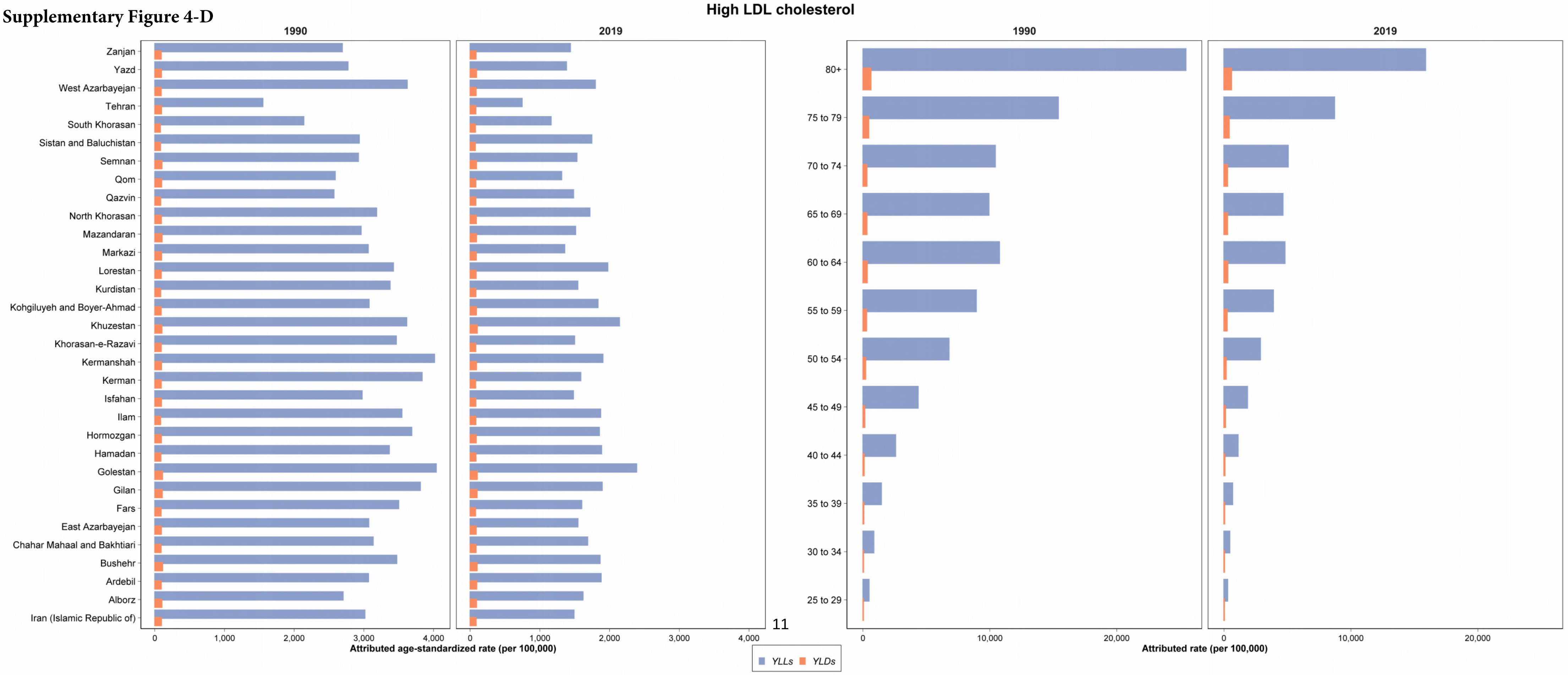

Supplementary Figure 5-A

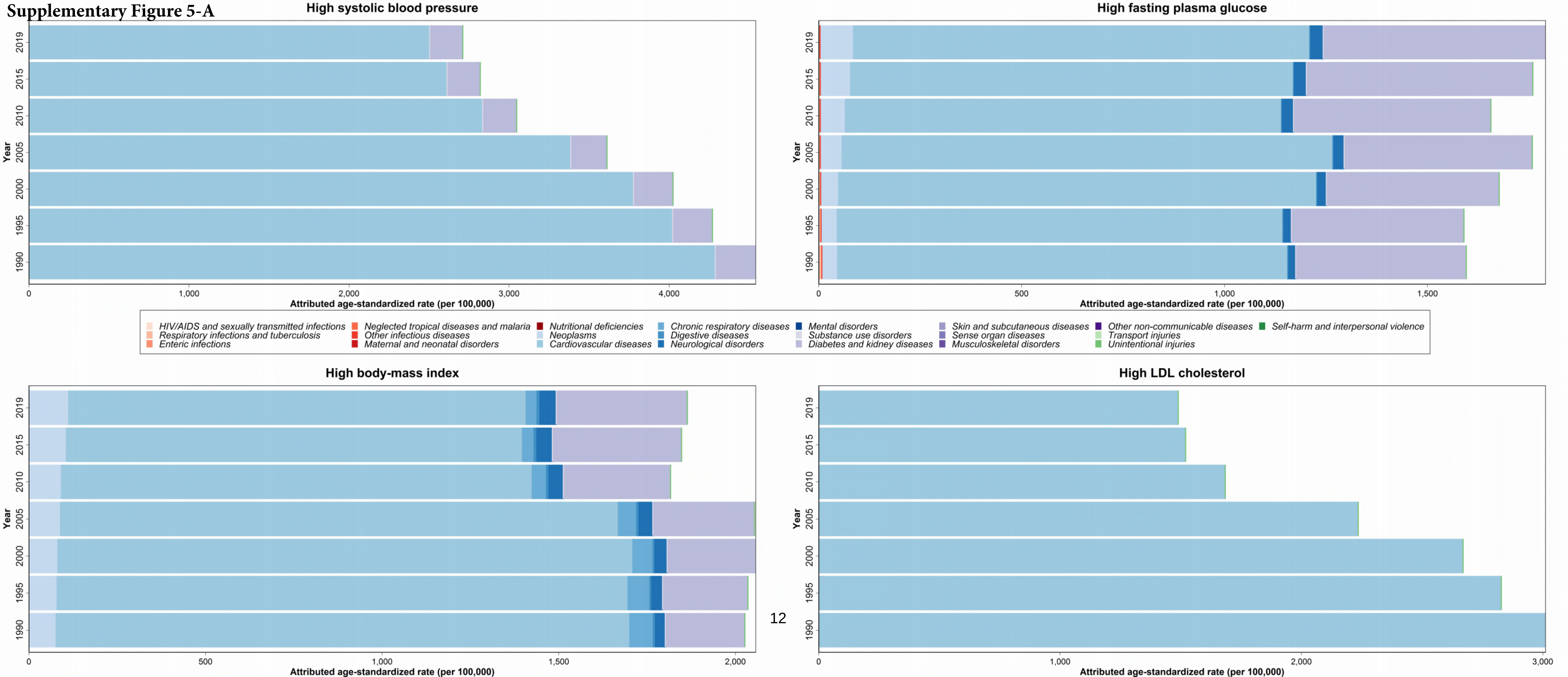

Supplementary Figure 5-B

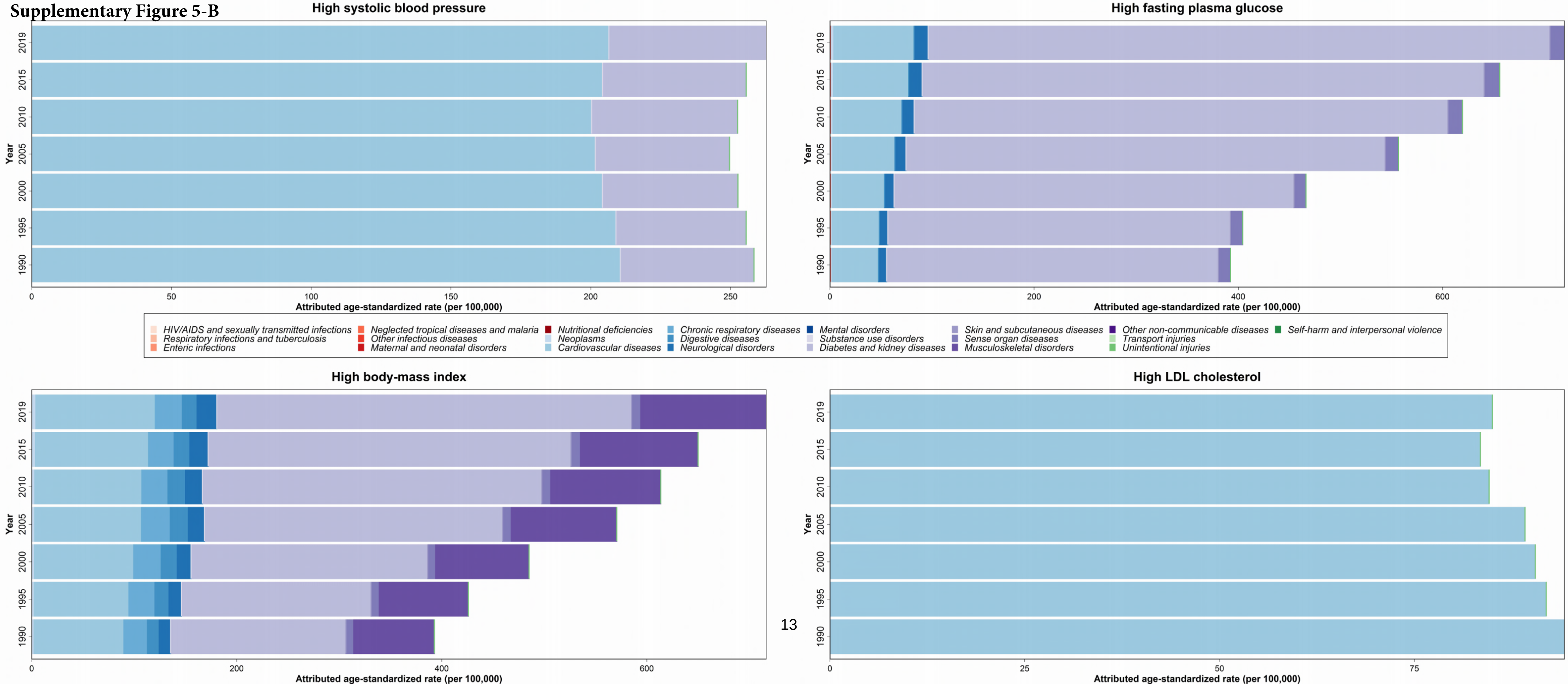

Supplementary Table 1

Suggested citation; High systolic blood pressure
World Health Organization (WHO), Ministry of Health and Medical Education (Iran), Center for Non-Communicable Diseases Control (Iran). Iran STEPS Noncommunicable Disease Risk Factors Survey 2005.
World Health Organization (WHO), Ministry of Health and Medical Education (Iran), Center for Non-Communicable Diseases Control (Iran). Iran STEPS Noncommunicable Disease Risk Factors Survey 2007.
Ministry of Health and Medical Education (Iran), World Health Organization (WHO). Iran STEPS Noncommunicable Disease Risk Factors Survey 2009.
Amini M, Afshin-Nia F, Bashardoost N, Aminorroaya A, Shahparian M, Kazemi M. Prevalence and risk factors of diabetes mellitus in the Isfahan city population (aged 40 or over) in 1993. Diabetes Res Clin Pract. 1997; 38(3): 185-90.
Ministry of Health and Medical Education (Iran), World Health Organization (WHO). Iran STEPS Noncommunicable Disease Risk Factors Survey 2008.
Fahimfar N, Khalili D, Mohebi R, Azizi F, Hadaegh F. Risk factors for ischemic stroke; results from 9 years of follow-up in a population based cohort of Iran. BMC Neurol. 2012; 12: 117.
Shirani S, Heidari K, Sabzghabaee AM, Mirmoghtadaee P, Hoseini L, Aalifar H, Fadaei H, Esnaashari H, Soltani R. The modifiable noncommunicable risk factors among an Iranian population. Southeast Asian J Trop Med Public Health. 2012; 43(5): 1227-32.
Ministry of Health and Medical Education (Iran). Iran STEPS Noncommunicable Disease Risk Factors Survey 2011.
Bushehr University of Medical Sciences. Iran Persian Gulf Healthy Heart Study (PGHHS) Phase I 2003-2004.
Ministry of Health and Medical Education (Iran), World Health Organization (WHO). Iran STEPS Noncommunicable Disease Risk Factors Survey 2006.
Esteghamati A, Meysamie A, Khalilzadeh O, Rashidi A, Haghazali M, Asgari F, Kamgar M, Gouya MM, Abbasi M. Third national Surveillance of Risk Factors of Non-Communicable Diseases (SuRFNCD-2007) in Iran: methods and results on prevalence of diabetes, hypertension, obesity, central obesity, and dyslipidemia. BMC Public Health. 2009; 167.
Azizi F, Emami H, Salehi P, Ghanbarian A, Mirmiran P, Mirbolooki M, Azizi T. Cardiovascular risk factors in the elderly: the Tehran Lipid and Glucose Study. J Cardiovasc Risk. 2003; 10(1): 65-73.
Sadeghi M, Roohafza HR, Kelishadi R. Blood Pressure and Associated Cardiovascular Risk Factors in Iran: Isfahan Healthy Heart Programme. Med J Malaysia. 2004; 59(4): 460-7.
Harati H, Hadaegh F, Saadat N, Azizi F. Population-based incidence of Type 2 diabetes and its associated risk factors: results from a six-year cohort study in Iran. BMC Public Health. 2009; 186.
Shirani S, Kelishadi R, Sarrafzadegan N, Khosravi A, Sadri G, Amani A, Heidari S, Ramezani MA. Awareness, treatment and control of hypertension, dyslipidaemia and diabetes mellitus in an Iranian population: the IHHP study. East Mediterr Health J. 2009; 15(6): 1455,Äì63.
Farzadfar F, Murray CJL, Gakidou E, Bossert T, Namdaritabar H, Alikhani S, Moradi G, Delavari A, Jamshidi H, Ezzati M. Effectiveness of diabetes and hypertension management by rural primary health-care workers (Behvarz workers) in Iran: a nationally representative observational study. Lancet. 2012; 379(9810): 47,Äì54.
Shab-Bidar S, Hosseini-Esfahani F, Mirmiran P, Hosseinpour-Niazi S, Azizi F. Metabolic syndrome profiles, obesity measures and intake of dietary fatty acids in adults: Tehran Lipid and Glucose Study. J Hum Nutr Diet. 2014; 27 Suppl 2: 98,Äì108.
Kheirandish M, Asgari S, Lotfaliany M, Bozorgmanesh M, Saadat N, Tohidi M, Azizi F, Hadaegh F. Secular trends in serum lipid levels of a Middle Eastern adult population; 10 years follow up in Tehran lipid and glucose study. Lipids Health Dis. 2014; 13: 20.
Safari M, Yazdanpanah B, Yazdanpanah B, Mobasheri A. A population-based screening of type 2 diabetes in high-risk population of Yasuj, Iran. J Health Popul Nutr. 2014; 32(4): 677,Äì86.
Ostovaneh MR, Zamani F, Sharafkhah M, Ansari-Moghaddam A, Akhavan Khaleghi N, Saeedian FS, Rohani Z, Motamed N, Maadi M, Malekzadeh R, Poustchi H. Prevalence of metabolic syndrome in Amol and Zahedan, Iran: a population based study. Arch Iran Med. 2014; 17(7): 477,Äì82.
Azimi-Nezhad M, Herbeth B, Siest G, Dad√© S, Ndiaye NC, Esmaily H, Hosseini SJ, Ghayour-Mobarhan M, Visvikis-Siest S. High prevalence of metabolic syndrome in Iran in comparison with France: what are the components that explain this?. Metab Syndr Relat Disord. 2012; 10(3): 181,Äì8.
Khosravi A, Emamian MH, Shariati M, Hashemi H, Fotouhi A. The prevalence of pre-hypertension and hypertension in an Iranian urban population. High Blood Press Cardiovasc Prev. 2014; 21(2): 127,Äì35.
Mirmiran P, Hajifaraji M, Bahadoran Z, Sarvghadi F, Azizi F. Dietary protein intake is associated with favorable cardiometabolic risk factors in adults: Tehran Lipid and Glucose Study. Nutr Res. 2012; 32(3): 169,Äì76.
Hadaegh F, Shafiee G, Ghasemi A, Sarbakhsh P, Azizi F. Impact of metabolic syndrome, diabetes and prediabetes on cardiovascular events: Tehran lipid and glucose study. Diabetes Res Clin Pract. 2010; 87(3): 342,Äì7.
Haghighatdoost F, Sarrafzadegan N, Mohammadifard N, Sajjadi F, Maghroon M, Boshtam M, Alikhasi H, Azadbakht L. Healthy eating index and cardiovascular risk factors among Iranians. J Am Coll Nutr. 2013; 32(2): 111,Äì21.

Suggested citation; High systolic blood pressure

Hadaegh F, Shafiee G, Hatami M, Azizi F. Systolic and diastolic blood pressure, mean arterial pressure and pulse pressure for prediction of cardiovascular events and mortality in a Middle Eastern population. Blood Press. 2012; 21(1): 12,Äì8.
Hosseini-Esfahani F, Mousavi Nasl Khameneh A, Mirmiran P, Ghanbarian A, Azizi F. Trends in risk factors for cardiovascular disease among Iranian adolescents: the Tehran Lipid and Glucose Study, 1999-2008. J Epidemiol. 2011; 21(5): 319,Äì28.
Ghasemi A, Zahediasl S, Syedmoradi L, Azizi F. Association between serum nitric oxide metabolites and hypertension in a general population. Int Angiol. 2011; 30(4): 380,Äì7.
Najafipour H, Nasri HR, Afshari M, Moazenzadeh M, Shokoohi M, Foroud A, Etemad K, Sadeghi B, Mirzazadeh A. Hypertension: diagnosis, control status and its predictors in general population aged between 15 and 75 years: a community-based study in southeastern Iran. Int J Public Health. 2014; 59(6): 999,Äì1009.
Ostovar A, Nabipour I, Larijani B, Heshmat R, Darabi H, Vahdat K, Ravanipour M, Mehrdad N, Raeisi A, Heidari G, Shafiee G, Haeri M, Pourbehi M, Sharifi F, Noroozi A, Tahmasebi R, Aghaei Meybodi H, Assadi M, Farrokhi S, Nemati R, Amini MR, Barekat M, Amini A, Salimipour H, Dobaradaran S, Moshtaghi D. Bushehr Elderly Health (BEH) Programme, phase I (cardiovascular system). BMJ Open. 2015; 5(12): e009597.
Khosravi-Boroujeni H, Ahmed F, Sadeghi M, Roohafza H, Talaei M, Dianatkhah M, Pourmogaddas A, Sarrafzadegan N. Does the impact of metabolic syndrome on cardiovascular events vary by using different definitions?. BMC Public Health. 2015; 15: 1313.
Ostovaneh MR, Zamani F, Ansari-Moghaddam A, Sharafkhah M, Saeedian FS, Rohani Z, Khoonsari M, Barzin G, Hemmasi G, Ajdarkosh H, Katoonizadeh A, Maadi M, Malekzadeh R, Poustchi H. Nonalcoholic Fatty Liver: The Association with Metabolic Abnormalities, Body Mass Index and Central Obesity--A Population-Based Study. Metab Syndr Relat Disord. 2015; 13(7): 304,Äì11.
International Agency for Research on Cancer (IARC), National Cancer Institute (NCI) (United States), Tehran University of Medical Sciences. Iran - Golestan Cohort Study 2004-2008 - Teheran University of Medical Sciences.
Digestive Diseases Research Institute (DDRI) (Iran), Shiraz University of Medical Sciences. Iran - Pars Cohort Study.
Aalami Harandi S, Sarrafzadegan N, Sadeghi M, Talaei M, Dianatkhah M, Oveisgharan S, Pourmoghaddas A, Salehi A, Sedighifard Z. Do Cardiometabolic Risk Factors Relative Risks Differ for the Occurrence of Ischemic Heart Disease and Stroke?. Res Cardiovasc Med. 2016; 5(1): e30619.
Yusufali AM, Khatib R, Islam S, Alhabib KF, Bahonar A, Swidan HM, Khammash U, Alshamiri MQ, Rangarajan S, Yusuf S. Prevalence, awareness, treatment and control of hypertension in four Middle East countries. J Hypertens. 2017; 35(7): 1457-1464.
Biderafsh A, Karami M, Faradmal J, Poorolajal J. Estimating the potential impact fraction of hypertension as the main risk factor of stroke: Application of the distribution shift method. J Epidemiol Glob Health. 2015; 5(3): 231-7.
Biderafsh A, Karami M, Faradmal J, Poorolajal J. Estimating the potential impact fraction of hypertension as the main risk factor of stroke: Application of the distribution shift method [Unpublished data]. J Epidemiol Glob Health. 2015; 5(3): 231-7.
Ghorbani Z, Shamshirgaran SM, Ghaffari S, Sarbakhsh P, Najafipour F, Aminisani N. Hypertension prevalence, awareness, treatment and its correlates among people 35 years and older: Result from pilot phase of the Azar cohort study. J Educ Health Promot. 2018; 7.
Mansour-Ghanaei F, Joukar F, Naghipour MR, Sepanlou SG, Poustchi H, Mojtahedi K, Balou HA, Heidarzadeh A, Malekzadeh R. The PERSIAN Guilan Cohort Study (PGCS). Arch Iran Med. 2019; 22(1): 39-45.

Supplementary Table 2

Suggested citation; High fasting plasma glucose
World Health Organization (WHO), Ministry of Health and Medical Education (Iran), Center for Non-Communicable Diseases Control (Iran). Iran STEPS Noncommunicable Disease Risk Factors Survey 2005.
World Health Organization (WHO), Ministry of Health and Medical Education (Iran), Center for Non-Communicable Diseases Control (Iran). Iran STEPS Noncommunicable Disease Risk Factors Survey 2007.
Ministry of Health and Medical Education (Iran), World Health Organization (WHO). Iran STEPS Noncommunicable Disease Risk Factors Survey 2009.
Ministry of Health and Medical Education (Iran), Statistical Centre of Iran. Iran Multiple Indicator Demographic and Health Survey 2010.
Sarraf-Zadegan N, Sayed-Tabatabaei FA, Bashardoost N, Maleki A, Totonchi M, Habibi HR, Sotodehmaram E, Tafazoli F, Karimi A. The prevalence of coronary artery disease in an urban population in Isfahan, Iran. Acta Cardiol. 1999; 54(5): 257-63.
Sarraf-Zadegan N. Risk factors for coronary artery disease in Isfahan, Iran. Eur J Public Health. 1999; 9(1): 20-26.
Shahid Beheshti University of Medical Sciences. Iran - Tehran Lipid and Glucose Study 1999-2000.
Qazvin University of Medical Sciences, Zanjan University of Medical Sciences. Iran Community-Based Pilot Study for Primary Prevention of the Major Noncommunicable Diseases in Qazvin and Abhar 2001.
Alavi A, Sanjari M, Haghdoost A, Sibbald RG. Common foot examination features of 247 Iranian patients with diabetes. Int Wound J. 2009; 6(2): 117-22.
Vatankhah N, Noudeh YJ, Khamseh ME, Baradaran HR. Screening people with type 2 diabetes at risk for foot ulceration in Iran. Diabetes Technol Ther. 2010; 12(9): 731-6.
Abdollahi A, Malekmadani MH, Mansoori MR, Bostak A, Abbaszadeh MR, Mirshahi A. Prevalence of diabetic retinopathy in patients with newly diagnosed type II diabetes mellitus. Acta Med Iran. 2006; 44(6): 415-19.
Javadi MA,¬†Katibeh M,¬†Rafati N,¬†Dehghan MH,¬†Zayeri F,¬†Yaseri M,¬†Sehat M,¬†Ahmadieh H. Prevalence of diabetic retinopathy in Tehran province: a population-based study. BMC Ophthalmol. 2009; 9(1): 12.
Amini M, Afshin-Nia F, Bashardoost N, Aminorroaya A, Shahparian M, Kazemi M. Prevalence and risk factors of diabetes mellitus in the Isfahan city population (aged 40 or over) in 1993. Diabetes Res Clin Pract. 1997; 38(3): 185-90.
Esmaeilzadeh S, Delavar MA, Zeinalzadeh M, Mir M-RA. Epidemiology of infertility: a population-based study in Babol, Iran. Women Health. 2012; 52(8): 744-54.
Ministry of Health and Medical Education (Iran), World Health Organization (WHO). Iran STEPS Noncommunicable Disease Risk Factors Survey 2008.
Janghorbani M, Amini M, Willett WC, Mehdi Gouya M, Delavari A, Alikhani S, Mahdavi A. First Nationwide Survey of Prevalence of Overweight, Underweight, and Abdominal Obesity in Iranian Adults. Obesity (Silver Spring). 2007; 15(11): 2797-808.
Ghassemi H, Harrison G, Mohammad K. An accelerated nutrition transition in Iran. Public Health Nutr. 2002; 5(1A): 149-55.
Dastgiri S, Mahdavi R, TuTunchi H, Faramarzi E. Prevalence of obesity, food choices and socio-economic status: a cross-sectional study in the north-west of Iran. Public Health Nutr. 2006; 9(8): 996-1000.
Pishdad GR. Overweight and obesity in adults aged 20-74 in southern Iran. Int J Obes Relat Metab Disord. 1996; 20(10): 963-5.
Hajian-Tilaki KO, Heidari B. Prevalence of obesity,central obesity and the associated factors in urban population aged 20-70 years, in the north of Iran: a population-based study and regression approach. Obes Rev. 2007; 8(1): 3-10.
Mohammadpour-Ahranjani B, Rashidi A, Karandish M, Eshraghian MR, Kalantari N. Prevalence of overweight and obesity in adolescent Tehrani students, 2000-2001: an epidemic health problem. Public Health Nutr. 2004; 7(5): 645-8.
Sotoudeh G, Khosravi S, Khajehnasiri F, Khalkhali HR. High prevalence of overweight and obesity in women of Islamshahr, Iran. Asia Pac J Clin Nutr. 2005; 14(2): 169-72.
Tehran University. Iran - Tehran Blood Pressure Study 1990-1991.
Fahimfar N, Khalili D, Mohebi R, Azizi F, Hadaegh F. Risk factors for ischemic stroke; results from 9 years of follow-up in a population based cohort of Iran. BMC Neurol. 2012; 12: 117.
Rajavi Z, Katibeh M, Ziaei H, Fardesmaeilpour N, Sehat M, Ahmadieh H, Javadi MA. Rapid assessment of avoidable blindness in Iran. Ophthalmology. 2011; 118(9): 1812-8.

Suggested citation; High fasting plasma glucose

Hasani N, Khosrawi S, Hashemipour M, Haghighatiyan M, Javdan Z, Taheri MH, Kelishadi R, Amini M, Barekatein R. Prevalence and related risk-factors of peripheral neuropathy in children with insulin-dependent diabetes mellitus. J Res Med Sci. 2013; 18(2): 132-6.

Manaviat MR, Rashidi M, Afkhami-Ardekani M. Four years incidence of diabetic retinopathy and effective factors on its progression in type II diabetes. Eur J Ophthalmol. 2008; 18(4): 572-7.

Pishdad GR. Low incidence of type 1 diabetes in Iran. Diabetes Care. 2005; 28(4): 927-8.

Shirani S, Heidari K, Sabzghabaee AM, Mirmoghtadaee P, Hoseini L, Aalifar H, Fadaei H, Esnaashari H, Soltani R. The modifiable noncommunicable risk factors among an Iranian population. Southeast Asian J Trop Med Public Health. 2012; 43(5): 1227-32.

Hajian-Tilaki K, Heidari B. Prevalences of overweight and obesity and their association with physical activity pattern among Iranian adolescents aged 12-17 years. Public Health Nutr. 2012; 15(12): 2246-52.

Khazaie H, Najafi F, Rezaie L, Tahmasian M, Sepehry AA, Herth FJF. Prevalence of symptoms and risk of obstructive sleep apnea syndrome in the general population. Arch Iran Med. 2011; 14(5): 335-8.

Kelishadi R, Ardalan G, Gheiratmand R, Majdzadeh R, Hosseini M, Gouya MM, Razaghi EM, Delavari A, Motaghian M, Barekati H, Mahmoud-Arabi MS, Lock K, Caspian Study Group. Thinness, overweight and obesity in a national sample of Iranian children and adolescents: CASPIAN Study. Child Care Health Dev. 2008; 34(1): 44-54.

Ministry of Health and Medical Education (Iran). Iran STEPS Noncommunicable Disease Risk Factors Survey 2011.

Bushehr University of Medical Sciences. Iran Persian Gulf Healthy Heart Study (PGHHS) Phase I 2003-2004.

Gakidou E,¬†Mallinger L,¬†Abbott-Klafter J,¬†Guerrero R,¬†Villalpando S,¬†Ridaura RL,¬†Aekplakorn W,¬†Naghavi M,¬†Lim S,¬†Lozano R,¬†Murray CJ. Management of diabetes and associated cardiovascular risk factors in seven countries: a comparison of data from national health examination surveys. Bull World Health Organ. 2011; 89(3): 172-83.

Ministry of Health and Medical Education (Iran), World Health Organization (WHO). Iran STEPS Noncommunicable Disease Risk Factors Survey 2006.

Dorosty AR, Siassi F, Reilly JJ. Obesity in Iranian children. Arch Dis Child. 2002; 87(5): 388-391.

Bakhshi E, Eshraghian MR, Mohammad K, Foroushani AR, Zeraati H, Fotouhi A, Siassi F, Seifi B. Sociodemographic and smoking associated with obesity in adult women in Iran: results from the National Health Survey. J Public Health (Oxf). 2008; 30(4): 429-35.

Kelishadi R, Gharipour M, Sadri GH, Tavasoli AA, Amani A. Cardiovascular disease risk factors, metabolic syndrome and obesity in an Iranian population. East Mediterr Health J. 2008; 14(5): 1070-9.

Fazizi F, Esmaillzadeh A, Mirmiran FP. Obesity and cardiovascular disease risk factors in Tehran adults: a population-based study. East Mediterr Health J. 2004; 10(6): 887-97.

Esmaillzadeh A, Mirmiran P, Azizi F. Comparative evaluation of anthropometric measures to predict cardiovascular risk factors in Tehranian adult women. Public Health Nutr. 2006; 9(1): 61-9.

Nabipour I, Amiri M, Imami SR, Jahfari SM, Nosrati A, Iranpour D, Soltanian AR. Unhealthy lifestyles and ischaemic electrocardiographic abnormalities: the Persian Gulf Healthy Heart Study. East Mediterr Health J. 2008; 14(4): 858-68.

Maddah M. Overweight and obesity among Iranian female adolescents in Rasht: more overweight in the lower social group. Public Health Nutr. 2007; 10(5): 450-3.

Hajian-Tilaki KO, Heidari B. Prevalence of obesity, central obesity and the associated factors in urban population aged 20-70 years, in the north of Iran: a population-based study and regression approach. Obes Rev. 2007; 8(1): 3-10.

Janghorbani M, Amini M, Rezvanian H, Gouya M-M, Delavari A, Alikhani S, Mahdavi A. Association of body mass index and abdominal obesity with marital status in adults. Arch Iran Med. 2008; 11(3): 274-81.

Azadbakht L, Esmaillzadeh A. Dietary and non-dietary determinants of central adiposity among Tehrani women. Public Health Nutr. 2008; 11(5): 528-34.

Maddah M, Nikooyeh B. Factors associated with overweight in children in Rasht, Iran: gender, maternal education, skipping breakfast and parental obesity. Public Health Nutr. 2010; 13(2): 196-200.

Maddah M, Nikooyeh B. Obesity among Iranian adolescent girls: location of residence and parental obesity. J Health Popul Nutr. 2010; 28(1): 61-6.

Esteghamati A, Meysamie A, Khalilzadeh O, Rashidi A, Haghazali M, Asgari F, Kamgar M, Gouya MM, Abbasi M. Third national Surveillance of Risk Factors of Non-Communicable Diseases (SuRFNCD-2007) in Iran: methods and results on prevalence of diabetes, hypertension, obesity, central obesity, and dyslipidemia. BMC Public Health. 2009; 167.

Soori H. Pattern of dietary behaviour and obesity in Ahwaz, Islamic Republic of Iran. East Mediterr Health J. 2001; 7(1-2): 163-70.

Suggested citation; High fasting plasma glucose

Fakhrzadeh H, Ghaderpanahi M, Sharifi F, Badamchizade Z, Mirarefin M, Larijani B. Increased risk of chronic kidney disease in elderly with metabolic syndrome and high levels of C-reactive protein: Kahrizak Elderly Study. Kidney Blood Press Res. 2009; 32(6): 457-63.
Azizi F, Azadbakht L, Mirmiran P. Trends in overweight, obesity and central fat accumulation among Tehranian adults between 1998-1999 and 2001-2002: Tehran lipid and glucose study. Ann Nutr Metab. 2005; 49(1): 3-8.
Azizi F, Rahmani M, Emami H, Mirmiran P, Hajipour R, Madjid M, Ghanbili J, Ghanbarian A, Mehrabi Y, Saadat N, Salehi P, Mortazavi N, Heydarian P, Sarbazi N, Allahverdian S, Saadati N, Ainy E, Moeini S. Cardiovascular risk factors in an Iranian urban population: Tehran lipid and glucose study (phase 1). Soz Praventivmed. 2002; 47(6): 408-26.
Sadeghi M, Roohafza H, Shirani S, Poormoghadas M, Kelishadi R, Baghaii A, Sarraf-Zadegan N. Diabetes and associated cardiovascular risk factors in Iran: the Isfahan Healthy Heart Programme. Ann Acad Med Singapore. 2007; 36(3): 175-80.
Hamilton Health Sciences, McMaster University (Canada), Population Health Research Institute (PHRI). Iran Prospective Urban and Rural Epidemiological Study.
Kiani J, Moghimbeigi A, Azizkhani H, Kosarifard S. The prevalence and associated risk factors of peripheral diabetic neuropathy in Hamedan, Iran. Arch Iran Med. 2013; 16(1): 17,Äì9.
Tabatabaei-Malazy O, Mohajeri-Tehrani M, Madani S, Heshmat R, Larijani B. The prevalence of diabetic peripheral neuropathy and related factors. Iran J Public Health. 2011; 40(3): 55,Äì62.
Janghorbani M, Rezvanian H, Kachooei A, Ghorbani A, Chitsaz A, Izadi F, Amini M. Peripheral neuropathy in type 2 diabetes mellitus in Isfahan, Iran: prevalence and risk factors. Acta Neurol Scand. 2006; 114(6): 384,Äì91.
Golozar A, Khademi H, Kamangar F, Poutschi H, Islami F, Abnet CC, Freedman ND, Taylor PR, Pharoah P, Boffetta P, Brennan PJ, Dawsey SM, Malekzadeh R, Etemadi A. Diabetes mellitus and its correlates in an Iranian adult population. PLoS One. 2011; 6(10): e26725.
Janghorbani M, Amini M. Normal fasting plasma glucose and risk of prediabetes and type 2 diabetes: the Isfahan Diabetes Prevention Study. Rev Diabet Stud. 2011; 8(4): 490-8.
Derakhshan A, Sardarinia M, Khalili D, Momenan AA, Azizi F, Hadaegh F. Sex specific incidence rates of type 2 diabetes and its risk factors over 9 years of follow-up: Tehran Lipid and Glucose Study. PLoS One. 2014; 9(7): e102563.
Azimi-Nezhad M, Ghayour-Mobarhan M, Parizadeh MR, Safarian M, Esmaeili H, Parizadeh SM, Khodaee G, Hosseini J, Abasalti Z, Hassankhani B, Ferns G. Prevalence of type 2 diabetes mellitus in Iran and its relationship with gender, urbanisation, education, marital status and occupation. Singapore Med J. 2008; 49(7): 571-6.
Chahkandi T, Taheri F, Kazemi T, Bijari B. The Prevalence of Diabetes and Prediabetes Among Elementary School Children in Birjand. Iran J Pediatr. 2015; 25(1): e183.
Janghorbani M, Amini M. Comparison of fasting glucose with post-load glucose values and glycated hemoglobin for prediction of type 2 diabetes: the Isfahan diabetes prevention study. Rev Diabet Stud. 2009; 6(2): 117-23.
Harati H, Hadaegh F, Saadat N, Azizi F. Population-based incidence of Type 2 diabetes and its associated risk factors: results from a six-year cohort study in Iran. BMC Public Health. 2009; 186.
Janghorbani M, Amini M, Ghanbari H, Safaiee H. Incidence of and risk factors for diabetic retinopathy in Isfahan, Iran. Ophthalmic Epidemiol. 2003; 10(2): 81-95.
Dehghan MH, Katibeh M, Ahmadieh H, Nourinia R, Yaseri M. Prevalence and risk factors for diabetic retinopathy in the 40 to 80 year-old population in Yazd, Iran: the Yazd Eye Study. J Diabetes. 2015; 7(1): 139-41.
Shirani S, Kelishadi R, Sarrafzadegan N, Khosravi A, Sadri G, Amani A, Heidari S, Ramezani MA. Awareness, treatment and control of hypertension, dyslipidaemia and diabetes mellitus in an Iranian population: the IHHP study. East Mediterr Health J. 2009; 15(6): 1455,Äì63.
Farzadfar F, Murray CJL, Gakidou E, Bossert T, Namdaritabar H, Alikhani S, Moradi G, Delavari A, Jamshidi H, Ezzati M. Effectiveness of diabetes and hypertension management by rural primary health-care workers (Behvarz workers) in Iran: a nationally representative observational study. Lancet. 2012; 379(9810): 47,Äì54.
Shab-Bidar S, Hosseini-Esfahani F, Mirmiran P, Hosseinpour-Niazi S, Azizi F. Metabolic syndrome profiles, obesity measures and intake of dietary fatty acids in adults: Tehran Lipid and Glucose Study. J Hum Nutr Diet. 2014; 27 Suppl 2: 98,Äì108.
Lotfi MH, Saadati H, Afzali M. Prevalence of diabetes in people aged ,â• 30 years: the results of screen-ing program of Yazd Province, Iran, in 2012. J Res Health Sci. 2014; 14(1): 87,Äì91.
Kheirandish M, Asgari S, Lotfaliany M, Bozorgmanesh M, Saadat N, Tohidi M, Azizi F, Hadaegh F. Secular trends in serum lipid levels of a Middle Eastern adult population; 10 years follow up in Tehran lipid and glucose study. Lipids Health Dis. 2014; 13: 20.
Safari M, Yazdanpanah B, Yazdanpanah B, Mobasheri A. A population-based screening of type 2 diabetes in high-risk population of Yasuj, Iran. J Health Popul Nutr. 2014; 32(4): 677,Äì86.
Ostovaneh MR, Zamani F, Sharafkhah M, Ansari-Moghaddam A, Akhavan Khaleghi N, Saeedian FS, Rohani Z, Motamed N, Maadi M, Malekzadeh R, Poustchi H. Prevalence of metabolic syndrome in Amol and Zahedan, Iran: a population based study. Arch Iran Med. 2014; 17(7): 477,Äì82.
Azimi-Nezhad M, Herbeth B, Siest G, Dad√© S, Ndiaye NC, Esmaily H, Hosseini SJ, Ghayour-Mobarhan M, Visvikis-Siest S. High prevalence of metabolic syndrome in Iran in comparison with France: what are the components that explain this?. Metab Syndr Relat Disord. 2012; 10(3): 181,Äì8.

Suggested citation; High fasting plasma glucose
Mirmiran P, Hajifaraji M, Bahadoran Z, Sarvghadi F, Azizi F. Dietary protein intake is associated with favorable cardiometabolic risk factors in adults: Tehran Lipid and Glucose Study. Nutr Res. 2012; 32(3): 169,Äì76.
Hadaegh F, Shafiee G, Ghasemi A, Sarbakhsh P, Azizi F. Impact of metabolic syndrome, diabetes and prediabetes on cardiovascular events: Tehran lipid and glucose study. Diabetes Res Clin Pract. 2010; 87(3): 342,Äì7.
Haghighatdoost F, Sarrafzadegan N, Mohammadifard N, Sajjadi F, Maghroon M, Boshtam M, Alikhasi H, Azadbakht L. Healthy eating index and cardiovascular risk factors among Iranians. J Am Coll Nutr. 2013; 32(2): 111,Äì21.
Janghorbani M, Amini M. Associations of hip circumference and height with incidence of type 2 diabetes: the Isfahan diabetes prevention study. Acta Diabetol. 2012; 49 Suppl 1: S107,Äì114.
Hadaegh F, Shafiee G, Hatami M, Azizi F. Systolic and diastolic blood pressure, mean arterial pressure and pulse pressure for prediction of cardiovascular events and mortality in a Middle Eastern population. Blood Press. 2012; 21(1): 12,Äì8.
Ghasemi A, Zahediasl S, Syedmoradi L, Azizi F. Association between serum nitric oxide metabolites and hypertension in a general population. Int Angiol. 2011; 30(4): 380,Äì7.
Najafian J, Mohamadifard N, Siadat ZD, Sadri G, Rahmati MR. Association between sleep duration and diabetes mellitus: Isfahan Healthy Heart Program. Niger J Clin Pract. 2013; 16(1): 59,Äì62.
Ghorbani A, Ziaee A, Esmailzadehha N, Javadi H. Association between health-related quality of life and impaired glucose metabolism in Iran: the Qazvin Metabolic Diseases Study. Diabet Med. 2014; 31(6): 754,Äì8.
Massarrat M-S, Tahaghoghi-Mehrizi S. Iranian National Health Survey: A Brief report. Arch Iran Med. 2002; 5(2): 73,Äì9.
Ahadi Z, Qorbani M, Kelishadi R, Ardalan G, Motlagh ME, Asayesh H, Zeynali M, Chinian M, Larijani B, Shafiee G, Heshmat R. Association between breakfast intake with anthropometric measurements, blood pressure and food consumption behaviors among Iranian children and adolescents: the CASPIAN-IV study. Public Health. 2015; 129(6): 740,Äì7.
Ostovar A, Nabipour I, Larijani B, Heshmat R, Darabi H, Vahdat K, Ravanipour M, Mehrdad N, Raeisi A, Heidari G, Shafiee G, Haeri M, Pourbehi M, Sharifi F, Noroozi A, Tahmasebi R, Aghaei Meybodi H, Assadi M, Farrokhi S, Nemati R, Amini MR, Barekat M, Amini A, Salimipour H, Dobaradaran S, Moshtaghi D. Bushehr Elderly Health (BEH) Programme, phase I (cardiovascular system). BMJ Open. 2015; 5(12): e009597.
Khosravi-Boroujeni H, Ahmed F, Sadeghi M, Roohafza H, Talaei M, Dianatkhah M, Pourmogaddas A, Sarrafzadegan N. Does the impact of metabolic syndrome on cardiovascular events vary by using different definitions?. BMC Public Health. 2015; 15: 1313.
Ebrahimi H, Emamian MH, Hashemi H, Fotouhi A. High Incidence of Diabetes Mellitus Among a Middle-Aged Population in Iran: A Longitudinal Study. Can J Diabetes. 2016; 40(6): 570,Äì5.
International Agency for Research on Cancer (IARC), National Cancer Institute (NCI) (United States), Tehran University of Medical Sciences. Iran - Golestan Cohort Study 2004-2008 - Teheran University of Medical Sciences.
Digestive Diseases Research Institute (DDRI) (Iran), Shiraz University of Medical Sciences. Iran - Pars Cohort Study.
Litwak L, Goh SY, Hussein Z, Malek R, Prusty V, Khamseh ME. Prevalence of diabetes complications in people in type 2 diabetes mellitus and its association with baseline characteristics in the multinational A1chieve study . Diabetol Metab Syndr. 2013; 5(1): 57.
Noshad S, Abbasi M, Etemad K, Meysamie A, Afarideh M, Khajeh E, Asgari F, Mousavizadeh M, Rafei A, Neishaboury M, Ghajar A, Nakhjavani M, Koohpayehzadeh J, Esteghamati A. Prevalence of metabolic syndrome in Iran: A 2011 update. J Diabetes. 2017; 9(5): 518-525.
Golozar A, Khalili D, Etemadi A, Poustchi H, Fazeltabar A, Hosseini F, Kamangar F, Khoshnia M, Islami F, Hadaegh F, Brennan P, Boffetta P, Abnet CC, Dawsey SM, Azizi F, Malekzadeh R, Danaei G. White rice intake and incidence of type-2 diabetes: analysis of two prospective cohort studies from Iran. BMC Public Health. 2017; 17(1): 133.
Hosseinpanah F, Rambod M, Azizi F. Population attributable risk for diabetes associated with excess weight in Tehranian adults: a population-based cohort study. BMC Public Health. 2007; 7: 328.
Mansour-Ghanaei F, Joukar F, Naghipour MR, Sepanlou SG, Poustchi H, Mojtahedi K, Balou HA, Heidarzadeh A, Malekzadeh R. The PERSIAN Guilan Cohort Study (PGCS). Arch Iran Med. 2019; 22(1): 39-45.
Janghorbani M, Amini M. Cataract in type 2 diabetes mellitus in Isfahan, Iran: incidence and risk factors. Ophthalmic Epidemiol. 2004; 11(5): 347-58.

Supplementary Table 3

Suggested citation; High body-mass index
World Health Organization (WHO), Ministry of Health and Medical Education (Iran), Center for Non-Communicable Diseases Control (Iran). Iran STEPS Noncommunicable Disease Risk Factors Survey 2005.
World Health Organization (WHO), Ministry of Health and Medical Education (Iran), Center for Non-Communicable Diseases Control (Iran). Iran STEPS Noncommunicable Disease Risk Factors Survey 2007.
Ministry of Health and Medical Education (Iran), World Health Organization (WHO). Iran STEPS Noncommunicable Disease Risk Factors Survey 2009.
Ministry of Health and Medical Education (Iran), World Health Organization (WHO). Iran WHO Multi-country Survey Study on Health and Health System Responsiveness 2000-2001. Geneva, Switzerland: World Health Organization (WHO).
Sarraf-Zadegan N. Risk factors for coronary artery disease in Isfahan, Iran. Eur J Public Health. 1999; 9(1): 20-26.
Shahid Beheshti University of Medical Sciences. Iran - Tehran Lipid and Glucose Study 1999-2000.
Qazvin University of Medical Sciences, Zanjan University of Medical Sciences. Iran Community-Based Pilot Study for Primary Prevention of the Major Noncommunicable Diseases in Qazvin and Abhar 2001.
Esmaeilzadeh S, Delavar MA, Zeinalzadeh M, Mir M-RA. Epidemiology of infertility: a population-based study in Babol, Iran. Women Health. 2012; 52(8): 744-54.
Ministry of Health and Medical Education (Iran), World Health Organization (WHO). Iran STEPS Noncommunicable Disease Risk Factors Survey 2008.
Janghorbani M, Amini M, Willett WC, Mehdi Gouya M, Delavari A, Alikhani S, Mahdavi A. First Nationwide Survey of Prevalence of Overweight, Underweight, and Abdominal Obesity in Iranian Adults. Obesity (Silver Spring). 2007; 15(11): 2797-808.
Ghassemi H, Harrison G, Mohammad K. An accelerated nutrition transition in Iran. Public Health Nutr. 2002; 5(1A): 149-55.
Dastgiri S, Mahdavi R, TuTunchi H, Faramarzi E. Prevalence of obesity, food choices and socio-economic status: a cross-sectional study in the north-west of Iran. Public Health Nutr. 2006; 9(8): 996-1000.
Pishdad GR. Overweight and obesity in adults aged 20-74 in southern Iran. Int J Obes Relat Metab Disord. 1996; 20(10): 963-5.
Hajian-Tilaki KO, Heidari B. Prevalence of obesity,central obesity and the associated factors in urban population aged 20-70 years, in the north of Iran: a population-based study and regression approach. Obes Rev. 2007; 8(1): 3-10.
Mohammadpour-Ahranjani B, Rashidi A, Karandish M, Eshraghian MR, Kalantari N. Prevalence of overweight and obesity in adolescent Tehrani students, 2000-2001: an epidemic health problem. Public Health Nutr. 2004; 7(5): 645-8.
Sotoudeh G, Khosravi S, Khajehnasiri F, Khalkhali HR. High prevalence of overweight and obesity in women of Islamshahr, Iran. Asia Pac J Clin Nutr. 2005; 14(2): 169-72.
Tehran University. Iran - Tehran Blood Pressure Study 1990-1991.
Kelishadi R, Hashemipour M, Sarraf-Zadegan N, Amiri M. Trend of atherosclerosis risk factors in children of Isfahan. Asian Cardiovasc Thorac Ann. 2001; 9(1): 36-40.
Shirani S, Heidari K, Sabzghabaee AM, Mirmoghtadaee P, Hoseini L, Aalifar H, Fadaei H, Esnaashari H, Soltani R. The modifiable noncommunicable risk factors among an Iranian population. Southeast Asian J Trop Med Public Health. 2012; 43(5): 1227-32.
Hajian-Tilaki K, Heidari B. Prevalences of overweight and obesity and their association with physical activity pattern among Iranian adolescents aged 12-17 years. Public Health Nutr. 2012; 15(12): 2246-52.
Khazaie H, Najafi F, Rezaie L, Tahmasian M, Sepehry AA, Herth FJF. Prevalence of symptoms and risk of obstructive sleep apnea syndrome in the general population. Arch Iran Med. 2011; 14(5): 335-8.
Kelishadi R, Ardalan G, Gheiratmand R, Majdzadeh R, Hosseini M, Gouya MM, Razaghi EM, Delavari A, Motaghian M, Barekati H, Mahmoud-Arabi MS, Lock K, Caspian Study Group. Thinness, overweight and obesity in a national sample of Iranian children and adolescents: CASPIAN Study. Child Care Health Dev. 2008; 34(1): 44-54.
Ministry of Health and Medical Education (Iran). Iran STEPS Noncommunicable Disease Risk Factors Survey 2011.
Ministry of Health and Medical Education (Iran), World Health Organization (WHO). Iran STEPS Noncommunicable Disease Risk Factors Survey 2006.
Dorosty AR, Siassi F, Reilly JJ. Obesity in Iranian children. Arch Dis Child. 2002; 87(5): 388-391.

Suggested citation; High body-mass index

Bakhshi E, Eshraghian MR, Mohammad K, Foroushani AR, Zeraati H, Fotouhi A, Siassi F, Seifi B. Sociodemographic and smoking associated with obesity in adult women in Iran: results from the National Health Survey. J Public Health (Oxf). 2008; 30(4): 429-35.

Kelishadi R, Gharipour M, Sadri GH, Tavasoli AA, Amani A. Cardiovascular disease risk factors, metabolic syndrome and obesity in an Iranian population. East Mediterr Health J. 2008; 14(5): 1070-9.

Fazizi F, Esmaillzadeh A, Mirmiran FP. Obesity and cardiovascular disease risk factors in Tehran adults: a population-based study. East Mediterr Health J. 2004; 10(6): 887-97.

Esmaillzadeh A, Mirmiran P, Azizi F. Comparative evaluation of anthropometric measures to predict cardiovascular risk factors in Tehranian adult women. Public Health Nutr. 2006; 9(1): 61-9.

Nabipour I, Amiri M, Imami SR, Jahfari SM, Nosrati A, Iranpour D, Soltanian AR. Unhealthy lifestyles and ischaemic electrocardiographic abnormalities: the Persian Gulf Healthy Heart Study. East Mediterr Health J. 2008; 14(4): 858-68.

Maddah M. Overweight and obesity among Iranian female adolescents in Rasht: more overweight in the lower social group. Public Health Nutr. 2007; 10(5): 450-3.

Hajian-Tilaki KO, Heidari B. Prevalence of obesity, central obesity and the associated factors in urban population aged 20-70 years, in the north of Iran: a population-based study and regression approach. Obes Rev. 2007; 8(1): 3-10.

Janghorbani M, Amini M, Rezvanian H, Gouya M-M, Delavari A, Alikhani S, Mahdavi A. Association of body mass index and abdominal obesity with marital status in adults. Arch Iran Med. 2008; 11(3): 274-81.

Azadbakht L, Esmaillzadeh A. Dietary and non-dietary determinants of central adiposity among Tehrani women. Public Health Nutr. 2008; 11(5): 528-34.

Maddah M, Nikooyeh B. Factors associated with overweight in children in Rasht, Iran: gender, maternal education, skipping breakfast and parental obesity. Public Health Nutr. 2010; 13(2): 196-200.

Maddah M, Nikooyeh B. Obesity among Iranian adolescent girls: location of residence and parental obesity. J Health Popul Nutr. 2010; 28(1): 61-6.

Esteghamati A, Meysamie A, Khalilzadeh O, Rashidi A, Haghazali M, Asgari F, Kamgar M, Gouya MM, Abbasi M. Third national Surveillance of Risk Factors of Non-Communicable Diseases (SuRFNCD-2007) in Iran: methods and results on prevalence of diabetes, hypertension, obesity, central obesity, and dyslipidemia. BMC Public Health. 2009; 167.

Soori H. Pattern of dietary behaviour and obesity in Ahwaz, Islamic Republic of Iran. East Mediterr Health J. 2001; 7(1-2): 163-70.

Fakhrzadeh H, Ghaderpanahi M, Sharifi F, Badamchizade Z, Mirarefin M, Larijani B. Increased risk of chronic kidney disease in elderly with metabolic syndrome and high levels of C-reactive protein: Kahrizak Elderly Study. Kidney Blood Press Res. 2009; 32(6): 457-63.

Azizi F, Azadbakht L, Mirmiran P. Trends in overweight, obesity and central fat accumulation among Tehranian adults between 1998-1999 and 2001-2002: Tehran lipid and glucose study. Ann Nutr Metab. 2005; 49(1): 3-8.

Khadivzadeh T. Mid upper arm and calf circumferences as indicators of nutritional status in women of reproductive age. East Mediterr Health J. 2002; 8(4-5): 612-8.

Nabipour I, Amiri M, Imami SR, Jahfari SM, Shafeiae E, Nosrati A, Iranpour D, Soltanian AR. The metabolic syndrome and nonfatal ischemic heart disease; a population-based study. Int J Cardiol. 2007; 118(1): 48-53.

Rashidy-Pour A, Malek M, Eskandarian R, Ghorbani R. Obesity in the Iranian population. Obes Rev. 2009; 10(1): 2-6.

Hamilton Health Sciences, McMaster University (Canada), Population Health Research Institute (PHRI). Iran Prospective Urban and Rural Epidemiological Study.

Maddah M, Akbarian Z, Shoyooie S, Rostamnejad M, Soleimani M. Prevalence of regular exercise among Iranian adults: a study in northern Iran. J Phys Act Health. 2014; 11(4): 810,Äì3.

Keshavarzi S, Ahmadi SM, Lankarani KB. The impact of depression and malnutrition on health-related quality of life among the elderly Iranians. Glob J Health Sci. 2015; 7(3): 161,Äì70.

Shab-Bidar S, Hosseini-Esfahani F, Mirmiran P, Hosseinpour-Niazi S, Azizi F. Metabolic syndrome profiles, obesity measures and intake of dietary fatty acids in adults: Tehran Lipid and Glucose Study. J Hum Nutr Diet. 2014; 27 Suppl 2: 98,Äì108.

Lotfi MH, Saadati H, Afzali M. Prevalence of diabetes in people aged ,â• 30 years: the results of screen-ing program of Yazd Province, Iran, in 2012. J Res Health Sci. 2014; 14(1): 87,Äì91.

Kheirandish M, Asgari S, Lotfaliany M, Bozorgmanesh M, Saadat N, Tohidi M, Azizi F, Hadaegh F. Secular trends in serum lipid levels of a Middle Eastern adult population; 10 years follow up in Tehran lipid and glucose study. Lipids Health Dis. 2014; 13: 20.

Safari M, Yazdanpanah B, Yazdanpanah B, Mobasheri A. A population-based screening of type 2 diabetes in high-risk population of Yasuj, Iran. J Health Popul Nutr. 2014; 32(4): 677,Äì86.

Suggested citation; High body-mass index

Massarrat M-S, Tahaghoghi-Mehrizi S. Iranian National Health Survey: A Brief report. Arch Iran Med. 2002; 5(2): 73,Äì9.

Kelishadi R, Heshmat R, Motlagh ME, Majdzadeh R, Keramatian K, Qorbani M, Taslimi M, Aminaee T, Ardalan G, Poursafa P, Larijani B. Methodology and Early Findings of the Third Survey of CASPIAN Study: A National School-based Surveillance of Students,Äô High Risk Behaviors. Int J Prev Med. 2012; 3(6): 394,Äì401.

Ministry of Health and Medical Education (Iran), Tehran University of Medical Sciences, United Nations Children's Fund (UNICEF). Iran National Integrated Micronutrient Survey 2012.

Ahadi Z, Qorbani M, Kelishadi R, Ardalan G, Motlagh ME, Asayesh H, Zeynali M, Chinian M, Larijani B, Shafiee G, Heshmat R. Association between breakfast intake with anthropometric measurements, blood pressure and food consumption behaviors among Iranian children and adolescents: the CASPIAN-IV study. Public Health. 2015; 129(6): 740,Äì7.

Ostovar A, Nabipour I, Larijani B, Heshmat R, Darabi H, Vahdat K, Ravanipour M, Mehrdad N, Raeisi A, Heidari G, Shafiee G, Haeri M, Pourbehi M, Sharifi F, Noroozi A, Tahmasebi R, Aghaei Meybodi H, Assadi M, Farrokhi S, Nemati R, Amini MR, Barekat M, Amini A, Salimipour H, Dobaradaran S, Moshtaghi D. Bushehr Elderly Health (BEH) Programme, phase I (cardiovascular system). BMJ Open. 2015; 5(12): e009597.

Ostovaneh MR, Zamani F, Ansari-Moghaddam A, Sharafkhah M, Saeedian FS, Rohani Z, Khoonsari M, Barzin G, Hemmasi G, Ajdarkosh H, Katoonizadeh A, Maadi M, Malekzadeh R, Poustchi H. Nonalcoholic Fatty Liver: The Association with Metabolic Abnormalities, Body Mass Index and Central Obesity--A Population-Based Study. Metab Syndr Relat Disord. 2015; 13(7): 304,Äì11.

International Agency for Research on Cancer (IARC), National Cancer Institute (NCI) (United States), Tehran University of Medical Sciences. Iran - Golestan Cohort Study 2004-2008 - Teheran University of Medical Sciences.

Digestive Diseases Research Institute (DDRI) (Iran), Shiraz University of Medical Sciences. Iran - Pars Cohort Study.

Mirbolouk M, Asgari S, Sheikholeslami F, Mirbolouk F, Azizi F, Hadaegh F. Different obesity phenotypes, and incident cardiovascular disease and mortality events in elderly Iranians: Tehran lipid and glucose study. Geriatr Gerontol Int. 2015; 15(4): 449-56.

Hosseinpanah F, Rambod M, Azizi F. Population attributable risk for diabetes associated with excess weight in Tehranian adults: a population-based cohort study. BMC Public Health. 2007; 7: 328.

Supplementary Table 4

Suggested citation; High LDL cholesterol
World Health Organization (WHO), Ministry of Health and Medical Education (Iran), Center for Non-Communicable Diseases Control (Iran). Iran STEPS Noncommunicable Disease Risk Factors Survey 2005.
World Health Organization (WHO), Ministry of Health and Medical Education (Iran), Center for Non-Communicable Diseases Control (Iran). Iran STEPS Noncommunicable Disease Risk Factors Survey 2007.
Ministry of Health and Medical Education (Iran), World Health Organization (WHO). Iran STEPS Noncommunicable Disease Risk Factors Survey 2009.
Ministry of Health and Medical Education (Iran). Iran Health and Disease Survey 1997-1999.
Amini M, Afshin-Nia F, Bashardoost N, Aminorroaya A, Shahparian M, Kazemi M. Prevalence and risk factors of diabetes mellitus in the Isfahan city population (aged 40 or over) in 1993. Diabetes Res Clin Pract. 1997; 38(3): 185-90.
Fahimfar N, Khalili D, Mohebi R, Azizi F, Hadaegh F. Risk factors for ischemic stroke; results from 9 years of follow-up in a population based cohort of Iran. BMC Neurol. 2012; 12: 117.
Shirani S, Heidari K, Sabzghabaee AM, Mirmoghtadaee P, Hoseini L, Aalifar H, Fadaei H, Esnaashari H, Soltani R. The modifiable noncommunicable risk factors among an Iranian population. Southeast Asian J Trop Med Public Health. 2012; 43(5): 1227-32.
Ministry of Health and Medical Education (Iran). Iran STEPS Noncommunicable Disease Risk Factors Survey 2011.
Bushehr University of Medical Sciences. Iran Persian Gulf Healthy Heart Study (PGHHS) Phase I 2003-2004.
Ministry of Health and Medical Education (Iran), World Health Organization (WHO). Iran STEPS Noncommunicable Disease Risk Factors Survey 2006.
Azizi F, Rahmani M, Ghanbarian A, Emami H, Salehi P, Mirmiran P, Sarbazi N. Serum lipid levels in an Iranian adults population: Tehran Lipid and Glucose Study. Eur J Epidemiol. 2003; 18(4): 311-9.
Rafiei M, Boshtam M, Sarraf-Zadegan N. Lipid profiles in the Isfahan population: an Isfahan cardiovascular disease risk factor survey, 1994. East Mediterr Health J. 1999; 5(4): 766-77.
Shirani S, Kelishadi R, Sarrafzadegan N, Khosravi A, Sadri G, Amani A, Heidari S, Ramezani MA. Awareness, treatment and control of hypertension, dyslipidaemia and diabetes mellitus in an Iranian population: the IHHP study. East Mediterr Health J. 2009; 15(6): 1455,Äì63.
Shab-Bidar S, Hosseini-Esfahani F, Mirmiran P, Hosseinpour-Niazi S, Azizi F. Metabolic syndrome profiles, obesity measures and intake of dietary fatty acids in adults: Tehran Lipid and Glucose Study. J Hum Nutr Diet. 2014; 27 Suppl 2: 98,Äì108.
Kheirandish M, Asgari S, Lotfaliany M, Bozorgmanesh M, Saadat N, Tohidi M, Azizi F, Hadaegh F. Secular trends in serum lipid levels of a Middle Eastern adult population; 10 years follow up in Tehran lipid and glucose study. Lipids Health Dis. 2014; 13: 20.
Safari M, Yazdanpanah B, Yazdanpanah B, Mobasheri A. A population-based screening of type 2 diabetes in high-risk population of Yasuj, Iran. J Health Popul Nutr. 2014; 32(4): 677,Äì86.
Ostovaneh MR, Zamani F, Sharafkhah M, Ansari-Moghaddam A, Akhavan Khaleghi N, Saeedian FS, Rohani Z, Motamed N, Maadi M, Malekzadeh R, Poustchi H. Prevalence of metabolic syndrome in Amol and Zahedan, Iran: a population based study. Arch Iran Med. 2014; 17(7): 477,Äì82.
Azimi-Nezhad M, Herbeth B, Siest G, Dad√© S, Ndiaye NC, Esmaily H, Hosseini SJ, Ghayour-Mobarhan M, Visvikis-Siest S. High prevalence of metabolic syndrome in Iran in comparison with France: what are the components that explain this?. Metab Syndr Relat Disord. 2012; 10(3): 181,Äì8.
Hadaegh F, Hatami M, Tohidi M, Sarbakhsh P, Saadat N, Azizi F. Lipid ratios and appropriate cut off values for prediction of diabetes: a cohort of Iranian men and women. Lipids Health Dis. 2010; 9: 85.
Hadaegh F, Shafiee G, Ghasemi A, Sarbakhsh P, Azizi F. Impact of metabolic syndrome, diabetes and prediabetes on cardiovascular events: Tehran lipid and glucose study. Diabetes Res Clin Pract. 2010; 87(3): 342,Äì7.
Haghighatdoost F, Sarrafzadegan N, Mohammadifard N, Sajjadi F, Maghroon M, Boshtam M, Alikhasi H, Azadbakht L. Healthy eating index and cardiovascular risk factors among Iranians. J Am Coll Nutr. 2013; 32(2): 111,Äì21.
Janghorbani M, Amini M. Associations of hip circumference and height with incidence of type 2 diabetes: the Isfahan diabetes prevention study. Acta Diabetol. 2012; 49 Suppl 1: S107,Äì114.
Hadaegh F, Shafiee G, Hatami M, Azizi F. Systolic and diastolic blood pressure, mean arterial pressure and pulse pressure for prediction of cardiovascular events and mortality in a Middle Eastern population. Blood Press. 2012; 21(1): 12,Äì8.
Hosseini-Esfahani F, Mousavi Nasl Khameneh A, Mirmiran P, Ghanbarian A, Azizi F. Trends in risk factors for cardiovascular disease among Iranian adolescents: the Tehran Lipid and Glucose Study, 1999-2008. J Epidemiol. 2011; 21(5): 319,Äì28.
Ghasemi A, Zahediasl S, Syedmoradi L, Azizi F. Association between serum nitric oxide metabolites and hypertension in a general population. Int Angiol. 2011; 30(4): 380,Äì7.

Suggested citation; High LDL cholesterol

Ostovar A, Nabipour I, Larijani B, Heshmat R, Darabi H, Vahdat K, Ravanipour M, Mehrdad N, Raeisi A, Heidari G, Shafiee G, Haeri M, Pourbehi M, Sharifi F, Noroozi A, Tahmasebi R, Aghaei Meybodi H, Assadi M, Farrokhi S, Nemati R, Amini MR, Barekat M, Amini A, Salimipour H, Dobaradaran S, Moshtaghi D. Bushehr Elderly Health (BEH) Programme, phase I (cardiovascular system). BMJ Open. 2015; 5(12): e009597.
Khosravi-Boroujeni H, Ahmed F, Sadeghi M, Roohafza H, Talaei M, Dianatkhah M, Pourmogaddas A, Sarrafzadegan N. Does the impact of metabolic syndrome on cardiovascular events vary by using different definitions?. BMC Public Health. 2015; 15: 1313.
Aalami Harandi S, Sarrafzadegan N, Sadeghi M, Talaei M, Dianatkhah M, Oveisgharan S, Pourmoghaddas A, Salehi A, Sedighifard Z. Do Cardiometabolic Risk Factors Relative Risks Differ for the Occurrence of Ischemic Heart Disease and Stroke?. Res Cardiovasc Med. 2016; 5(1): e30619.

Supplementary Table 5

Province	Measure	Attributed age-standardized rate (per 100,000)						% Change (1990 to 2019)		
		1990			2019					
		Both	Female	Male	Both	Female	Male	Both	Female	Male
Alborz	Deaths	239 (184.6 to 297.8)	229.7 (165.2 to 296.8)	245.7 (177.9 to 324)	168.5 (136.8 to 202.3)	173.9 (131.7 to 216.7)	172.4 (134.7 to 214.7)	-29.5 (-43.4 to -9.7)	-24.3 (-44.3 to 11.3)	-29.8 (-47.8 to -3.3)
	DALYs	4609 (3555.5 to 5719.7)	4150.7 (2969.2 to 5357.6)	4981.1 (3524.8 to 6647.7)	3111.7 (2594.3 to 3669.9)	2753.6 (2186.1 to 3379)	3531.1 (2843.2 to 4375.2)	-32.5 (-46.6 to -12.5)	-33.7 (-51.3 to -2.4)	-29.1 (-48.4 to 1.9)
	YLLs	4326.9 (3308.7 to 5398.6)	3854.7 (2673.3 to 5029.1)	4711.2 (3298.5 to 6333.1)	2819.3 (2324 to 3370.3)	2461.6 (1933.2 to 3048.2)	3238.1 (2547.8 to 4074.5)	-34.8 (-49.1 to -13.7)	-36.1 (-54 to -3.5)	-31.3 (-50.8 to 1.3)
	YLDs	282.1 (201.6 to 372.3)	296 (211 to 393.6)	269.9 (195 to 359.6)	292.4 (210.1 to 380.1)	292.1 (206.2 to 384.1)	293 (212.4 to 387.9)	3.7 (-6.8 to 15.6)	-1.3 (-18.1 to 17.2)	8.6 (-4.1 to 23.1)
Ardebil	Deaths	262.8 (212.1 to 316.9)	239.5 (181 to 299.2)	283 (215.6 to 358.8)	219.8 (184.7 to 254)	180.7 (143.4 to 216)	261.7 (217.7 to 307.3)	-16.4 (-32 to 2.9)	-24.6 (-42.3 to -1.3)	-7.5 (-27.7 to 20.4)
	DALYs	5231.3 (4252.6 to 6333.2)	4634 (3564.7 to 5780.8)	5747.8 (4381.6 to 7299.4)	4099.4 (3578.4 to 4675.3)	3228.4 (2669.3 to 3782.9)	5012.5 (4216.8 to 5807.5)	-21.6 (-36.6 to -3.6)	-30.3 (-46.5 to -9)	-12.8 (-33.4 to 15.7)
	YLLs	4952.9 (4000.3 to 6004.4)	4335.7 (3312.7 to 5472.4)	5487.5 (4124.9 to 7031.4)	3767.8 (3247.6 to 4283.6)	2891.8 (2372.8 to 3412.2)	4685.6 (3919.7 to 5433.9)	-23.9 (-38.9 to -5.5)	-33.3 (-49.5 to -11.6)	-14.6 (-35.5 to 14.4)
	YLDs	278.4 (200.9 to 363.5)	298.3 (212.5 to 393.4)	260.3 (188.6 to 344.6)	331.6 (240.1 to 432.4)	336.6 (242.9 to 441.4)	326.9 (236 to 426.1)	19.1 (6.8 to 33.6)	12.8 (-4.8 to 34.2)	25.6 (10 to 45.1)
Bushehr	Deaths	241 (184.9 to 296.8)	221.4 (160.3 to 284.6)	258.6 (187.5 to 330.7)	174.1 (141 to 206.5)	149.1 (114.4 to 186.7)	199.7 (161.1 to 240.6)	-27.8 (-42 to -9.5)	-32.7 (-50.3 to -9.8)	-22.8 (-41.7 to 3.7)
	DALYs	4838.9 (3765.5 to 5973.5)	4280.4 (3103.6 to 5436.9)	5349.3 (3858.4 to 6919.9)	3254.8 (2724.7 to 3771)	2680 (2159.5 to 3235.8)	3829.1 (3116.5 to 4555.8)	-32.7 (-46.5 to -14.6)	-37.4 (-52.8 to -14.6)	-28.4 (-47 to -0.1)
	YLLs	4576.9 (3535.9 to 5688)	4001.9 (2850.9 to 5134.9)	5103.2 (3645.3 to 6653.9)	2988.8 (2477.2 to 3486.2)	2410.2 (1924.5 to 2935.8)	3565.8 (2892.2 to 4251)	-34.7 (-48.7 to -16.5)	-39.8 (-55.2 to -16.6)	-30.1 (-48.7 to -0.7)
	YLDs	262 (187.9 to 345.4)	278.5 (193.9 to 368.8)	246.1 (172.9 to 332.7)	266.1 (190.9 to 350.1)	269.8 (193.5 to 357.3)	263.3 (185.4 to 347.8)	1.6 (-10.2 to 15.1)	-3.1 (-20 to 20.8)	7 (-7.2 to 22.7)

Province	Measure	Attributed age-standardized rate (per 100,000)						% Change (1990 to 2019)		
		1990			2019					
		Both	Female	Male	Both	Female	Male	Both	Female	Male
Chahar Mahaal and Bakhtiari	Deaths	242.5 (193 to 297.5)	215.4 (161.6 to 276.4)	266.4 (197.9 to 342.4)	169.7 (134.3 to 207.1)	135.1 (99.6 to 177)	206 (162.9 to 249.6)	-30 (-43.4 to -12.5)	-37.3 (-54.5 to -12.2)	-22.7 (-41.1 to 1.7)
	DALYs	4711 (3808.9 to 5720.9)	4008.9 (3054.4 to 5080)	5313.9 (3950.1 to 6957.5)	3106.1 (2607.2 to 3691.8)	2340.2 (1819.5 to 2986.8)	3897.2 (3188 to 4696.1)	-34.1 (-46.6 to -17.4)	-41.6 (-57.2 to -19)	-26.7 (-45.2 to -2.3)
	YLLs	4479.5 (3591.1 to 5461.8)	3767.1 (2834.2 to 4805.6)	5092.4 (3731.8 to 6695.3)	2848.3 (2355 to 3408)	2079.9 (1594.1 to 2716.6)	3642.4 (2966.4 to 4404.5)	-36.4 (-49 to -19.2)	-44.8 (-60.4 to -22.1)	-28.5 (-47.1 to -3.6)
	YLDs	231.5 (165.8 to 308.7)	241.8 (169.7 to 327)	221.5 (157.3 to 295.1)	257.9 (186.2 to 340.6)	260.3 (186.1 to 341.7)	254.8 (182.9 to 337.6)	11.4 (-0.9 to 25.2)	7.6 (-11.1 to 29.7)	15 (1 to 30.9)
East Azarbayejan	Deaths	303.7 (241.3 to 372)	292.1 (222.9 to 372)	311.2 (234.9 to 389.9)	237.1 (198.3 to 276.6)	231.5 (185.1 to 282.5)	242.8 (196.9 to 294.5)	-21.9 (-37.6 to -4.3)	-20.8 (-41.8 to 5.4)	-22 (-41.5 to 4.4)
	DALYs	5650.3 (4525.9 to 6934.1)	5141.6 (3966.4 to 6574.4)	6080.8 (4566.4 to 7802.8)	4031.2 (3442.6 to 4654.9)	3637.8 (3004.1 to 4390.5)	4423.7 (3605.8 to 5389.1)	-28.7 (-43 to -9.7)	-29.2 (-47.4 to -6)	-27.3 (-46.3 to 0)
	YLLs	5357.9 (4244.8 to 6624.7)	4829 (3663.6 to 6223.2)	5806.9 (4295.5 to 7522.1)	3723.6 (3141.7 to 4313.3)	3327.9 (2704.7 to 4051.7)	4118.1 (3318.7 to 5082.1)	-30.5 (-45.3 to -11.3)	-31.1 (-49.6 to -7.1)	-29.1 (-48.6 to -1)
	YLDs	292.5 (211.7 to 382.3)	312.6 (223.7 to 409.8)	274 (195.2 to 356.9)	307.6 (222.4 to 401.1)	309.8 (220.4 to 405.2)	305.5 (222.1 to 398.1)	5.2 (-4.5 to 16.3)	-0.9 (-15.9 to 17.1)	11.5 (0.2 to 26.2)
Fars	Deaths	263 (201 to 322.3)	249.6 (180.4 to 317)	270.3 (196.1 to 350.7)	186.7 (152.1 to 225.9)	186.7 (142.8 to 233.6)	188.5 (149 to 237.7)	-29 (-43.4 to -8)	-25.2 (-45 to 2.4)	-30.3 (-49.2 to -2.6)
	DALYs	5084.5 (3942.9 to 6215)	4482.9 (3278.9 to 5708.6)	5581.2 (4031.3 to 7313.6)	3349.3 (2820.9 to 3988.2)	3004.9 (2380.9 to 3674.3)	3701.1 (2970.7 to 4634.2)	-34.1 (-47.6 to -15.2)	-33 (-50.9 to -5.1)	-33.7 (-52.2 to -5.5)
	YLLs	4825.9 (3716.6 to 5952.5)	4203.5 (3021.3 to 5389.2)	5343.2 (3859.1 to 7059.9)	3081.2 (2578 to 3700.5)	2726.3 (2124.4 to 3366.2)	3443.9 (2724.4 to 4325)	-36.2 (-49.8 to -16.4)	-35.1 (-53.4 to -7.2)	-35.5 (-54.4 to -7.4)
	YLDs	258.6 (186.1 to 339.6)	279.4 (199.4 to 368.8)	238 (169.4 to 318.7)	268 (192.1 to 353.4)	278.6 (197.4 to 372.2)	257.2 (184.2 to 340.1)	3.7 (-8 to 18.1)	-0.3 (-16.4 to 19)	8.1 (-6.2 to 24.4)

Province	Measure	Attributed age-standardized rate (per 100,000)						% Change (1990 to 2019)		
		1990			2019					
		Both	Female	Male	Both	Female	Male	Both	Female	Male
Gilan	Deaths	280 (223.2 to 339.1)	265.4 (199.8 to 339.3)	288.6 (218.2 to 369.6)	200.5 (166.7 to 236)	205.4 (160.2 to 255.1)	197.4 (159.2 to 242.8)	-28.4 (-41.7 to -9.9)	-22.6 (-42.8 to 7.2)	-31.6 (-48.8 to -6.5)
	DALYs	5519.5 (4428.3 to 6680.8)	4976.9 (3731.1 to 6381.7)	5991.1 (4479.1 to 7768.8)	3713.6 (3194.7 to 4286.1)	3462.1 (2791.1 to 4184.7)	3978.7 (3263.3 to 4833.8)	-32.7 (-45.3 to -15.2)	-30.4 (-48.4 to -4.6)	-33.6 (-51 to -6.8)
	YLLs	5238.6 (4196.3 to 6353.5)	4676.3 (3473.3 to 6057.5)	5731.5 (4255.7 to 7517.9)	3413.1 (2914.1 to 3975.7)	3152.7 (2517.1 to 3838.3)	3687.2 (3004.9 to 4535.9)	-34.8 (-47.7 to -16.8)	-32.6 (-50.5 to -5.4)	-35.7 (-53.3 to -7.6)
	YLDs	280.9 (202.4 to 372)	300.6 (213.3 to 407.3)	259.6 (183.2 to 347)	300.6 (219.8 to 392.6)	309.4 (222.7 to 408.2)	291.5 (209.6 to 385.2)	7 (-4.7 to 20)	2.9 (-14.3 to 23.4)	12.3 (-1.5 to 28.3)
Golestan	Deaths	281.9 (227.2 to 342)	263.2 (198.2 to 328.9)	297.2 (226.6 to 377.4)	228 (191.6 to 263.7)	217.1 (174.4 to 258.5)	240.5 (194.9 to 292.9)	-19.1 (-32.4 to -1.6)	-17.5 (-35.9 to 10.1)	-19.1 (-37.6 to 6.1)
	DALYs	5707.3 (4560.1 to 6955.4)	5145.6 (3908.3 to 6388.3)	6196.3 (4700.5 to 7856)	4518.3 (3883.9 to 5162.5)	4103 (3369.6 to 4823.1)	4954.7 (4046.3 to 5947.5)	-20.8 (-35 to -2)	-20.3 (-39.2 to 7.5)	-20 (-39.8 to 7.5)
	YLLs	5419.7 (4293.7 to 6645.8)	4830.4 (3615.5 to 6057.3)	5936.1 (4446 to 7594.2)	4205 (3580 to 4840.6)	3769.1 (3075.7 to 4458.6)	4663.6 (3790.5 to 5603.9)	-22.4 (-36.6 to -2.8)	-22 (-41.6 to 6.2)	-21.4 (-41.6 to 6.8)
	YLDs	287.6 (203.4 to 381.7)	315.2 (217.9 to 425)	260.3 (184.2 to 345.4)	313.3 (225.7 to 408.5)	333.8 (240.1 to 440.2)	291.1 (210.2 to 385.4)	8.9 (-3.7 to 22.4)	5.9 (-11.9 to 28.1)	11.8 (-1.5 to 27.3)
Hamadan	Deaths	240.3 (188.4 to 300.7)	229.7 (169.6 to 295.6)	246.7 (183.6 to 331.6)	167.7 (133.8 to 204.3)	162.7 (122.6 to 210)	176.6 (135.6 to 221.3)	-30.2 (-43.6 to -12.3)	-29.2 (-46.2 to -2.2)	-28.4 (-47.5 to -1.2)
	DALYs	4779.1 (3754.1 to 5989.8)	4209.2 (3115.4 to 5408.9)	5271.2 (3927.6 to 7193.5)	3246.2 (2650.8 to 3854)	2790.5 (2210.5 to 3464.1)	3731 (2935.5 to 4646.5)	-32.1 (-45.5 to -14.8)	-33.7 (-50.7 to -8.7)	-29.2 (-48.4 to -1.8)
	YLLs	4557.3 (3570.6 to 5765)	3971.5 (2917.4 to 5114.8)	5064.7 (3749.9 to 6929.4)	3001.5 (2431 to 3586.6)	2540.9 (1995.7 to 3201.8)	3491.1 (2716 to 4389.2)	-34.1 (-47.8 to -16.1)	-36 (-53.2 to -10.7)	-31.1 (-50.3 to -3)
	YLDs	221.8 (157.3 to 293.8)	237.8 (164.2 to 324.5)	206.6 (146.3 to 274)	244.7 (177.2 to 322.8)	249.7 (176.7 to 335.1)	239.9 (172.2 to 321.1)	10.4 (-2.8 to 26.6)	5 (-15.9 to 31.9)	16.1 (0.1 to 35.2)

Province	Measure	Attributed age-standardized rate (per 100,000)						% Change (1990 to 2019)		
		1990			2019					
		Both	Female	Male	Both	Female	Male	Both	Female	Male
Hormozgan	Deaths	300.2 (230 to 359.4)	280.1 (214.9 to 347.7)	310.6 (221 to 401.7)	181.4 (150.2 to 212.5)	188.6 (152.5 to 227)	180.2 (141.3 to 219.7)	-39.6 (-50.8 to -22)	-32.7 (-47.4 to -11.8)	-42 (-56.6 to -17.2)
	DALYs	6179.4 (4505.6 to 7493.5)	5437.7 (3991.6 to 6828.7)	6724 (4666.6 to 8786.5)	3665.8 (3149.2 to 4255.8)	3316.1 (2753 to 3901.3)	4062.1 (3259.9 to 4969.9)	-40.7 (-52.4 to -19.9)	-39 (-52.9 to -18.1)	-39.6 (-55.7 to -10.8)
	YLLs	5916.1 (4251.1 to 7212)	5152.8 (3733.6 to 6534.6)	6482.2 (4484.5 to 8560.4)	3375.7 (2863.5 to 3936.3)	3013.9 (2481.4 to 3584.1)	3783.3 (3010.4 to 4658)	-42.9 (-54.9 to -21.5)	-41.5 (-55.5 to -20.1)	-41.6 (-57.8 to -12.3)
	YLDs	263.3 (187.7 to 346)	284.9 (200.5 to 375)	241.8 (172.5 to 322.6)	290.1 (210.2 to 375.9)	302.2 (217.4 to 391.5)	278.8 (201.5 to 368.6)	10.2 (-0.7 to 22.2)	6.1 (-10.7 to 25.8)	15.3 (1.7 to 31.8)
Ilam	Deaths	248 (196.5 to 305)	232.1 (175.1 to 290.3)	259.3 (198.1 to 333.6)	190.4 (155.7 to 224.6)	184.3 (142.2 to 224.4)	194.9 (154.7 to 236.3)	-23.2 (-35.4 to -6.5)	-20.6 (-38.7 to 5.9)	-24.9 (-41.4 to -0.2)
	DALYs	4838.9 (3785.9 to 5888.5)	4316.9 (3251.5 to 5401.1)	5233.9 (3921.3 to 6748.5)	3455.4 (2929.8 to 3955.3)	3219.4 (2616.8 to 3870.9)	3667.5 (2986.8 to 4388.5)	-28.6 (-40.8 to -11.9)	-25.4 (-42.5 to 0.6)	-29.9 (-45.8 to -5.8)
	YLLs	4617.7 (3579 to 5649.3)	4085.1 (3046.9 to 5155)	5020.8 (3744.8 to 6542.2)	3203.6 (2695 to 3699.7)	2958.9 (2383.9 to 3587)	3424.5 (2763.2 to 4139.5)	-30.6 (-42.7 to -13.5)	-27.6 (-44.5 to -0.7)	-31.8 (-47.6 to -6.8)
	YLDs	221.2 (157.6 to 294.6)	231.8 (161.7 to 313.2)	213.1 (149.1 to 284.1)	251.8 (181.3 to 328.9)	260.5 (187.2 to 343.6)	243.1 (173.3 to 320.6)	13.8 (1.8 to 28.6)	12.4 (-6.8 to 37.8)	14.1 (-0.9 to 32.4)
Isfahan	Deaths	259.9 (205 to 315.8)	262.2 (201.6 to 330.5)	246.9 (181.5 to 318.4)	138.4 (110.8 to 167.3)	145.8 (108.8 to 184.8)	133.7 (102.5 to 169.7)	-46.7 (-57.1 to -32.2)	-44.4 (-58.6 to -24.2)	-45.9 (-60.5 to -24.1)
	DALYs	4778.3 (3780.8 to 5804.5)	4445.8 (3384.9 to 5704)	4972.3 (3705.6 to 6490.7)	2536.9 (2089.8 to 3001.1)	2344.6 (1846.5 to 2894.4)	2739.2 (2154.4 to 3442.6)	-46.9 (-57.6 to -32)	-47.3 (-60.5 to -27.3)	-44.9 (-60.2 to -21.9)
	YLLs	4505.3 (3522.7 to 5549.2)	4154 (3080 to 5415.5)	4719.8 (3496.5 to 6223.6)	2305.2 (1877.6 to 2758.7)	2110.1 (1633.6 to 2642.2)	2510.2 (1943.4 to 3184.2)	-48.8 (-59.4 to -33.1)	-49.2 (-62.7 to -28.4)	-46.8 (-62.1 to -23)
	YLDs	273 (196.2 to 356.5)	291.8 (208.7 to 382.1)	252.6 (182.3 to 333.8)	231.8 (166 to 306.3)	234.5 (165.9 to 313.1)	229.1 (163.9 to 304.2)	-15.1 (-22.9 to -8)	-19.6 (-30.7 to -8.8)	-9.3 (-19.5 to 0.2)

Province	Measure	Attributed age-standardized rate (per 100,000)						% Change (1990 to 2019)		
		1990			2019					
		Both	Female	Male	Both	Female	Male	Both	Female	Male
Kerman	Deaths	305 (246.7 to 366.1)	272.4 (207.6 to 342.1)	332.9 (253.3 to 414.9)	189 (153 to 223.9)	189.3 (147.5 to 231.3)	189.1 (150.1 to 229)	-38 (-48.6 to -23.7)	-30.5 (-47.9 to -9.4)	-43.2 (-55.6 to -26.4)
	DALYs	6104.9 (4926.3 to 7322.8)	5101.8 (3930.5 to 6375.6)	6990.4 (5348.4 to 8800.5)	3383.5 (2830.7 to 3971.3)	3083.2 (2497.4 to 3677.2)	3677.9 (3006.6 to 4431)	-44.6 (-54.6 to -30.7)	-39.6 (-54 to -20.9)	-47.4 (-59.7 to -29.8)
	YLLs	5825.1 (4672.2 to 7027.8)	4806.3 (3676.7 to 6057.2)	6726.1 (5096.4 to 8543.5)	3104.8 (2561.1 to 3660.8)	2795.4 (2250.4 to 3349)	3408.6 (2756.6 to 4161.1)	-46.7 (-57 to -32.2)	-41.8 (-56.4 to -22.5)	-49.3 (-61.6 to -31.5)
	YLDs	279.8 (201.6 to 367.1)	295.5 (214.2 to 392.5)	264.3 (189.6 to 347.8)	278.6 (204.2 to 364.6)	287.9 (207.6 to 374.8)	269.4 (193.5 to 354.5)	-0.4 (-9.9 to 10.9)	-2.6 (-17.8 to 14.1)	1.9 (-8.6 to 14.7)
Kermanshah	Deaths	288.3 (231.6 to 347.7)	252.9 (188.6 to 319.4)	315.9 (239.3 to 401.1)	184.8 (151.8 to 220.5)	184 (141.9 to 228.6)	188.7 (149.9 to 234.2)	-35.9 (-48.4 to -19.1)	-27.3 (-46.5 to -1.8)	-40.3 (-55.2 to -18.8)
	DALYs	5905.8 (4715.4 to 7213)	4835.7 (3607.5 to 6121.2)	6780.9 (5087.5 to 8772.9)	3612.8 (3047.5 to 4234)	3259.6 (2597.1 to 3955.8)	4000.2 (3235.5 to 4939.7)	-38.8 (-51.1 to -21.1)	-32.6 (-50.2 to -6.5)	-41 (-56.5 to - 18.4)
	YLLs	5656.9 (4488.2 to 6923.6)	4573.5 (3380.7 to 5850.7)	6543.7 (4867.4 to 8518.3)	3325.8 (2809 to 3909.1)	2968.1 (2344 to 3649.7)	3717.8 (2961 to 4637.3)	-41.2 (-53.5 to -23.6)	-35.1 (-52.5 to -9.1)	-43.2 (-58.6 to -20.2)
	YLDs	248.9 (178.4 to 330.7)	262.2 (185.3 to 354.4)	237.2 (170 to 318.9)	287 (205.6 to 379.5)	291.5 (206.6 to 388.6)	282.4 (199.8 to 372.8)	15.3 (1.4 to 30.9)	11.2 (-8.7 to 38.1)	19.1 (3.8 to 37.3)
Khorasan-e-Razavi	Deaths	310.9 (248.8 to 377.1)	281 (213.5 to 361.3)	336.5 (256.9 to 425.2)	186.8 (155.4 to 218.8)	172.8 (133.3 to 214)	201.5 (161.8 to 241.3)	-39.9 (-50.9 to -26.4)	-38.5 (-55.7 to -16.4)	-40.1 (-54.1 to -22)
	DALYs	6136.9 (4925.7 to 7415.3)	5264.6 (4007.3 to 6757.5)	6907.1 (5235.2 to 8855.7)	3472.2 (2954.9 to 4017.8)	2968.7 (2372.7 to 3604.3)	3988.4 (3247.1 to 4776.4)	-43.4 (-54 to -29.1)	-43.6 (-59.1 to -23.7)	-42.3 (-57.1 to -22.2)
	YLLs	5858 (4655.1 to 7111.8)	4985.5 (3747.3 to 6440.8)	6628.2 (4984.3 to 8561.2)	3198.2 (2703.1 to 3729.4)	2709.4 (2137.5 to 3336.4)	3699.1 (2973.7 to 4474.3)	-45.4 (-56.1 to -31)	-45.7 (-61 to -25.7)	-44.2 (-59.2 to -24)
	YLDs	279 (201.4 to 367.9)	279.2 (193.7 to 374.8)	278.8 (199 to 367.1)	274 (194.1 to 359.3)	259.3 (182.9 to 344)	289.3 (204.8 to 380.8)	-1.8 (-11.3 to 9.4)	-7.1 (-22.7 to 12.8)	3.8 (-7.6 to 15.9)

Province	Measure	Attributed age-standardized rate (per 100,000)						% Change (1990 to 2019)		
		1990			2019					
		Both	Female	Male	Both	Female	Male	Both	Female	Male
Khuzestan	Deaths	283.4 (227.8 to 343.9)	261.3 (199.9 to 329.2)	301.2 (228.7 to 387.5)	203.1 (165.9 to 243.9)	195.8 (148.7 to 246.2)	210.9 (167.7 to 258.4)	-28.3 (-41.9 to -11.9)	-25.1 (-44.6 to 0.8)	-30 (-47.2 to -6)
	DALYs	5718.6 (4632.3 to 6961.7)	5007.4 (3886.3 to 6306.1)	6332.8 (4779.1 to 8267.3)	3924.1 (3323.6 to 4564.5)	3513.8 (2781.5 to 4291.2)	4333 (3541.1 to 5284.1)	-31.4 (-44.7 to -14.8)	-29.8 (-47.1 to -5.2)	-31.6 (-49.2 to -7.4)
	YLLs	5445.4 (4378.2 to 6651.6)	4720.1 (3643.9 to 5994)	6073.6 (4558.6 to 7992.3)	3610.4 (3054.4 to 4224.4)	3195.6 (2494.7 to 3969.2)	4023.8 (3251 to 4953.7)	-33.7 (-46.9 to -17)	-32.3 (-50 to -7.4)	-33.7 (-51.7 to -9.4)
	YLDs	273.2 (194.6 to 359)	287.4 (201.6 to 381.5)	259.2 (183.4 to 345.5)	313.7 (224.8 to 409.2)	318.2 (227 to 416.8)	309.2 (223.1 to 411.7)	14.8 (2.5 to 29.4)	10.7 (-7.4 to 32.5)	19.3 (4.9 to 34.6)
Kohgiluyeh and Boyer-Ahmad	Deaths	240.4 (184 to 295.1)	248.4 (178.7 to 318.6)	224.8 (165.6 to 296.3)	166.6 (129.5 to 207)	189 (140.1 to 242.3)	151.9 (111.6 to 200)	-30.7 (-46.1 to -12.3)	-23.9 (-45.8 to 5.2)	-32.4 (-52.1 to -5)
	DALYs	4559.4 (3528.3 to 5574.1)	4430.9 (3211.5 to 5678.1)	4565.3 (3321 to 5968.5)	3037.8 (2470.6 to 3663)	3163.9 (2423 to 3999.8)	2985.3 (2245.5 to 3888.3)	-33.4 (-47.8 to -14.4)	-28.6 (-48.5 to -1.1)	-34.6 (-54.4 to -6.1)
	YLLs	4325.9 (3313.3 to 5341.7)	4181.9 (3000.2 to 5384.8)	4346.6 (3146.4 to 5746.2)	2777.2 (2229.9 to 3386.4)	2884.9 (2176.4 to 3704.2)	2740.9 (2005.5 to 3612.3)	-35.8 (-50.3 to -16.5)	-31 (-51.3 to -3.2)	-36.9 (-56.9 to -7.8)
	YLDs	233.5 (164.9 to 311.8)	249 (173.2 to 336.7)	218.8 (156.2 to 291.2)	260.6 (187.5 to 339.4)	279 (202.4 to 368.1)	244.4 (173.5 to 323.8)	11.6 (-1.2 to 25.1)	12 (-6 to 36.7)	11.7 (-2.2 to 28.6)
Kurdistan	Deaths	266.3 (212.7 to 325.5)	229.4 (172.7 to 296.4)	296.8 (226.7 to 385.6)	169.5 (138.5 to 199.8)	162.5 (124.5 to 197.8)	178.9 (142.8 to 218.8)	-36.3 (-48.4 to -21.8)	-29.2 (-47.7 to -4.5)	-39.7 (-54.8 to -20.9)
	DALYs	5369.8 (4332.9 to 6571.1)	4447.6 (3362.3 to 5698)	6137.2 (4722.1 to 8035.9)	3249 (2773.1 to 3775.5)	2847.6 (2301.6 to 3422.7)	3673.6 (2985.1 to 4454.2)	-39.5 (-51.1 to -25.4)	-36 (-52.4 to -13.4)	-40.1 (-55.9 to -20)
	YLLs	5125.7 (4127.8 to 6288.5)	4197 (3139.8 to 5423.1)	5899 (4490.4 to 7760.3)	2974.1 (2527.4 to 3460.6)	2575.9 (2042.2 to 3109.1)	3395.6 (2734.4 to 4171)	-42 (-53.2 to -27.8)	-38.6 (-55.2 to -16.3)	-42.4 (-58.4 to -21.8)
	YLDs	244.1 (170.6 to 322.6)	250.6 (172.8 to 341)	238.2 (167 to 315.3)	274.9 (198.2 to 358.3)	271.8 (192 to 360.2)	278 (201.2 to 366.3)	12.6 (1.4 to 26.4)	8.4 (-9.7 to 31)	16.7 (3.8 to 33.6)

Province	Measure	Attributed age-standardized rate (per 100,000)						% Change (1990 to 2019)		
		1990			2019					
		Both	Female	Male	Both	Female	Male	Both	Female	Male
Lorestan	Deaths	262.6 (210.5 to 321.5)	235.4 (180 to 295.1)	284.8 (210.3 to 374.1)	193.4 (158.6 to 230.7)	151.4 (113.2 to 184.4)	237.2 (189.8 to 290.3)	-26.3 (-40.1 to -9)	-35.7 (-53.4 to -14.3)	-16.7 (-37 to 13.2)
	DALYs	5237.7 (4199.9 to 6446.1)	4479 (3425.4 to 5642.3)	5858.2 (4296.8 to 7724.3)	3636.3 (3041 to 4284.8)	2695.1 (2096.1 to 3258.6)	4626.6 (3740.5 to 5641.2)	-30.6 (-45.2 to -13)	-39.8 (-57.1 to -19.8)	-21 (-42 to 10.8)
	YLLs	5000.4 (3983.5 to 6223.1)	4228.1 (3213.3 to 5401.6)	5632.9 (4062.4 to 7473.5)	3380.7 (2800.1 to 4016.1)	2441.5 (1847.3 to 2964.9)	4368.6 (3504.1 to 5367.5)	-32.4 (-47 to -14.4)	-42.3 (-59.5 to -22.1)	-22.4 (-43.5 to 10.4)
	YLDs	237.3 (170.1 to 310.3)	250.9 (179.2 to 334.6)	225.3 (159.3 to 299.5)	255.6 (181.1 to 332.9)	253.6 (178.9 to 339.7)	258 (182.9 to 337.5)	7.7 (-4.2 to 21.1)	1.1 (-16.1 to 22.1)	14.5 (-0.3 to 31.3)
Markazi	Deaths	258.3 (206.7 to 312.9)	244.3 (186.6 to 312.4)	269 (204.3 to 345.8)	156 (128 to 183.4)	153.4 (120.3 to 186.8)	159.8 (126.5 to 196.7)	-39.6 (-51.7 to -25.2)	-37.2 (-53.9 to -16)	-40.6 (-55.3 to -20)
	DALYs	5140.2 (4102.3 to 6242)	4514.1 (3453.9 to 5808)	5722.6 (4340.6 to 7613)	2991.3 (2521.1 to 3484.1)	2629 (2124.7 to 3146.9)	3354.9 (2690 to 4099.3)	-41.8 (-53.3 to -26.5)	-41.8 (-57 to -21.9)	-41.4 (-56.6 to -19.1)
	YLLs	4868.3 (3839.7 to 5977.9)	4229.3 (3204.5 to 5501.6)	5463.6 (4052.4 to 7328.5)	2710.7 (2256.2 to 3179.2)	2351 (1882.9 to 2820.3)	3071.7 (2432.5 to 3799.8)	-44.3 (-55.8 to -28.7)	-44.4 (-59.8 to -24.1)	-43.8 (-59.1 to -21.3)
	YLDs	271.9 (193.1 to 356.2)	284.9 (201.3 to 379.2)	259 (184.9 to 338.1)	280.6 (204.1 to 364)	277.9 (196.7 to 362.1)	283.3 (206.6 to 373.7)	3.2 (-7.3 to 14.6)	-2.4 (-18.6 to 18.1)	9.4 (-2.4 to 23.2)
Mazandaran	Deaths	240.9 (191.7 to 297.6)	239.6 (179.6 to 302.6)	237.1 (176.9 to 310.1)	178.1 (147.8 to 210.5)	174.8 (135.2 to 214.4)	181.4 (145 to 221.9)	-26.1 (-40.3 to -6.6)	-27.1 (-45.1 to -0.8)	-23.5 (-44.1 to 5)
	DALYs	4655.6 (3744.6 to 5638.6)	4424.3 (3362.5 to 5577.1)	4828.9 (3558 to 6321)	3358.1 (2845.6 to 3889.5)	3063.7 (2477.6 to 3697.9)	3654.8 (2934.1 to 4394.1)	-27.9 (-41.2 to -9.2)	-30.8 (-47.7 to -7.4)	-24.3 (-45.6 to 3.8)
	YLLs	4373.1 (3504.4 to 5314.5)	4125.1 (3086.5 to 5265.4)	4563.1 (3333.2 to 6051.1)	3057.2 (2568.6 to 3580.9)	2760.7 (2200.3 to 3381.5)	3355.9 (2675.5 to 4100.2)	-30.1 (-44 to -10.8)	-33.1 (-50.3 to -8.7)	-26.5 (-48 to 3)
	YLDs	282.5 (203 to 373.4)	299.2 (213.3 to 395.9)	265.8 (190.6 to 353.6)	300.9 (218.2 to 387.5)	303.1 (218.6 to 393.1)	298.8 (215.1 to 390.1)	6.5 (-4.3 to 17.8)	1.3 (-13.6 to 19.4)	12.4 (-0.6 to 27.6)

Province	Measure	Attributed age-standardized rate (per 100,000)						% Change (1990 to 2019)		
		1990			2019					
		Both	Female	Male	Both	Female	Male	Both	Female	Male
North Khorasan	Deaths	293.9 (242.7 to 352.3)	286.4 (224.2 to 355)	296.6 (229 to 372.1)	195.6 (164.8 to 227.6)	209.3 (169.5 to 251)	186.3 (154.7 to 222.2)	-33.4 (-44.7 to -20.8)	-26.9 (-44.7 to -4.1)	-37.2 (-51.2 to -18.9)
	DALYs	5867.8 (4807.6 to 7106.8)	5477.6 (4335.9 to 6808.8)	6178.4 (4751.3 to 7849)	3765.2 (3224.3 to 4323.1)	3749.6 (3083.9 to 4433.3)	3817.7 (3175.9 to 4531.8)	-35.8 (-47.2 to -22.9)	-31.5 (-47.7 to -10.6)	-38.2 (-52.7 to -17.6)
	YLLs	5611.7 (4577.8 to 6812.4)	5203.1 (4068.6 to 6493.1)	5939.4 (4527.1 to 7582.7)	3473 (2949.7 to 4017.7)	3449 (2798.4 to 4074.7)	3534.3 (2922.5 to 4226.2)	-38.1 (-49.6 to -25.2)	-33.7 (-50 to -12.7)	-40.5 (-54.9 to -19.9)
	YLDs	256.1 (185.4 to 340)	274.5 (195.3 to 367.2)	239 (171.1 to 316.8)	292.2 (210.2 to 383.7)	300.6 (215.1 to 397.6)	283.3 (205.1 to 370.9)	14.1 (2.2 to 28.3)	9.5 (-8.2 to 32.1)	18.5 (4.3 to 35)
Qazvin	Deaths	213.2 (167.3 to 263.3)	192.5 (141.8 to 245)	233.9 (174.7 to 304.6)	153 (124.8 to 180.9)	122 (91.9 to 154.2)	186.2 (150.4 to 223.1)	-28.2 (-42 to -9.4)	-36.6 (-54.7 to -12)	-20.4 (-40 to 6.7)
	DALYs	4249.2 (3383.6 to 5234.6)	3698.4 (2693 to 4670.3)	4762.6 (3533.1 to 6358.5)	2885.6 (2437.4 to 3368.1)	2158.2 (1699.7 to 2663.4)	3639.4 (2991.3 to 4337.5)	-32.1 (-45.4 to -13.7)	-41.6 (-57.3 to -19.9)	-23.6 (-42.9 to 3.5)
	YLLs	4018.3 (3182 to 4983.7)	3458 (2497.1 to 4420.2)	4541.1 (3327.5 to 6098.5)	2630.8 (2178.8 to 3106.3)	1908.1 (1477.1 to 2388.4)	3379.4 (2745.6 to 4081.8)	-34.5 (-48.2 to -15.5)	-44.8 (-60.4 to -22.6)	-25.6 (-45.5 to 2.8)
	YLDs	230.9 (164.1 to 304.6)	240.3 (168.3 to 322.8)	221.4 (155 to 294)	254.8 (183.4 to 335.1)	250.2 (176.1 to 331.2)	260 (186.4 to 340.8)	10.4 (-1.1 to 25.3)	4.1 (-13.1 to 28.8)	17.4 (2.5 to 35)
Qom	Deaths	227.7 (176.2 to 278.2)	228.3 (166.2 to 296.9)	223 (158.1 to 285.7)	133 (106.7 to 162)	149.4 (111.2 to 191.5)	125.4 (98 to 156.7)	-41.6 (-54.6 to -24.5)	-34.6 (-52.6 to -8.8)	-43.8 (-58.1 to -21)
	DALYs	4341 (3297.3 to 5303.9)	4039.2 (2917.1 to 5213.9)	4568.3 (3302.3 to 5918.8)	2454.7 (2007.5 to 2916.6)	2427.4 (1884.9 to 3006)	2535.2 (2019.9 to 3145.9)	-43.5 (-55.4 to -25.7)	-39.9 (-56.1 to -17.6)	-44.5 (-59.1 to -20.5)
	YLLs	4105.7 (3066.1 to 5057)	3792.5 (2708.7 to 4905.4)	4343.9 (3098.1 to 5678.8)	2213.4 (1797.6 to 2646.3)	2186.6 (1692.6 to 2737.9)	2293.1 (1805.9 to 2867.5)	-46.1 (-58 to -28.5)	-42.3 (-58.6 to -19.6)	-47.2 (-61.6 to -23)
	YLDs	235.3 (169.5 to 307.7)	246.6 (171.3 to 330.3)	224.4 (160.3 to 300.3)	241.4 (176.2 to 317.7)	240.7 (171.6 to 322.2)	242.2 (173.6 to 321.3)	2.6 (-10 to 15.5)	-2.4 (-21 to 19.1)	7.9 (-6.4 to 25.4)

Province	Measure	Attributed age-standardized rate (per 100,000)						% Change (1990 to 2019)		
		1990			2019					
		Both	Female	Male	Both	Female	Male	Both	Female	Male
Semnan	Deaths	236.5 (184.5 to 291.5)	212.3 (157.4 to 276.5)	261.1 (198 to 334.3)	168.7 (138.9 to 200.4)	137.8 (104.8 to 171.3)	201.4 (165.4 to 240.2)	-28.7 (-42.6 to -10)	-35.1 (-53.4 to -12.4)	-22.9 (-40.2 to 2)
	DALYs	4677.7 (3652.6 to 5795.1)	4024.5 (2975.2 to 5203.2)	5319.7 (3935.5 to 6815.3)	3102.5 (2632.5 to 3612.6)	2393 (1890.1 to 2929.1)	3830.7 (3198 to 4516.3)	-33.7 (-46 to -15.4)	-40.5 (-57.4 to -18.1)	-28 (-45 to -2.6)
	YLLs	4412.5 (3415.2 to 5487)	3744.9 (2727 to 4848.4)	5069.3 (3721.6 to 6570.5)	2829 (2380.4 to 3319.3)	2121.5 (1642.7 to 2622.5)	3554.9 (2940.2 to 4209.6)	-35.9 (-48.5 to -16.9)	-43.3 (-60.1 to -20.3)	-29.9 (-47 to -4)
	YLDs	265.2 (189.7 to 346.5)	279.6 (200.5 to 372.5)	250.4 (175.4 to 336.2)	273.5 (196.7 to 357.8)	271.5 (193.7 to 357.4)	275.7 (197.9 to 362)	3.1 (-7.2 to 15.9)	-2.9 (-19 to 16.9)	10.1 (-3.5 to 25.3)
Sistan and Baluchistan	Deaths	239.8 (179.7 to 299.6)	218.8 (162.1 to 283.9)	256.7 (177.1 to 336.7)	167.2 (136 to 201.3)	164.1 (125.4 to 205.7)	171 (132.2 to 217.5)	-30.3 (-44.7 to -8.9)	-25 (-44.8 to 4.5)	-33.4 (-51.1 to -1.6)
	DALYs	5033 (3569.5 to 6296.6)	4423.1 (3241.5 to 5764.1)	5512.9 (3587 to 7374.6)	3618.1 (3003.2 to 4274.2)	3380.6 (2651.9 to 4190)	3869.9 (2995.7 to 4894.7)	-28.1 (-43.5 to 1.1)	-23.6 (-44.7 to 7.9)	-29.8 (-50.2 to 8.9)
	YLLs	4800 (3351.7 to 6046.2)	4177.3 (2999.4 to 5488.5)	5290.6 (3346.5 to 7114.6)	3375.2 (2777.5 to 4013.5)	3132.8 (2408.7 to 3931.1)	3631.5 (2772.8 to 4645.9)	-29.7 (-45.4 to 0.7)	-25 (-46.6 to 8.2)	-31.4 (-51.8 to 8.7)
	YLDs	233 (166.4 to 306.4)	245.7 (170 to 327.1)	222.3 (158.5 to 294.6)	242.9 (175.6 to 319.6)	247.8 (176.4 to 327)	238.4 (170.3 to 315.2)	4.3 (-7.7 to 17.6)	0.8 (-16.7 to 23.5)	7.2 (-6 to 24.4)
South Khorasan	Deaths	192.9 (152 to 236.2)	179.6 (133.3 to 228)	204 (155.1 to 264.8)	125.1 (100.9 to 151.4)	115.3 (87.9 to 145.9)	137 (108.2 to 168.5)	-35.2 (-48 to -19.8)	-35.8 (-52.8 to -12.2)	-32.8 (-49 to -10.5)
	DALYs	3788.1 (3010 to 4688.9)	3377.3 (2572.5 to 4300.8)	4146.7 (3118.2 to 5470)	2329.1 (1936 to 2793.1)	2039.1 (1634.1 to 2533.4)	2647.9 (2124.1 to 3265.2)	-38.5 (-49.7 to -23.9)	-39.6 (-54.8 to -17.9)	-36.1 (-52 to -14.5)
	YLLs	3562.9 (2821.2 to 4452)	3142.6 (2365.8 to 4037.2)	3930.2 (2891.6 to 5227)	2088.4 (1721.8 to 2534.4)	1806.7 (1425.6 to 2285.8)	2399 (1901.3 to 3026.9)	-41.4 (-52.6 to -26.6)	-42.5 (-57.3 to -20.4)	-39 (-54.8 to -16.7)
	YLDs	225.2 (159.4 to 301.5)	234.7 (160 to 324.1)	216.5 (152 to 293.4)	240.7 (173.9 to 319.1)	232.5 (163.5 to 312.8)	248.9 (178.4 to 329.3)	6.9 (-4.8 to 22.1)	-0.9 (-20.2 to 22.6)	15 (-0.3 to 32.4)

Province	Measure	Attributed age-standardized rate (per 100,000)						% Change (1990 to 2019)		
		1990			2019					
		Both	Female	Male	Both	Female	Male	Both	Female	Male
Tehran	Deaths	129.6 (97.6 to 167.1)	136.6 (101.6 to 184.5)	118.7 (85.2 to 163.2)	68.9 (53.2 to 89.1)	75.1 (54.8 to 102.1)	64.4 (47.8 to 83.5)	-46.8 (-59.2 to -31.4)	-45 (-61.6 to -22.2)	-45.8 (-61.2 to -23.6)
	DALYs	2401.7 (1866 to 3070.1)	2311.6 (1750.1 to 3059.2)	2424.1 (1798.8 to 3294.6)	1323 (1065.3 to 1634.7)	1269.9 (962.5 to 1668.9)	1388.1 (1086.4 to 1757.6)	-44.9 (-57 to -30.2)	-45.1 (-60.2 to -23.8)	-42.7 (-58.8 to -19.3)
	YLLs	2174.7 (1660.3 to 2833.8)	2072.7 (1534.8 to 2808.3)	2210.2 (1594.9 to 3066.2)	1118.7 (876 to 1419.1)	1068.9 (785.2 to 1456.4)	1180.2 (891.8 to 1529.7)	-48.6 (-60.9 to -32.7)	-48.4 (-64.3 to -25.6)	-46.6 (-63.1 to -22.5)
	YLDs	227 (163.8 to 295.7)	238.9 (170.4 to 312.6)	213.9 (152.1 to 282)	204.4 (146.3 to 270.7)	201.1 (140 to 271)	207.8 (148.9 to 277.2)	-10 (-20.3 to 1.3)	-15.9 (-30.9 to 0.4)	-2.8 (-14.7 to 10.5)
West Azarbayejan	Deaths	282.2 (226.3 to 347)	268.1 (201.4 to 340.7)	291.8 (220.2 to 374.7)	201.6 (163.1 to 240.6)	200.1 (153.6 to 250.4)	203.2 (160.1 to 248.4)	-28.5 (-42.5 to -12)	-25.4 (-44.5 to -1.5)	-30.4 (-46.3 to -8.3)
	DALYs	5281.6 (4282 to 6438.8)	4735.4 (3626.5 to 5999.9)	5752.8 (4323.8 to 7374.5)	3408.8 (2866.1 to 3974.5)	3104.9 (2498.2 to 3733.7)	3711.3 (3009 to 4483.2)	-35.5 (-48.4 to -20.5)	-34.4 (-51.2 to -13.4)	-35.5 (-51.1 to -12.2)
	YLLs	5040.2 (4045.9 to 6192.3)	4473.3 (3365.5 to 5745.1)	5530.5 (4151 to 7161.6)	3156.3 (2626.5 to 3692.2)	2847 (2274.8 to 3459.8)	3464.6 (2783.1 to 4207.7)	-37.4 (-50.4 to -22)	-36.4 (-53.2 to -14.7)	-37.4 (-52.9 to -13.7)
	YLDs	241.4 (171.7 to 316.8)	262.2 (183.6 to 349.9)	222.3 (158.4 to 294.7)	252.5 (181.5 to 334)	257.9 (181.6 to 343.9)	246.6 (178.5 to 327.6)	4.6 (-7.3 to 18.2)	-1.6 (-18.6 to 20.6)	11 (-3.4 to 27.3)
Yazd	Deaths	224.4 (176.8 to 274.7)	211.7 (153.1 to 276.2)	232.1 (171.7 to 296.4)	157.9 (127.9 to 189.6)	152.1 (117.2 to 191)	163 (126.1 to 202)	-29.6 (-44.7 to -10.9)	-28.2 (-49 to 0.5)	-29.8 (-47.5 to -4.7)
	DALYs	4250.9 (3347.7 to 5188.5)	3805.4 (2814.1 to 4947.1)	4640 (3383.3 to 6080.2)	2770 (2299.8 to 3278.7)	2475.4 (1989.8 to 3074.2)	3033 (2420.8 to 3728.7)	-34.8 (-49.1 to -17.2)	-34.9 (-52.4 to -9.3)	-34.6 (-52 to -10.2)
	YLLs	4010.8 (3132 to 4935.9)	3553.9 (2591.2 to 4662.4)	4412 (3195.9 to 5837.3)	2513.1 (2057.7 to 2998.9)	2213.5 (1732.3 to 2759)	2781.6 (2169.4 to 3465.7)	-37.3 (-51.4 to -19.2)	-37.7 (-55.5 to -10.9)	-37 (-54.9 to -11.7)
	YLDs	240.1 (170.7 to 316.3)	251.5 (175.6 to 333.3)	228 (161.8 to 303.8)	256.9 (185.8 to 333.5)	262 (191.1 to 342.6)	251.5 (178.8 to 334.4)	7 (-4.9 to 20)	4.2 (-13.3 to 27)	10.3 (-3.1 to 27)

Province	Measure	Attributed age-standardized rate (per 100,000)						% Change (1990 to 2019)		
		1990			2019					
		Both	Female	Male	Both	Female	Male	Both	Female	Male
Zanjan	Deaths	230.9 (184.1 to 282.4)	199.4 (152 to 253)	262.8 (200.4 to 329.5)	171.7 (141.4 to 199)	144.8 (112.1 to 175)	201.2 (163.2 to 238)	-25.7 (-38.9 to -9.9)	-27.4 (-45.2 to -5.3)	-23.5 (-40.7 to -1.8)
	DALYs	4567.9 (3723.1 to 5570.4)	3796.3 (3007 to 4840.7)	5279 (4088.5 to 6728.9)	3141.4 (2670.6 to 3559.5)	2542.9 (2059.6 to 2981.3)	3781.2 (3127.7 to 4441.7)	-31.2 (-43.2 to -16.2)	-33 (-48.8 to -12.8)	-28.4 (-45.3 to -7.3)
	YLLs	4299.8 (3480.5 to 5300.8)	3516.4 (2760.7 to 4540.5)	5023.1 (3856.7 to 6496.5)	2852.8 (2420.9 to 3258.5)	2249.3 (1808 to 2653.8)	3497.7 (2879.3 to 4160.3)	-33.7 (-45.9 to -18.5)	-36 (-51.8 to -15.5)	-30.4 (-47.4 to -8.6)
	YLDs	268 (195 to 352.2)	279.8 (198.3 to 369.4)	256 (183.4 to 342)	288.6 (209.7 to 374.7)	293.6 (212.3 to 381.2)	283.5 (205 to 369.6)	7.7 (-3 to 20.3)	4.9 (-11.2 to 24.6)	10.8 (-2 to 24.5)

*Data in parentheses are 95% Uncertainty Intervals (95% UIs)

Supplementary Table 6

Province	Measure	Attributed age-standardized rate (per 100,000)						% Change (1990 to 2019)		
		1990			2019					
		Both	Female	Male	Both	Female	Male	Both	Female	Male
Alborz	Deaths	82.9 (57.5 to 116.4)	85.2 (56.1 to 127.4)	80 (53.8 to 115.6)	124.1 (89.1 to 176.7)	137.9 (92.4 to 199.4)	118.1 (81.1 to 172.3)	49.8 (16.2 to 98.9)	61.9 (16.1 to 132.5)	47.6 (3.4 to 106.1)
	DALYs	1821.3 (1353.1 to 2357.3)	1812.4 (1296.6 to 2426.8)	1816.7 (1287.3 to 2394.9)	2715.6 (2144.7 to 3445.2)	2761.4 (2117.9 to 3529.8)	2730.5 (2074.4 to 3648.6)	49.1 (20.5 to 91.1)	52.4 (17.6 to 107.9)	50.3 (10.2 to 104.3)
	YLLs	1435.6 (992.6 to 1965.8)	1412.1 (952.1 to 1993.5)	1443.9 (939.1 to 2024.8)	1963.3 (1475 to 2639.2)	1957 (1419.8 to 2679.3)	2027.4 (1453.5 to 2859.6)	36.8 (4.4 to 87.7)	38.6 (0.3 to 110.3)	40.4 (-3.9 to 108.3)
	YLDs	385.6 (271 to 521.7)	400.3 (277.2 to 542.4)	372.8 (263.2 to 504.4)	752.3 (521.8 to 1012.7)	804.4 (560.8 to 1087.5)	703.1 (486.2 to 950)	95.1 (82.2 to 109.1)	100.9 (83.4 to 120.7)	88.6 (71.2 to 105.6)
Ardebil	Deaths	78.4 (55.4 to 113.4)	71.4 (48.8 to 106.3)	84.6 (58.2 to 123.9)	117.7 (81.1 to 172.1)	107 (71.8 to 155)	129.1 (86.8 to 193.5)	50.1 (18.2 to 90)	49.8 (10.8 to 102.4)	52.7 (10.2 to 107.5)
	DALYs	1785 (1366.5 to 2316.9)	1627.6 (1224.9 to 2158.3)	1917.1 (1401.7 to 2585.6)	2536.5 (1959.3 to 3295.3)	2333 (1781.6 to 2976.4)	2751.8 (2061.5 to 3691.3)	42.1 (16.8 to 72.5)	43.3 (12.9 to 82.3)	43.5 (8.5 to 89.8)
	YLLs	1429.7 (1057.8 to 1948.5)	1266.7 (901.3 to 1798.1)	1567.7 (1078.4 to 2230.1)	1881.1 (1370.9 to 2541.7)	1635.9 (1178.2 to 2182.2)	2140.4 (1529.1 to 3035.4)	31.6 (2.3 to 67.5)	29.1 (-4.4 to 75)	36.5 (-2 to 91.3)
	YLDs	355.3 (250.1 to 484.9)	360.9 (254.5 to 486.4)	349.4 (244.9 to 477.7)	655.4 (455.6 to 892.6)	697 (487.5 to 948.9)	611.4 (423.3 to 842.2)	84.5 (72.2 to 98.8)	93.1 (75.5 to 112.4)	75 (59.2 to 92.8)
Bushehr	Deaths	86.9 (60.6 to 127.2)	77.3 (51.3 to 118.4)	96.2 (64.2 to 141.9)	133.6 (94.4 to 185.9)	125.9 (88.7 to 177.6)	141.7 (96.5 to 204.7)	53.9 (19.3 to 95.6)	62.9 (20.1 to 118.5)	47.3 (5.2 to 100.7)
	DALYs	1930.7 (1427.9 to 2602.1)	1714.6 (1252.2 to 2367.8)	2134.1 (1515.6 to 2970.2)	2900.1 (2251.7 to 3742.7)	2787.5 (2168.8 to 3546.6)	3018.4 (2255.1 to 4007.4)	50.2 (20.8 to 85.6)	62.6 (26.4 to 109)	41.4 (6.1 to 87.3)
	YLLs	1544.9 (1064.6 to 2188.4)	1325.2 (917 to 1928)	1751.9 (1163.7 to 2511.8)	2176 (1644.8 to 2921.9)	1997.4 (1507.2 to 2664.8)	2359.2 (1701.9 to 3268.7)	40.8 (8.6 to 82.6)	50.7 (11 to 108.5)	34.7 (-3.7 to 91.7)
	YLDs	385.8 (268.6 to 524.6)	389.4 (268.8 to 529)	382.2 (268.4 to 516.6)	724.1 (504.1 to 982.8)	790 (546.9 to 1070.6)	659.3 (455.9 to 893.1)	87.7 (74.7 to 101.4)	102.9 (85.3 to 122.9)	72.5 (56.8 to 90.1)

Province	Measure	Attributed age-standardized rate (per 100,000)						% Change (1990 to 2019)		
		1990			2019					
		Both	Female	Male	Both	Female	Male	Both	Female	Male
Chahar Mahaal and Bakhtiari	Deaths	66.1 (46.3 to 95.4)	54.3 (34.9 to 83.5)	77.8 (52.5 to 115.8)	79.5 (52.1 to 125.8)	65.5 (39 to 106)	94.3 (60.9 to 148.3)	20.2 (-11.1 to 61.7)	20.6 (-19.7 to 81)	21.1 (-18.5 to 72.6)
	DALYs	1458.1 (1093.9 to 1934.6)	1194.7 (851.8 to 1628.7)	1696.6 (1234.3 to 2334.5)	1749.3 (1310.7 to 2350.3)	1473.8 (1056.6 to 2032.7)	2039.2 (1492.1 to 2785.3)	20 (-4.6 to 50.4)	23.4 (-8.9 to 67)	20.2 (-12.2 to 59.9)
	YLLs	1163.6 (832.2 to 1629.8)	913.4 (610.7 to 1337.6)	1390.7 (964.8 to 2026.4)	1216.3 (840.8 to 1765)	936.8 (600.8 to 1398.7)	1510.6 (1016 to 2275.3)	4.5 (-22.5 to 39.1)	2.6 (-30.9 to 56.1)	8.6 (-26.2 to 54.1)
	YLDs	294.4 (206.9 to 396.1)	281.3 (193.8 to 381.2)	305.9 (213.3 to 411.9)	533 (368.5 to 729.6)	537.1 (366.5 to 737.9)	528.7 (365.6 to 712.2)	81 (69 to 93.8)	91 (74.3 to 109.9)	72.8 (58.6 to 88.4)
East Azarbayejan	Deaths	87.4 (61.9 to 123.8)	80 (53 to 119)	93.9 (64.9 to 134.2)	124.5 (86.4 to 179.4)	123.5 (82.3 to 183.3)	125.5 (85.4 to 186.2)	42.4 (10.1 to 81.3)	54.4 (9.6 to 112.1)	33.6 (-3.1 to 83.2)
	DALYs	1839 (1416.3 to 2400.3)	1642.7 (1213.5 to 2225)	2009.9 (1470.9 to 2746.6)	2617.6 (2045.6 to 3379.1)	2530.6 (1924.2 to 3326.5)	2705.3 (2041.2 to 3609)	42.3 (15 to 75.8)	54.1 (16.8 to 101.3)	34.6 (1 to 79.2)
	YLLs	1490 (1086.9 to 2045.5)	1300.6 (895.2 to 1876)	1655.5 (1151.9 to 2389.8)	1901.6 (1401.7 to 2633.4)	1787.8 (1267.8 to 2508.2)	2016.7 (1449.6 to 2825.1)	27.6 (-2.4 to 66.3)	37.5 (-2.6 to 93.6)	21.8 (-15.7 to 73.7)
	YLDs	348.9 (245.3 to 474.7)	342.1 (240 to 468)	354.4 (248.8 to 477.7)	715.9 (502.3 to 969)	742.8 (520.1 to 998)	688.6 (482.9 to 947.5)	105.2 (90.7 to 120.8)	117.1 (96.6 to 138.9)	94.3 (76 to 113.6)
Fars	Deaths	75.7 (53.2 to 107.9)	68.3 (46.3 to 100.6)	82.4 (54.1 to 117.5)	102.1 (69.4 to 150.8)	108.1 (71 to 164)	97.2 (64.7 to 150.9)	34.8 (1.5 to 81.9)	58.2 (11.1 to 120.3)	17.8 (-19.4 to 79.1)
	DALYs	1630.7 (1202.2 to 2157.7)	1427.6 (1046.9 to 1944.8)	1815 (1275.5 to 2496.5)	2253.9 (1736.6 to 2923.3)	2261.9 (1706.8 to 2965.7)	2253.5 (1685.3 to 3078)	38.2 (9.7 to 77.5)	58.4 (18.9 to 110.8)	24.2 (-10.2 to 78.4)
	YLLs	1341.4 (917.3 to 1852)	1138.1 (773 to 1606.9)	1526.2 (996.9 to 2149.8)	1631.1 (1189 to 2252.6)	1603.2 (1130 to 2257.2)	1666.5 (1150 to 2452.7)	21.6 (-8.8 to 65.8)	40.9 (-1.2 to 102.2)	9.2 (-26.3 to 71.5)
	YLDs	289.3 (203.4 to 388.1)	289.5 (202.7 to 397.8)	288.7 (203.8 to 385.1)	622.9 (434.1 to 843.9)	658.7 (454 to 909.6)	587 (414.3 to 804.8)	115.3 (98.7 to 132.1)	127.5 (105.1 to 152.9)	103.3 (82.3 to 125.2)

Province	Measure	Attributed age-standardized rate (per 100,000)						% Change (1990 to 2019)		
		1990			2019					
		Both	Female	Male	Both	Female	Male	Both	Female	Male
Gilan	Deaths	81 (57.8 to 116.2)	75.3 (50.6 to 112.7)	86.7 (58.7 to 125.2)	125.8 (85.4 to 180)	135.3 (90.6 to 198.5)	117.4 (77.6 to 170.3)	55.3 (20.2 to 101.2)	79.8 (28 to 144.9)	35.4 (-5.2 to 93.1)
	DALYs	1740 (1317.2 to 2303.4)	1585 (1151.4 to 2136.3)	1892.6 (1381.5 to 2593.1)	2742.1 (2106.7 to 3497)	2830.6 (2146.6 to 3655.5)	2659.3 (1943.4 to 3525)	57.6 (27.2 to 96.8)	78.6 (35.8 to 133.6)	40.5 (4.5 to 95.1)
	YLLs	1427.7 (1032.8 to 1963.5)	1270.3 (879 to 1829.2)	1582.7 (1086.7 to 2239.3)	2025.9 (1476.6 to 2703)	2061.6 (1477.8 to 2831.4)	1997.3 (1374.6 to 2788)	41.9 (9 to 87.1)	62.3 (14.9 to 128)	26.2 (-13.7 to 87.8)
	YLDs	312.3 (219.4 to 423.1)	314.8 (218.1 to 429.2)	309.9 (217.4 to 420)	716.2 (499.1 to 961.9)	769 (530.5 to 1048.5)	662.1 (462.8 to 889.9)	129.3 (111.7 to 146.5)	144.3 (121.3 to 169)	113.6 (94.4 to 135.1)
Golestan	Deaths	92 (64.2 to 132.2)	84 (56 to 127)	99.7 (68.3 to 147.2)	144.3 (102.4 to 200.4)	134.6 (92.8 to 189.7)	155.3 (107.3 to 223.9)	56.8 (24.7 to 99.6)	60.2 (17.4 to 118.5)	55.7 (13.9 to 109.8)
	DALYs	2076.6 (1580.5 to 2721.2)	1892.2 (1365.1 to 2546.5)	2243.1 (1627.1 to 3092.6)	3290.1 (2553.9 to 4247.2)	3097.1 (2374.4 to 3961)	3502.2 (2627.1 to 4743.6)	58.4 (29.7 to 96)	63.7 (23.5 to 114.1)	56.1 (17.4 to 106)
	YLLs	1686.5 (1209.6 to 2293.9)	1488.1 (1002.3 to 2118.9)	1867.3 (1283.4 to 2644.8)	2541.2 (1868.5 to 3425.3)	2304 (1685.8 to 3120.9)	2799.8 (1996.5 to 4001.4)	50.7 (19 to 95.2)	54.8 (10.5 to 117.8)	49.9 (6.4 to 108.1)
	YLDs	390.1 (273.1 to 531.7)	404.1 (279.7 to 546)	375.8 (264.1 to 506.6)	748.8 (525.4 to 1007.7)	793 (555.7 to 1066.4)	702.4 (486 to 952.1)	92 (78.4 to 107.8)	96.2 (77.8 to 117.4)	86.9 (71 to 105.5)
Hamadan	Deaths	74.8 (52 to 106.5)	70.3 (47.6 to 106.8)	78.1 (51.7 to 113.9)	95.3 (64.4 to 144.3)	102.7 (65.3 to 159.6)	90.3 (59.4 to 139.4)	27.3 (-4.2 to 67.8)	46.2 (-0.5 to 106.3)	15.6 (-22.1 to 72)
	DALYs	1637.3 (1230.8 to 2117.9)	1467.2 (1073.4 to 1992.9)	1783.4 (1252.4 to 2469.9)	2110 (1586.7 to 2816.3)	2119 (1566.2 to 2866.1)	2116.3 (1550.5 to 2908.4)	28.9 (2.5 to 61.9)	44.4 (7.6 to 89.4)	18.7 (-14.4 to 67.7)
	YLLs	1350.9 (963.6 to 1833.1)	1173.5 (816.7 to 1683.5)	1504.2 (1005.4 to 2167.6)	1552.5 (1103 to 2170.2)	1521 (1048.6 to 2190.4)	1600.1 (1085.3 to 2332.7)	14.9 (-13.2 to 53.5)	29.6 (-11.3 to 84.8)	6.4 (-29 to 60.9)
	YLDs	286.4 (200.9 to 386.2)	293.7 (204.6 to 398.9)	279.2 (195.1 to 379.2)	557.5 (392.2 to 752.1)	597.9 (413.7 to 809.3)	516.2 (360.2 to 698.9)	94.6 (81.3 to 108.5)	103.6 (83.1 to 124.8)	84.9 (68.8 to 102.2)

Province	Measure	Attributed age-standardized rate (per 100,000)						% Change (1990 to 2019)		
		1990			2019					
		Both	Female	Male	Both	Female	Male	Both	Female	Male
Hormozgan	Deaths	84.2 (59.7 to 118.7)	71.4 (50.2 to 101.4)	96.9 (65 to 139.5)	101.8 (71 to 145.8)	103.5 (69.9 to 153.1)	102.8 (68.7 to 151.9)	20.9 (-6.7 to 58.8)	45 (6.3 to 97.5)	6.2 (-26.2 to 51.5)
	DALYs	1899.6 (1406.2 to 2480)	1611.4 (1204.5 to 2123.7)	2154.7 (1496 to 2904.5)	2347 (1808 to 3048.7)	2249.1 (1723.4 to 2960.6)	2470.7 (1810.9 to 3261)	23.6 (-1.2 to 59.5)	39.6 (8.4 to 82.4)	14.7 (-16.9 to 60.1)
	YLLs	1568.9 (1088.5 to 2145.7)	1285.6 (900.9 to 1764.7)	1820.1 (1186 to 2598.4)	1708.1 (1252.2 to 2321.6)	1593.2 (1142.4 to 2226.1)	1848.7 (1262.6 to 2630.6)	8.9 (-16.7 to 49.5)	23.9 (-9.9 to 73.8)	1.6 (-31.3 to 52.4)
	YLDs	330.7 (230.8 to 447.2)	325.8 (227.3 to 440.3)	334.6 (232.2 to 455.6)	638.9 (446.6 to 863.8)	655.9 (451.8 to 890.6)	622 (437.2 to 846.6)	93.2 (80.2 to 108.7)	101.3 (83.6 to 120.5)	85.9 (69.9 to 104.5)
Ilam	Deaths	75.4 (53.4 to 106.4)	63.4 (42.9 to 94.7)	84.7 (57.9 to 122)	111.5 (75.8 to 163)	111.2 (73.2 to 164)	110.8 (74.2 to 166.5)	47.8 (12.5 to 88.8)	75.4 (28.7 to 136.1)	30.8 (-9.8 to 82.9)
	DALYs	1647.8 (1238.8 to 2193.7)	1379.4 (1021.6 to 1880.2)	1849.4 (1341.9 to 2544.6)	2377.6 (1849.2 to 3086.9)	2374.2 (1783.7 to 3101.5)	2368.3 (1783.6 to 3108.5)	44.3 (15 to 78.8)	72.1 (32.2 to 124.5)	28.1 (-6.3 to 69.1)
	YLLs	1357.5 (985.1 to 1859.1)	1094.2 (751.3 to 1594.5)	1555.8 (1085.4 to 2226.3)	1762.5 (1286.3 to 2400.1)	1723.3 (1213 to 2359.8)	1788.3 (1270.4 to 2504.2)	29.8 (-0.6 to 69.6)	57.5 (14.1 to 120.4)	14.9 (-19.7 to 62.5)
	YLDs	290.3 (202.2 to 393.7)	285.2 (199.2 to 392.9)	293.6 (203.2 to 393.8)	615.1 (430.6 to 836)	650.8 (454.4 to 880.5)	580 (407 to 788.4)	111.9 (97.5 to 128.1)	128.2 (107.5 to 153.4)	97.5 (80.9 to 116.2)
Isfahan	Deaths	82.3 (51.9 to 132.1)	81.1 (47.4 to 137.6)	81.4 (49.4 to 134.4)	116.1 (80.9 to 168.9)	118.7 (80.1 to 178)	115.1 (80.1 to 164.3)	41.1 (5.2 to 99.9)	46.5 (0.2 to 125.4)	41.5 (-5.1 to 115.4)
	DALYs	1677.9 (1193.1 to 2317.4)	1569.2 (1083.5 to 2315.1)	1758 (1194.7 to 2538.2)	2534.7 (1975.6 to 3213.8)	2450.2 (1890.8 to 3163.7)	2629.3 (1982.9 to 3428.7)	51.1 (19 to 95.1)	56.1 (15.2 to 113.1)	49.6 (7.1 to 107.4)
	YLLs	1325.3 (869.6 to 1948.6)	1218 (775.6 to 1914.9)	1404.2 (875 to 2152.8)	1837.6 (1376.7 to 2451.1)	1727.6 (1248.6 to 2403.1)	1956.7 (1397.4 to 2686.7)	38.7 (3.9 to 92)	41.8 (-2.4 to 113.5)	39.3 (-6.4 to 113)
	YLDs	352.6 (246.9 to 482.3)	351.2 (245.8 to 483.3)	353.9 (243.8 to 480.9)	697.2 (489.2 to 947.9)	722.6 (506 to 979.4)	672.6 (468.7 to 910)	97.7 (83.6 to 114.7)	105.8 (88.4 to 125.2)	90.1 (70.7 to 113.2)

Province	Measure	Attributed age-standardized rate (per 100,000)						% Change (1990 to 2019)		
		1990			2019					
		Both	Female	Male	Both	Female	Male	Both	Female	Male
Kerman	Deaths	85.1 (61.3 to 119.6)	74.5 (50.3 to 111.8)	95.4 (65.7 to 136.6)	102.6 (70.7 to 153.1)	106.3 (69.5 to 163.9)	99.3 (64.9 to 150.8)	20.6 (-5.9 to 55.2)	42.7 (-0.1 to 91.8)	4 (-25.4 to 46.2)
	DALYs	1872.9 (1417.2 to 2477.8)	1601.3 (1178.4 to 2193.6)	2121.4 (1551.8 to 2851.2)	2212 (1719.4 to 2863.4)	2203.9 (1693.7 to 2876.7)	2222.7 (1674.6 to 3019.8)	18.1 (-4.3 to 46.2)	37.6 (3.1 to 74.4)	4.8 (-22 to 39.8)
	YLLs	1552 (1150.3 to 2131.8)	1283.6 (907.2 to 1851.4)	1797.7 (1233.2 to 2511.3)	1584.2 (1162.7 to 2200.4)	1547.3 (1096.5 to 2175.4)	1623.3 (1161.9 to 2334.1)	2.1 (-21.1 to 33.2)	20.5 (-16 to 63.2)	-9.7 (-36.2 to 29)
	YLDs	320.9 (222.8 to 430)	317.7 (220.9 to 429.2)	323.7 (225.1 to 437.3)	627.8 (443.5 to 859.9)	656.6 (460.3 to 904.2)	599.4 (413.3 to 809.9)	95.6 (81.4 to 111.2)	106.7 (87.4 to 127.5)	85.2 (69 to 104.7)
Kermanshah	Deaths	84.6 (60.4 to 118)	72.3 (49.4 to 105.6)	94.7 (65.1 to 135.2)	107.3 (70.7 to 157.1)	115.4 (72.6 to 171.5)	100.8 (64.8 to 152.4)	26.7 (-3.6 to 64.1)	59.6 (15.4 to 116.5)	6.4 (-27.3 to 50.3)
	DALYs	1899.5 (1415.4 to 2457.8)	1592.8 (1172 to 2121.7)	2149.1 (1531 to 2899)	2409.6 (1806.8 to 3200)	2471.6 (1809.3 to 3300.4)	2360.4 (1710 to 3261.2)	26.9 (-0.1 to 58.2)	55.2 (16 to 101.1)	9.8 (-20.2 to 48.3)
	YLLs	1585.7 (1125.4 to 2106.9)	1277.2 (891 to 1789.2)	1837.4 (1248.7 to 2600.9)	1774.7 (1263.9 to 2505.9)	1798.2 (1238.4 to 2580.9)	1765 (1208.8 to 2602.8)	11.9 (-15.9 to 46.2)	40.8 (-0.7 to 95.4)	-3.9 (-34.9 to 37.8)
	YLDs	313.8 (221.8 to 417.7)	315.6 (218 to 425.7)	311.6 (221.1 to 416.6)	634.9 (442.5 to 856.2)	673.4 (467.7 to 917.2)	595.4 (410.2 to 803.4)	102.3 (88.3 to 118.2)	113.4 (92.6 to 137.3)	91 (74.7 to 110)
Khorasan-e-Razavi	Deaths	140.8 (93 to 219.3)	117.1 (74.2 to 194.6)	163.1 (103.1 to 256.5)	122.4 (86.4 to 175.9)	115.7 (80 to 170.5)	129.4 (89.6 to 185.5)	-13.1 (-30.4 to 13.2)	-1.2 (-26.8 to 40.9)	-20.6 (-41.5 to 9.8)
	DALYs	2881.6 (2102.9 to 4029.6)	2324.6 (1658.9 to 3327.4)	3382.7 (2347.4 to 4849.2)	2992.1 (2348.9 to 3858)	2703.8 (2109.6 to 3484.1)	3288.4 (2551 to 4297.2)	3.8 (-16.6 to 28.6)	16.3 (-11.7 to 54.2)	-2.8 (-27.4 to 29.7)
	YLLs	2366.5 (1638.5 to 3492.7)	1856.4 (1226 to 2847.4)	2824.3 (1839.2 to 4247.5)	2076.6 (1571.5 to 2889.8)	1833.4 (1352.4 to 2538.7)	2326.8 (1688 to 3261.7)	-12.2 (-30.9 to 13.8)	-1.2 (-29.2 to 40.8)	-17.6 (-41.5 to 18.6)
	YLDs	515.1 (359.1 to 691.9)	468.2 (321.1 to 635.7)	558.4 (387.3 to 746.1)	915.5 (639.6 to 1228.2)	870.4 (616.4 to 1169.4)	961.5 (662.1 to 1309.1)	77.7 (64.5 to 92.9)	85.9 (69.4 to 104.8)	72.2 (55.2 to 91.1)

Province	Measure	Attributed age-standardized rate (per 100,000)						% Change (1990 to 2019)		
		1990			2019					
		Both	Female	Male	Both	Female	Male	Both	Female	Male
Khuzestan	Deaths	100.9 (71 to 144.1)	93.9 (63.7 to 141.1)	107.3 (72.7 to 154.9)	164.3 (121 to 222.4)	162.2 (113.4 to 223.8)	166.9 (119.7 to 230.1)	62.8 (28 to 107.6)	72.8 (26.2 to 140.6)	55.5 (14 to 114.1)
	DALYs	2260.3 (1730.4 to 2920.1)	2071.1 (1552.6 to 2771.2)	2430.4 (1777.2 to 3278.2)	3759.4 (2970.9 to 4653.9)	3599.2 (2832.7 to 4516.1)	3922.5 (3080.1 to 5003.3)	66.3 (37.5 to 103.2)	73.8 (37.4 to 126.3)	61.4 (24.1 to 113.3)
	YLLs	1844.5 (1348.3 to 2462.6)	1655.4 (1188.8 to 2282.8)	2014.4 (1405.7 to 2843.7)	2850.5 (2168 to 3693.5)	2667.3 (1974.5 to 3460.9)	3036.5 (2221.3 to 4109.6)	54.5 (21.5 to 97.8)	61.1 (19.4 to 122.6)	50.7 (10.3 to 112.5)
	YLDs	415.8 (291.7 to 564.4)	415.7 (289.2 to 566.3)	416 (288.5 to 565.8)	908.9 (632.8 to 1223.8)	931.9 (654.4 to 1256.5)	886 (607.2 to 1197.5)	118.6 (103.6 to 134.1)	124.2 (107.8 to 143)	113 (93 to 136)
Kohgiluyeh and Boyer-Ahmad	Deaths	67.3 (46.6 to 98)	64.2 (42.5 to 98.9)	69.3 (46.1 to 100.8)	82.3 (53.4 to 127.1)	94.8 (57.2 to 153.3)	73.9 (46.9 to 119.1)	22.4 (-10.6 to 66.5)	47.6 (-3.5 to 116.6)	6.6 (-30.6 to 60.1)
	DALYs	1473.9 (1103.6 to 1961.4)	1355.1 (986.6 to 1852.1)	1566.7 (1144.3 to 2137.4)	1797.9 (1329.9 to 2404.2)	1924.6 (1354.9 to 2650.8)	1713.2 (1259 to 2348.4)	22 (-4.5 to 54.4)	42 (3.7 to 92.4)	9.4 (-20.1 to 53)
	YLLs	1168.8 (833.9 to 1611.6)	1055.5 (715.2 to 1516.9)	1256.2 (849.1 to 1815.3)	1261.2 (873.2 to 1811.4)	1362.4 (878.2 to 2041.4)	1199.4 (808.6 to 1793.6)	7.9 (-22.4 to 47.6)	29.1 (-15.2 to 92.1)	-4.5 (-36.6 to 45.4)
	YLDs	305.1 (212.5 to 413.9)	299.6 (205.6 to 407.6)	310.5 (216.4 to 420.8)	536.6 (372 to 727.6)	562.2 (389.2 to 768.6)	513.8 (356.5 to 694.5)	75.9 (64.5 to 87.1)	87.7 (71.8 to 103.5)	65.5 (51.7 to 80.9)
Kurdistan	Deaths	77.6 (55.8 to 107.7)	65.1 (44.9 to 95.1)	88.1 (60.8 to 123.4)	90.1 (62.3 to 131.6)	96.4 (63.7 to 143.2)	85.5 (56.6 to 129.3)	16.1 (-9.8 to 48.2)	48 (4.9 to 106.9)	-3 (-32.2 to 38.6)
	DALYs	1729.8 (1333.8 to 2255.5)	1452.5 (1083.6 to 1964)	1957.7 (1424.2 to 2604.2)	2057.3 (1599.2 to 2726)	2085.2 (1560.7 to 2754.6)	2045.7 (1500 to 2737.5)	18.9 (-3.4 to 46.3)	43.6 (9.6 to 87.3)	4.5 (-21.5 to 41)
	YLLs	1428.6 (1035.4 to 1915.1)	1157.1 (820.5 to 1649.4)	1652.3 (1135 to 2263)	1463.1 (1076.8 to 2002.6)	1457 (1014.9 to 2058.7)	1485.1 (1004.4 to 2113)	2.4 (-20.6 to 32.6)	25.9 (-10.2 to 75.7)	-10.1 (-37.4 to 30.1)
	YLDs	301.3 (211.5 to 409)	295.4 (205.2 to 399.4)	305.4 (212.6 to 416.3)	594.3 (413.3 to 814.9)	628.1 (433.5 to 867.8)	560.6 (393.7 to 764.7)	97.3 (82.4 to 113.6)	112.7 (93 to 137.9)	83.5 (65.2 to 101)

Province	Measure	Attributed age-standardized rate (per 100,000)						% Change (1990 to 2019)		
		1990			2019					
		Both	Female	Male	Both	Female	Male	Both	Female	Male
Lorestan	Deaths	82.4 (59.8 to 114.7)	70.3 (48.5 to 102.8)	93.1 (63.8 to 133.9)	102.5 (69 to 153.6)	86.5 (55.3 to 131.5)	119 (79.1 to 175.8)	24.4 (-4.4 to 60.6)	23.1 (-13.6 to 71.2)	27.9 (-8.2 to 80.6)
	DALYs	1849.9 (1422 to 2403.3)	1571 (1160.4 to 2071.9)	2084 (1531 to 2852.7)	2239.4 (1689.7 to 2960.2)	1926.2 (1409.2 to 2581.8)	2570 (1882.8 to 3467.5)	21.1 (-2.4 to 51.1)	22.6 (-7.7 to 58.9)	23.3 (-7.7 to 68.9)
	YLLs	1506.6 (1111 to 2045.7)	1231.4 (859.9 to 1705.1)	1738.2 (1207.1 to 2504.2)	1647.4 (1169.1 to 2320.5)	1315.7 (870.1 to 1906.5)	1997.6 (1376.8 to 2839.8)	9.3 (-16.9 to 43.6)	6.8 (-25.9 to 50.1)	14.9 (-19.1 to 67.6)
	YLDs	343.2 (239 to 465.9)	339.6 (235.3 to 459.5)	345.8 (240.2 to 467.8)	591.9 (410 to 801.7)	610.5 (421.3 to 840.8)	572.4 (396.8 to 779.1)	72.5 (60.3 to 85.1)	79.8 (64.3 to 96.7)	65.5 (51.3 to 82.4)
Markazi	Deaths	76 (50.2 to 121.8)	72.2 (43.1 to 119.8)	78.9 (52 to 123.2)	91.1 (62.9 to 135.1)	91.4 (61.9 to 138.1)	91.5 (60.7 to 136.3)	19.9 (-10.6 to 61.6)	26.6 (-12.2 to 87.3)	16 (-21.1 to 67)
	DALYs	1635.4 (1211.6 to 2205.4)	1490.3 (1038.9 to 2108.6)	1764.9 (1270.7 to 2446.7)	2161.8 (1678.4 to 2805.2)	2090 (1630.3 to 2722.2)	2237.7 (1652.5 to 3000.8)	32.2 (4.9 to 64.5)	40.2 (3.7 to 88.7)	26.8 (-7.7 to 69.5)
	YLLs	1309 (941 to 1872.5)	1164.9 (743.8 to 1739.3)	1438.1 (983 to 2086.7)	1500.5 (1110.5 to 2084.5)	1400.7 (1014.3 to 1990.5)	1604.8 (1118.1 to 2304.1)	14.6 (-13.6 to 51.9)	20.2 (-17.4 to 75.8)	11.6 (-23.4 to 61)
	YLDs	326.5 (229.9 to 441)	325.5 (225.4 to 442.6)	326.8 (230.4 to 444.8)	661.4 (465.1 to 894.6)	689.3 (487.7 to 929.2)	632.9 (442.7 to 860.3)	102.6 (87.1 to 118.2)	111.8 (91.8 to 133.4)	93.6 (75.5 to 112.9)
Mazandaran	Deaths	92.4 (63.8 to 136.7)	94.3 (61.8 to 148.6)	88.7 (59.1 to 133.8)	120.1 (86.4 to 165.1)	123.2 (86.9 to 170.9)	116.9 (81.3 to 167.1)	29.9 (0.6 to 66.5)	30.7 (-7 to 82.6)	31.8 (-6.4 to 84.3)
	DALYs	2031.8 (1561.2 to 2653.2)	2026.1 (1497.8 to 2738.3)	2017.9 (1477.1 to 2775.1)	2807.9 (2238.9 to 3467.9)	2840.9 (2222.2 to 3519.1)	2772.7 (2153.9 to 3613.4)	38.2 (13.2 to 67.1)	40.2 (7.9 to 81.4)	37.4 (3.3 to 79.7)
	YLLs	1599 (1167 to 2164.8)	1580.4 (1088 to 2242.7)	1598 (1097.7 to 2288)	2027.7 (1546 to 2613)	2016.3 (1477.6 to 2638.2)	2038.1 (1482.5 to 2824.7)	26.8 (-1.1 to 61.7)	27.6 (-7.2 to 76.7)	27.5 (-10.5 to 81.3)
	YLDs	432.8 (300 to 581.7)	445.8 (309.9 to 600.9)	419.9 (293.4 to 564.4)	780.3 (543.6 to 1037.9)	824.6 (576.1 to 1096.4)	734.6 (507.9 to 991.9)	80.3 (67.3 to 94)	85 (68.3 to 104.6)	75 (58.9 to 92.5)

Province	Measure	Attributed age-standardized rate (per 100,000)						% Change (1990 to 2019)		
		1990			2019					
		Both	Female	Male	Both	Female	Male	Both	Female	Male
North Khorasan	Deaths	126.9 (85.7 to 191.3)	108.9 (71 to 173.8)	143.2 (92.7 to 221.1)	115.6 (81.1 to 167.6)	120.3 (81.2 to 180)	113.1 (78.6 to 160.3)	-8.9 (-27.4 to 16.5)	10.5 (-18.5 to 54.4)	-21 (-40.6 to 8.1)
	DALYs	2859.2 (2118.1 to 3920.7)	2403.9 (1747.6 to 3369.1)	3256.7 (2314.1 to 4596.6)	2850.4 (2228.8 to 3683.2)	2809.6 (2152.6 to 3662.3)	2913.6 (2238.3 to 3788.6)	-0.3 (-19.1 to 21.1)	16.9 (-10 to 50)	-10.5 (-31.8 to 18)
	YLLs	2270.1 (1596.1 to 3316.7)	1857.5 (1254.9 to 2788.5)	2628.8 (1729.8 to 3854.4)	1985.1 (1462.4 to 2715.5)	1951.4 (1409.9 to 2753)	2041.1 (1462.5 to 2843.5)	-12.6 (-31.2 to 11.5)	5.1 (-24.1 to 45.4)	-22.4 (-44.1 to 9.1)
	YLDs	589.1 (409.9 to 803.8)	546.4 (380.2 to 743.7)	627.9 (436.2 to 853.9)	865.4 (601.9 to 1179.1)	858.2 (598.2 to 1180.7)	872.4 (600.9 to 1192.8)	46.9 (37.4 to 57.2)	57.1 (43.8 to 71.5)	38.9 (27.8 to 51.3)
Qazvin	Deaths	64.7 (47.3 to 89.9)	58.4 (40.2 to 85.9)	71.6 (49.9 to 104.1)	94.1 (65.1 to 137.1)	85.3 (56 to 127.9)	103.4 (67.4 to 153.8)	45.5 (11.3 to 90)	46 (0.7 to 102.5)	44.4 (0.3 to 101.9)
	DALYs	1471.9 (1123.7 to 1914.3)	1336.9 (999.6 to 1789.4)	1603.1 (1173.4 to 2172.6)	2099.9 (1616.3 to 2698.8)	1935 (1440.2 to 2512.3)	2271.2 (1683.1 to 3028.3)	42.7 (14.6 to 77.2)	44.7 (8.3 to 88.6)	41.7 (5.7 to 87.7)
	YLLs	1162.5 (848.7 to 1567.1)	1021.2 (724.1 to 1450.3)	1300.2 (900.6 to 1856.2)	1488.6 (1069.6 to 2040.2)	1284.2 (891.4 to 1795.8)	1701.4 (1169.9 to 2438.1)	28 (-2 to 69.8)	25.8 (-12.9 to 78.6)	30.9 (-9.7 to 85.6)
	YLDs	309.4 (215.6 to 411.7)	315.6 (219.8 to 424.5)	303 (211.2 to 406.2)	611.3 (431.3 to 822)	650.8 (458.9 to 888)	569.8 (397.7 to 762.2)	97.6 (84.5 to 112.2)	106.2 (87.4 to 125.4)	88.1 (72.2 to 105.9)
Qom	Deaths	88.4 (63.8 to 124.3)	89.4 (61.9 to 133.5)	86.1 (59.1 to 121)	105 (72.8 to 148.7)	125.6 (85 to 183)	92.2 (61.9 to 134)	18.8 (-7.2 to 60)	40.5 (2.8 to 94.7)	7 (-24 to 52.8)
	DALYs	1945.9 (1454.8 to 2478.4)	1886.2 (1380.5 to 2522.9)	1985.6 (1432.4 to 2622)	2323.3 (1814.9 to 2920.3)	2546.1 (1980.3 to 3282)	2160.1 (1614.3 to 2868.5)	19.4 (-2.7 to 53)	35 (6.2 to 74.6)	8.8 (-18.6 to 48.2)
	YLLs	1552.3 (1117.4 to 2070.2)	1485.5 (1028.9 to 2054.4)	1598.6 (1108.8 to 2188.2)	1648 (1221.2 to 2215.3)	1818.6 (1324.5 to 2488.6)	1533 (1067.2 to 2162.2)	6.2 (-18.1 to 46.3)	22.4 (-8.7 to 71)	-4.1 (-32.7 to 44.2)
	YLDs	393.6 (275.4 to 530.2)	400.7 (280.2 to 544.6)	387 (270.1 to 516.9)	675.3 (473.2 to 917.5)	727.5 (506.9 to 978)	627.1 (433 to 850.3)	71.6 (59.2 to 83.4)	81.6 (65.4 to 98.1)	62 (47.6 to 77.8)

Province	Measure	Attributed age-standardized rate (per 100,000)						% Change (1990 to 2019)		
		1990			2019					
		Both	Female	Male	Both	Female	Male	Both	Female	Male
Semnan	Deaths	89.6 (63.8 to 129.8)	79.4 (52.8 to 122.3)	100.8 (70.3 to 148.2)	119.4 (84.9 to 169)	103.5 (71.8 to 145.4)	136.1 (93.7 to 194)	33.3 (4.6 to 73)	30.5 (-7.9 to 81.3)	35.1 (-1.7 to 83.3)
	DALYs	2018 (1548.2 to 2634.2)	1780.7 (1327.8 to 2450)	2257.7 (1680.6 to 3058.1)	2727.8 (2127.9 to 3479.2)	2461.2 (1920.8 to 3094.8)	3004.9 (2270.6 to 3993.6)	35.2 (11.5 to 67.7)	38.2 (5.4 to 76.7)	33.1 (2.4 to 71.1)
	YLLs	1587.1 (1150.2 to 2179.8)	1341.3 (927.8 to 1953)	1835.9 (1287.4 to 2600.9)	1961.1 (1437.5 to 2626.2)	1629.4 (1180.7 to 2187.2)	2305.3 (1642 to 3200.6)	23.6 (-3.7 to 62.1)	21.5 (-14.3 to 69.1)	25.6 (-9 to 71.6)
	YLDs	430.9 (302.8 to 579.1)	439.4 (304.4 to 598.8)	421.8 (295.6 to 563.7)	766.7 (542 to 1033.6)	831.8 (581.7 to 1121.2)	699.6 (490.4 to 941.2)	77.9 (67.1 to 90)	89.3 (74.4 to 105.1)	65.9 (51.4 to 81.9)
Sistan and Baluchistan	Deaths	67.8 (47.8 to 96.1)	56.4 (38.8 to 83.5)	77.7 (51.2 to 116.5)	94.5 (67.7 to 135.6)	88.3 (61.3 to 127.7)	100.8 (68.5 to 150.7)	39.5 (7.5 to 82.7)	56.5 (12.1 to 123.8)	29.8 (-9.2 to 86.4)
	DALYs	1588.6 (1127 to 2091.8)	1323.4 (960.5 to 1791.1)	1800.4 (1185.8 to 2483.7)	2307.4 (1779.8 to 3050.9)	2161.7 (1641.6 to 2862.3)	2455.2 (1843.7 to 3325.5)	45.2 (16 to 86.8)	63.3 (23.2 to 121.8)	36.4 (0.6 to 94.8)
	YLLs	1276.3 (879.8 to 1748.7)	1027.1 (708.1 to 1451)	1476 (905.3 to 2133.9)	1749.4 (1311.9 to 2412.4)	1610.6 (1162.9 to 2242.6)	1890.5 (1345.3 to 2765.6)	37.1 (4.3 to 89.7)	56.8 (9.8 to 132.4)	28.1 (-11.7 to 98.1)
	YLDs	312.3 (216.6 to 422.3)	296.3 (207.6 to 403.7)	324.4 (224.6 to 437.7)	558 (385.3 to 759.3)	551.1 (382.4 to 748.1)	564.7 (395.6 to 771.4)	78.6 (66.4 to 91.4)	86 (68.4 to 104.1)	74.1 (59.6 to 90.8)
South Khorasan	Deaths	112.7 (73.1 to 165.7)	101.3 (65.4 to 156)	123.4 (78.2 to 185.9)	96.4 (65.9 to 138.5)	89.1 (60.2 to 129)	105.7 (69.1 to 156)	-14.4 (-32.4 to 9.5)	-12 (-34.2 to 20.9)	-14.4 (-35.9 to 21.8)
	DALYs	2565.5 (1902.4 to 3431.3)	2342 (1708.9 to 3188.5)	2764.1 (1969.5 to 3810.6)	2543.9 (1983.6 to 3214.9)	2488.5 (1918.6 to 3152.1)	2615.7 (1982.7 to 3400.3)	-0.8 (-18.9 to 20)	6.3 (-15.8 to 35.2)	-5.4 (-26.9 to 23.7)
	YLLs	1919.8 (1330.2 to 2699.2)	1678.5 (1137.5 to 2514.9)	2136.1 (1414.8 to 3144.8)	1558.6 (1148.7 to 2159.3)	1413.4 (1011.9 to 1908.6)	1724.7 (1211.3 to 2435)	-18.8 (-36.6 to 5)	-15.8 (-38.3 to 17.5)	-19.3 (-41.4 to 15.7)
	YLDs	645.7 (447.4 to 876.9)	663.6 (450.6 to 907.9)	628 (436.4 to 856.8)	985.3 (687.9 to 1334.1)	1075.1 (749.8 to 1461.1)	891 (617.9 to 1192.3)	52.6 (43.8 to 63.6)	62 (50.1 to 76.1)	41.9 (29.8 to 55)

Province	Measure	Attributed age-standardized rate (per 100,000)						% Change (1990 to 2019)		
		1990			2019					
		Both	Female	Male	Both	Female	Male	Both	Female	Male
Tehran	Deaths	105.1 (76.6 to 144.9)	110.7 (74.7 to 157.9)	97.5 (67.3 to 141.4)	90.5 (69 to 115.9)	102.9 (73.9 to 138)	80 (59.1 to 106)	-13.9 (-32.1 to 11.5)	-7.1 (-30.8 to 31.4)	-17.9 (-42.1 to 16.6)
	DALYs	2316.2 (1810.9 to 2942.8)	2328 (1743.5 to 3039.9)	2277.9 (1657.1 to 3091)	2187.6 (1768.2 to 2626.6)	2307.1 (1811.7 to 2835.7)	2082.8 (1640.5 to 2593.4)	-5.6 (-21.2 to 15.7)	-0.9 (-21.7 to 28.2)	-8.6 (-30.5 to 20.3)
	YLLs	1789.1 (1367.8 to 2363.2)	1805.3 (1277.4 to 2457.7)	1744.6 (1233.1 to 2485)	1446.1 (1146.7 to 1771.9)	1541.5 (1140.4 to 1964.2)	1365.1 (1027 to 1778.5)	-19.2 (-36.7 to 4.7)	-14.6 (-37.2 to 19.6)	-21.8 (-46 to 13.2)
	YLDs	527.1 (367.3 to 712.2)	522.7 (363 to 708.5)	533.3 (368.9 to 723.2)	741.4 (516.4 to 989.9)	765.6 (530.6 to 1028.6)	717.7 (500.1 to 965.2)	40.7 (31.6 to 49.8)	46.5 (34.5 to 58.6)	34.6 (22.2 to 48.6)
West Azarbayejan	Deaths	85.3 (59.6 to 123.7)	77.4 (50.2 to 118.8)	92.6 (61.2 to 133.6)	131.6 (88 to 196.3)	136.7 (90 to 206.7)	126.4 (84.1 to 189.6)	54.3 (20.2 to 95.3)	76.5 (29.4 to 141.5)	36.5 (-3.6 to 89.8)
	DALYs	1770.9 (1354.3 to 2333.2)	1575.8 (1153 to 2142.6)	1944.8 (1414.6 to 2628.2)	2637.3 (2016.8 to 3454.9)	2662.2 (2002.3 to 3522.9)	2607.7 (1948.4 to 3464.5)	48.9 (20.1 to 82.4)	68.9 (29.9 to 117.8)	34.1 (-0.4 to 78.4)
	YLLs	1453.4 (1069.3 to 1992.7)	1257.8 (854.9 to 1815.8)	1628.5 (1113.1 to 2319.8)	1958.5 (1399.3 to 2740.2)	1935.2 (1374 to 2746.5)	1979.4 (1390.4 to 2828.3)	34.8 (4.8 to 74.3)	53.9 (12.2 to 113.1)	21.6 (-14.7 to 73.8)
	YLDs	317.5 (221.9 to 430.6)	318.1 (218.8 to 432.5)	316.3 (219.7 to 433.6)	678.8 (468.6 to 914.7)	727.1 (506.9 to 985.3)	628.3 (423.5 to 844.6)	113.8 (98.6 to 131.8)	128.6 (107.4 to 153.9)	98.6 (80.8 to 118.3)
Yazd	Deaths	84.7 (61.1 to 122.4)	75 (51.1 to 109.9)	96.6 (65.9 to 142.3)	126.4 (92.1 to 175.2)	117 (82.8 to 164.3)	136.4 (95.4 to 195.7)	49.2 (17.4 to 91.8)	56.1 (12.7 to 115.6)	41.2 (1.3 to 98.1)
	DALYs	1866.9 (1453.7 to 2411)	1628.6 (1208.3 to 2153.3)	2128.3 (1574.7 to 2831.6)	2871.3 (2277.6 to 3600.3)	2602.2 (2028.6 to 3240.3)	3127.7 (2424.4 to 4111.3)	53.8 (27.1 to 89.5)	59.8 (23.9 to 104.6)	47 (11.8 to 95.2)
	YLLs	1483 (1088.9 to 1991.3)	1260.8 (877.6 to 1758.5)	1725 (1186.4 to 2408.3)	2030.3 (1543.2 to 2693.1)	1767.7 (1300.2 to 2342.9)	2280.8 (1647.2 to 3113.7)	36.9 (7.9 to 80.6)	40.2 (2.1 to 95.1)	32.2 (-5.9 to 90.2)
	YLDs	384 (268 to 512.6)	367.8 (257.2 to 495)	403.3 (279.9 to 553)	841 (590.1 to 1143.4)	834.5 (580.8 to 1123.5)	846.9 (588.7 to 1153.3)	119 (104.6 to 134.6)	126.9 (108.5 to 145.8)	110 (92.4 to 130.6)

Province	Measure	Attributed age-standardized rate (per 100,000)						% Change (1990 to 2019)		
		1990			2019					
		Both	Female	Male	Both	Female	Male	Both	Female	Male
Zanjan	Deaths	58.9 (41 to 83.9)	49.3 (32.9 to 74.4)	69.7 (45.7 to 103.1)	76.2 (51.7 to 114.3)	70.5 (43.4 to 107.3)	82.4 (54.1 to 126.1)	29.4 (-3.1 to 68.2)	42.9 (-2.7 to 100.9)	18.3 (-15.4 to 69.9)
	DALYs	1330.5 (1009.4 to 1779.5)	1118 (816.6 to 1526.4)	1536.3 (1102.3 to 2095.7)	1698.5 (1276.1 to 2258.2)	1586.8 (1167.2 to 2110.4)	1818.5 (1354.5 to 2470.7)	27.7 (1.8 to 56.9)	41.9 (5.6 to 82.7)	18.4 (-10 to 57.7)
	YLLs	1045.7 (754.3 to 1453)	834.9 (564.4 to 1198.4)	1250.4 (833.5 to 1791)	1169.4 (832.3 to 1669.9)	1033.4 (688 to 1470.7)	1315.4 (902 to 1889.8)	11.8 (-15.5 to 46.2)	23.8 (-15.1 to 75.8)	5.2 (-24.6 to 51.7)
	YLDs	284.9 (199.6 to 384.5)	283.2 (196.7 to 383)	285.9 (198 to 387.2)	529.1 (363.5 to 718.4)	553.4 (378.8 to 752.5)	503.1 (349.3 to 682.2)	85.7 (73.7 to 98.8)	95.4 (77.7 to 117.3)	76 (61.3 to 90.9)

*Data in parentheses are 95% Uncertainty Intervals (95% UIs)

Supplementary Table 7

Province	Measure	Attributed age-standardized rate (per 100,000)						% Change (1990 to 2019)		
		1990			2019					
		Both	Female	Male	Both	Female	Male	Both	Female	Male
Alborz	Deaths	87.5 (51.5 to 131.2)	98.4 (56.8 to 150.8)	76.2 (39 to 124.9)	92.9 (62.2 to 128.2)	102.5 (68.9 to 140.9)	89 (54.1 to 130.5)	6.2 (-19.3 to 56.5)	4.2 (-25.8 to 65.6)	16.9 (-19.7 to 94.1)
	DALYs	2356.8 (1411.7 to 3377.6)	2603.1 (1606.7 to 3781.9)	2115.3 (1124.1 to 3384.1)	2593.6 (1823.4 to 3446.1)	2569.1 (1847.4 to 3397.6)	2661.4 (1716.7 to 3662.2)	10 (-14.8 to 58.4)	-1.3 (-25.2 to 45.1)	25.8 (-11.4 to 107.2)
	YLLs	1938.7 (1122.3 to 2879.8)	2088.3 (1173.1 to 3128.7)	1783.2 (912.1 to 2925.7)	1846.6 (1279.1 to 2493.7)	1729.8 (1204.7 to 2336.5)	2001 (1238.3 to 2875.7)	-4.8 (-28.3 to 47)	-17.2 (-39.9 to 32.7)	12.2 (-25 to 92.5)
	YLDs	418.1 (246.3 to 634.6)	514.7 (313.2 to 762.4)	332.1 (176.3 to 535.7)	747 (476.8 to 1062.4)	839.3 (547.1 to 1187.4)	660.4 (408.6 to 964.8)	78.7 (53.8 to 119.1)	63 (41.7 to 96.6)	98.9 (59.7 to 172.8)
Ardebil	Deaths	86.8 (48.1 to 132.7)	96.6 (54.1 to 143.5)	78.1 (36.5 to 132.1)	107.1 (70.1 to 146.7)	98.6 (66.8 to 133)	115.5 (69.6 to 165.4)	23.3 (-3.2 to 76.2)	2 (-25.2 to 50.6)	47.9 (7.4 to 147.1)
	DALYs	2368.8 (1385.3 to 3525)	2624.1 (1561.3 to 3766.8)	2153.6 (1066.7 to 3541.2)	2867.3 (1986.7 to 3805.6)	2614.5 (1884.6 to 3438.4)	3121.1 (2019.5 to 4287.2)	21 (-3.5 to 69.1)	-0.4 (-23.9 to 40)	44.9 (5.2 to 140.9)
	YLLs	2012.8 (1157.1 to 3071.4)	2176 (1252.8 to 3188.6)	1879.3 (918.5 to 3121.5)	2187.2 (1469.2 to 2945.4)	1853 (1295.8 to 2478.7)	2525.5 (1602.2 to 3516.2)	8.7 (-15.4 to 55.1)	-14.8 (-36.6 to 23.7)	34.4 (-4.5 to 127.8)
	YLDs	356 (196.3 to 552.1)	448.1 (262.1 to 665)	274.2 (133.4 to 449.5)	680.1 (434.9 to 983.4)	761.5 (496.6 to 1083)	595.6 (370.2 to 876.3)	91 (63.2 to 148.9)	70 (45.8 to 115.3)	117.2 (75.1 to 251.4)
Bushehr	Deaths	89.8 (51.6 to 137.4)	94.2 (54.9 to 140.8)	85 (42.8 to 139.9)	104.7 (69.8 to 140.9)	100.7 (70.2 to 134)	108.3 (68.3 to 154.8)	16.6 (-9.6 to 67.8)	6.9 (-20 to 56.4)	27.4 (-9.1 to 109.2)
	DALYs	2467.1 (1471.5 to 3568.3)	2589.7 (1628.8 to 3726.2)	2345.6 (1223.7 to 3764.3)	2851.4 (2016.3 to 3758.2)	2736.7 (2005.7 to 3527.6)	2953.7 (1972.9 to 4035)	15.6 (-9.4 to 62.3)	5.7 (-17.6 to 46.2)	25.9 (-11.2 to 101.2)
	YLLs	2075.3 (1206.4 to 3096.9)	2116.3 (1275.1 to 3115.3)	2031.4 (1030 to 3303.7)	2120.2 (1489.7 to 2808.1)	1910.1 (1379.6 to 2472)	2315.3 (1516.1 to 3230.3)	2.2 (-21.5 to 47.5)	-9.7 (-32.5 to 30.9)	14 (-20.8 to 90.5)
	YLDs	391.8 (226.5 to 594)	473.4 (285.8 to 700.1)	314.2 (163.2 to 505.7)	731.2 (470.2 to 1040.4)	826.6 (535 to 1165.1)	638.4 (395.4 to 927.9)	86.6 (59 to 136.9)	74.6 (50.1 to 117.7)	103.2 (61.9 to 195.5)

Province	Measure	Attributed age-standardized rate (per 100,000)						% Change (1990 to 2019)		
		1990			2019					
		Both	Female	Male	Both	Female	Male	Both	Female	Male
Chahar Mahaal and Bakhtiari	Deaths	64.1 (32 to 104.6)	68.4 (36.8 to 109.6)	59.8 (25.1 to 108.7)	67.2 (40.4 to 97.8)	57.7 (34.5 to 84.3)	76.8 (42.9 to 115.9)	4.9 (-18.8 to 56.7)	-15.5 (-41.5 to 31.3)	28.4 (-11.6 to 119.2)
	DALYs	1722.5 (911.2 to 2702.7)	1859.7 (1068.1 to 2833.1)	1600.1 (716.3 to 2819)	1887.5 (1228 to 2609.1)	1667.1 (1150 to 2295.6)	2110.5 (1281.8 to 3025)	9.6 (-14.3 to 58.8)	-10.4 (-34.3 to 25.8)	31.9 (-7.3 to 125.5)
	YLLs	1456.3 (763.3 to 2321.3)	1527.7 (853.6 to 2373.7)	1392.5 (612.1 to 2494.8)	1367.8 (871.8 to 1925.6)	1091.3 (706.5 to 1565.3)	1648.4 (967.1 to 2420.8)	-6.1 (-26.8 to 39.4)	-28.6 (-50.7 to 6.9)	18.4 (-18.7 to 105.6)
	YLDs	266.3 (137.1 to 429.6)	332 (182 to 517.9)	207.5 (90.1 to 355.7)	519.8 (313.2 to 758.1)	575.8 (366.3 to 835.1)	462.1 (260.6 to 702.7)	95.2 (63.9 to 163.1)	73.5 (44.3 to 125.1)	122.7 (75.9 to 257.8)
East Azarbayejan	Deaths	101.4 (57.3 to 155.4)	110.9 (65.8 to 167.1)	91.9 (45.3 to 155.7)	117.6 (75.3 to 164.2)	124.4 (78.5 to 176.4)	110.6 (65.6 to 165.2)	16 (-13.1 to 65.1)	12.1 (-22.1 to 67.3)	20.4 (-17.4 to 96.3)
	DALYs	2590.9 (1541.3 to 3834.3)	2786.2 (1731.2 to 4093.2)	2413.8 (1247.7 to 3916.1)	2896.3 (2031.8 to 3906.1)	2858.7 (2011.4 to 3788)	2926.6 (1924.2 to 4111.7)	11.8 (-13.7 to 54.3)	2.6 (-23.7 to 44)	21.2 (-14.2 to 90.4)
	YLLs	2221.4 (1291.1 to 3370.2)	2340.4 (1417.8 to 3546.3)	2113.6 (1079.7 to 3537.4)	2200.8 (1493.3 to 2995)	2089.7 (1420.3 to 2841.2)	2305.4 (1450 to 3324.5)	-0.9 (-25.6 to 40.3)	-10.7 (-36.1 to 31.6)	9.1 (-26 to 76.9)
	YLDs	369.4 (208.6 to 565)	445.8 (266.6 to 666.1)	300.1 (152.2 to 487.6)	695.4 (451.4 to 993.9)	768.9 (508.9 to 1086.1)	621.3 (378.9 to 916.7)	88.2 (61.5 to 139.8)	72.5 (46.5 to 113)	107 (68.7 to 205.8)
Fars	Deaths	93.6 (52.8 to 142.8)	96.8 (55 to 145.5)	88.7 (43 to 146.2)	102.7 (69.4 to 142.9)	104.3 (69.2 to 142.2)	102.2 (63.5 to 149.6)	9.7 (-17.3 to 62.3)	7.7 (-21 to 67.1)	15.3 (-23.2 to 96.3)
	DALYs	2474.6 (1457.3 to 3616.3)	2523.6 (1510.6 to 3639.9)	2408.3 (1227 to 3945.7)	2744.5 (1932.5 to 3648.8)	2604.3 (1867.5 to 3387.4)	2887.9 (1934 to 4014.2)	10.9 (-16 to 58.4)	3.2 (-21.2 to 52)	19.9 (-19.4 to 99.6)
	YLLs	2121.3 (1225.1 to 3159.7)	2095.1 (1219.9 to 3100.5)	2126.6 (1055.3 to 3498.3)	2060.5 (1431.4 to 2791.4)	1848.3 (1252.1 to 2480.4)	2275.1 (1471.8 to 3245.6)	-2.9 (-28.1 to 43.7)	-11.8 (-36.5 to 40.2)	7 (-30.2 to 87.6)
	YLDs	353.3 (201.4 to 537.3)	428.5 (252.6 to 630.9)	281.7 (146.2 to 456.2)	684.1 (442.4 to 983.5)	756.1 (498.9 to 1069.6)	612.7 (388.2 to 890.6)	93.6 (63.7 to 145.4)	76.4 (50.6 to 119.8)	117.5 (71.9 to 215.2)

Province	Measure	Attributed age-standardized rate (per 100,000)						% Change (1990 to 2019)		
		1990			2019					
		Both	Female	Male	Both	Female	Male	Both	Female	Male
Gilan	Deaths	99.6 (57 to 149.4)	106 (60.7 to 157.8)	89.8 (45.5 to 148)	106.9 (71.6 to 147.3)	114.3 (76.2 to 158.2)	100.3 (61.5 to 145)	7.3 (-16.5 to 51.1)	7.9 (-21 to 59.7)	11.7 (-21 to 77.8)
	DALYs	2650 (1584.8 to 3843.2)	2801.2 (1705.4 to 4078.3)	2460.5 (1292.1 to 3935.9)	2888.9 (2034.5 to 3816.3)	2880.6 (2048 to 3766.3)	2899.2 (1925.2 to 4004.7)	9 (-14.4 to 51)	2.8 (-20.7 to 43.9)	17.8 (-15.1 to 86.8)
	YLLs	2277.1 (1317.3 to 3342.2)	2351.4 (1392.2 to 3474.5)	2167.2 (1118.3 to 3518.8)	2167.4 (1509.9 to 2900.5)	2070.5 (1435.1 to 2799.5)	2268.1 (1454.6 to 3228.4)	-4.8 (-26.9 to 35.9)	-11.9 (-36.1 to 29.9)	4.7 (-28.3 to 71.7)
	YLDs	372.9 (214.6 to 568.9)	449.8 (268.8 to 670.5)	293.3 (152.1 to 476.5)	721.4 (460.1 to 1021.8)	810.1 (529.4 to 1139.8)	631.1 (391.9 to 915.6)	93.5 (66.2 to 144.2)	80.1 (54 to 124.9)	115.2 (74.6 to 202.7)
Golestan	Deaths	110.3 (65.7 to 163.6)	115.9 (71.8 to 170.8)	103.8 (53 to 169.5)	126.5 (85.7 to 169.9)	124 (86.2 to 168.1)	129.8 (80.5 to 182.5)	14.7 (-9.3 to 52.2)	7 (-18.3 to 48.5)	25 (-11.3 to 89.4)
	DALYs	3061.7 (1853.1 to 4437.4)	3239 (2066.7 to 4670.4)	2884.6 (1531.3 to 4557.6)	3533.6 (2481.9 to 4636.5)	3397.2 (2485.6 to 4449.9)	3677.1 (2385.2 to 5036.1)	15.4 (-8.6 to 52.3)	4.9 (-18.4 to 41.7)	27.5 (-10 to 93)
	YLLs	2637.4 (1569.2 to 3877)	2718.5 (1702.3 to 3982.6)	2551.8 (1327.8 to 4071.8)	2782 (1935.4 to 3705.3)	2556.8 (1833.8 to 3398.4)	3018.3 (1918.3 to 4178.8)	5.5 (-18.7 to 43.4)	-5.9 (-29.4 to 32.2)	18.3 (-18.9 to 86.2)
	YLDs	424.3 (244.9 to 642.7)	520.6 (315.5 to 771.6)	332.8 (178.2 to 532.9)	751.6 (483.2 to 1079.6)	840.4 (552.2 to 1197.1)	658.8 (409.4 to 962.6)	77.1 (54.1 to 119.8)	61.4 (40.6 to 96.5)	98 (61.2 to 179.5)
Hamadan	Deaths	88.3 (49.1 to 140.2)	96.6 (54.3 to 146.5)	80.1 (38.7 to 139.5)	94.3 (62.1 to 128.9)	95.6 (62.7 to 132.3)	95 (58.3 to 138.3)	6.8 (-17.3 to 52)	-1 (-27.6 to 49.6)	18.6 (-18.1 to 90.4)
	DALYs	2415.8 (1371.9 to 3670.4)	2577.1 (1545.1 to 3771.9)	2269.1 (1145.4 to 3818.1)	2649.4 (1856.4 to 3505.1)	2479.3 (1772.1 to 3266.9)	2826.7 (1850 to 3964.8)	9.7 (-14.4 to 53.2)	-3.8 (-28.2 to 35.7)	24.6 (-12.7 to 100.3)
	YLLs	2092.4 (1172.1 to 3237.1)	2172.7 (1267.4 to 3255.4)	2020.7 (1002.2 to 3471.4)	2035.8 (1388.1 to 2748.8)	1789.5 (1242.8 to 2438.4)	2289.6 (1456.1 to 3266.9)	-2.7 (-25.3 to 37.2)	-17.6 (-40.3 to 20.8)	13.3 (-23.3 to 86.2)
	YLDs	323.4 (181.3 to 499.7)	404.4 (236.7 to 610.1)	248.4 (121.7 to 408.8)	613.7 (392.1 to 871.1)	689.8 (444.6 to 967.8)	537.1 (337.9 to 791.3)	89.7 (62.3 to 147.8)	70.5 (44.7 to 113.6)	116.2 (73.8 to 232.9)

Province	Measure	Attributed age-standardized rate (per 100,000)						% Change (1990 to 2019)		
		1990			2019					
		Both	Female	Male	Both	Female	Male	Both	Female	Male
Hormozgan	Deaths	101.2 (56.3 to 155.5)	107.7 (62.3 to 162.7)	94.1 (44.1 to 159.2)	96.8 (65.3 to 130.4)	99.2 (65.2 to 134.5)	96.4 (58.9 to 139.1)	-4.4 (-26.9 to 41.9)	-7.9 (-33.1 to 38.8)	2.5 (-29.7 to 80.5)
	DALYs	2704.5 (1550.7 to 4016.4)	2880.2 (1743.7 to 4192.9)	2536.8 (1258.3 to 4144)	2720.9 (1897.6 to 3632.4)	2582.1 (1843 to 3366.5)	2870 (1871.2 to 4046.6)	0.6 (-22.9 to 50.6)	-10.4 (-33.2 to 30.7)	13.1 (-21.5 to 95.1)
	YLLs	2366.5 (1325.7 to 3577.9)	2460.8 (1475.3 to 3607.4)	2270.4 (1104.7 to 3777.9)	2059 (1420.4 to 2761.3)	1853.9 (1275 to 2465)	2273.8 (1424.6 to 3252.5)	-13 (-34.5 to 35.8)	-24.7 (-45.4 to 15.6)	0.2 (-32 to 79.9)
	YLDs	338 (187.6 to 524.5)	419.4 (248.8 to 630.6)	266.5 (132.1 to 440)	662 (426.1 to 948.9)	728.2 (475.2 to 1033.9)	596.2 (369.5 to 873.8)	95.8 (67.2 to 155.4)	73.6 (48.3 to 119.1)	123.7 (78.4 to 254.8)
Ilam	Deaths	78.1 (40.5 to 125.3)	86.8 (46.9 to 134.7)	71.3 (31.5 to 126.5)	106.6 (71.9 to 145.9)	107 (72.9 to 145.2)	106.4 (66.3 to 153.8)	36.5 (4.2 to 108.2)	23.3 (-11.9 to 89.4)	49.2 (3.6 to 179.2)
	DALYs	2085.5 (1147.4 to 3228.1)	2310.1 (1324.9 to 3440.9)	1915.7 (886 to 3275.8)	2822.9 (1981.1 to 3740.9)	2759.9 (2004.1 to 3615.1)	2883.5 (1876.7 to 3965.7)	35.4 (4.7 to 109.6)	19.5 (-10.9 to 81.3)	50.5 (6.2 to 176.2)
	YLLs	1801.8 (960.9 to 2833)	1948 (1088 to 2953.1)	1694.2 (765.2 to 2907.3)	2164.9 (1498.3 to 2907)	2028.8 (1429.5 to 2676)	2296.6 (1473 to 3198.9)	20.2 (-8.5 to 86.9)	4.1 (-24.3 to 62.9)	35.6 (-5.5 to 155.3)
	YLDs	283.7 (150.3 to 452.7)	362.1 (205.3 to 557.3)	221.5 (103 to 376.8)	658.1 (421.3 to 942.3)	731.1 (470.3 to 1042.1)	586.9 (367.3 to 854.8)	132 (91 to 230.5)	101.9 (67.9 to 168.9)	165 (104.3 to 357.5)
Isfahan	Deaths	85.1 (47.3 to 128.6)	90.5 (50.9 to 138.6)	77.4 (37.9 to 125.2)	89.7 (59.2 to 123)	94.8 (62.8 to 131)	86.2 (52.6 to 126.2)	5.3 (-20.3 to 52.5)	4.7 (-26.4 to 64.5)	11.4 (-23.8 to 85.4)
	DALYs	2244.8 (1327.9 to 3264.4)	2323.6 (1400.9 to 3393.1)	2139 (1129.2 to 3295.6)	2456.4 (1732.3 to 3277.7)	2395.8 (1731.8 to 3127.6)	2524.8 (1663 to 3567.1)	9.4 (-15.5 to 53.3)	3.1 (-23.6 to 48.4)	18 (-17.6 to 91.7)
	YLLs	1858.5 (1026.8 to 2741.8)	1868.8 (1070.7 to 2812.1)	1818.5 (915.7 to 2901.9)	1758 (1206.7 to 2364.1)	1628.8 (1143.4 to 2201.4)	1892.7 (1195.9 to 2753.5)	-5.4 (-29.6 to 38.5)	-12.8 (-38.7 to 37.8)	4.1 (-30.3 to 74.7)
	YLDs	386.3 (225.3 to 590.1)	454.8 (274.3 to 679.1)	320.5 (173.3 to 510.8)	698.4 (451.3 to 995.5)	767 (506 to 1091.7)	632.1 (399.6 to 920.2)	80.8 (54.9 to 126.7)	68.6 (44.4 to 109.5)	97.2 (59 to 177.9)

Province	Measure	Attributed age-standardized rate (per 100,000)						% Change (1990 to 2019)		
		1990			2019					
		Both	Female	Male	Both	Female	Male	Both	Female	Male
Kerman	Deaths	108.9 (63.5 to 159.5)	109.7 (66.8 to 159.5)	106.6 (55.8 to 168.7)	91.5 (60.8 to 126.8)	95 (64.1 to 134.1)	88.2 (54.5 to 129.7)	-16 (-34.7 to 11.7)	-13.4 (-37.1 to 19.1)	-17.3 (-40 to 23.9)
	DALYs	2909.1 (1734.9 to 4124.9)	2889.3 (1826.4 to 4132)	2913.7 (1572.7 to 4468.6)	2482.3 (1738.3 to 3329.6)	2418.7 (1733.8 to 3217.8)	2542.2 (1657.9 to 3576.6)	-14.7 (-32.1 to 11.4)	-16.3 (-34.5 to 10)	-12.7 (-36.2 to 28.6)
	YLLs	2525.6 (1495 to 3652.9)	2432.5 (1515.2 to 3494.4)	2599.2 (1379 to 4012.2)	1834.1 (1267 to 2509.7)	1698.8 (1197.2 to 2320.6)	1964.4 (1242.2 to 2791.6)	-27.4 (-43.7 to -3.1)	-30.2 (-48.3 to -5.2)	-24.4 (-46.1 to 14.4)
	YLDs	383.5 (225.9 to 579.9)	456.8 (277.3 to 678.5)	314.5 (168.8 to 495.5)	648.1 (416.7 to 933.4)	719.9 (474.2 to 1017.7)	577.8 (358.7 to 846.2)	69 (47.3 to 107.1)	57.6 (38.4 to 91)	83.7 (52.2 to 152.1)
Kermanshah	Deaths	104.2 (57.5 to 161.3)	109.9 (62.6 to 166.8)	99 (47.6 to 165.3)	97.4 (64.5 to 132.5)	102.4 (69.3 to 138.5)	94.2 (57.6 to 137.4)	-6.5 (-28.3 to 39.9)	-6.9 (-32.9 to 40)	-4.8 (-33.6 to 63.6)
	DALYs	2838.7 (1601.6 to 4275.2)	2942 (1717.1 to 4326.9)	2758.6 (1380 to 4471.1)	2742.8 (1907.4 to 3637.8)	2689.8 (1923.8 to 3562)	2810.1 (1827 to 3945.8)	-3.4 (-25.2 to 40.3)	-8.6 (-32.1 to 32)	1.9 (-28.1 to 73.1)
	YLLs	2497.4 (1410.3 to 3765.1)	2514.1 (1421.8 to 3826.7)	2491.2 (1226.5 to 4119.5)	2078.6 (1414.9 to 2779.8)	1947.1 (1369.7 to 2577.1)	2225.3 (1389.4 to 3188.7)	-16.8 (-37.2 to 24.4)	-22.6 (-44.7 to 18)	-10.7 (-38.3 to 56.3)
	YLDs	341.3 (191.1 to 529.5)	427.9 (250.5 to 651.3)	267.4 (134.4 to 440.8)	664.1 (425.5 to 950.6)	742.7 (488.3 to 1060.1)	584.7 (363.2 to 858.3)	94.6 (64.4 to 149.9)	73.5 (47.7 to 115.8)	118.7 (76.2 to 226.6)
Khorasan-e-Razavi	Deaths	105.1 (58.4 to 160.5)	115.9 (66.8 to 176)	94.1 (44.5 to 156.8)	98.2 (65.1 to 134.7)	102.1 (66.9 to 140.2)	94.2 (57.9 to 136.2)	-6.6 (-27.3 to 29.7)	-11.9 (-36 to 28.2)	0.1 (-27.6 to 70.7)
	DALYs	2850 (1663.5 to 4236.9)	3097.4 (1901.5 to 4481.3)	2618.9 (1303.1 to 4210)	2806.8 (1954.4 to 3717.4)	2747.5 (1967.6 to 3605)	2863.1 (1851.6 to 3914.9)	-1.5 (-22.9 to 34.9)	-11.3 (-32.7 to 23.9)	9.3 (-21 to 78.9)
	YLLs	2419 (1381.8 to 3661.9)	2589.4 (1509.1 to 3827.9)	2259 (1095.7 to 3710.5)	2001.7 (1363.5 to 2662.5)	1902.8 (1303.4 to 2559.2)	2098.6 (1322 to 2920.7)	-17.3 (-36.8 to 16.5)	-26.5 (-46.7 to 6.9)	-7.1 (-35.6 to 59.3)
	YLDs	431.1 (244.8 to 659.3)	508 (302.1 to 757.5)	359.9 (184.7 to 587.1)	805.2 (520.2 to 1148.8)	844.7 (550.3 to 1187.3)	764.4 (476.8 to 1117)	86.8 (60.6 to 136.8)	66.3 (42.7 to 104)	112.4 (72.2 to 215.7)

Province	Measure	Attributed age-standardized rate (per 100,000)						% Change (1990 to 2019)		
		1990			2019					
		Both	Female	Male	Both	Female	Male	Both	Female	Male
Khuzestan	Deaths	103.5 (59.1 to 157)	110.7 (64.6 to 165)	94.6 (47.7 to 154.9)	115.1 (78.5 to 157.3)	119.7 (82 to 166.2)	110.8 (70 to 161.9)	11.2 (-11.8 to 52.3)	8.1 (-20.8 to 55.4)	17.1 (-18.8 to 87.6)
	DALYs	2814.5 (1685.8 to 4124.9)	2984.6 (1842.5 to 4328.2)	2631.4 (1351 to 4229.2)	3281.9 (2338.5 to 4318.1)	3237.1 (2365.6 to 4220.4)	3319.1 (2209.7 to 4645.1)	16.6 (-7.4 to 55.6)	8.5 (-17.1 to 50.4)	26.1 (-11.2 to 97.7)
	YLLs	2408.7 (1408.9 to 3587.3)	2498 (1491.3 to 3706.6)	2302.2 (1173.7 to 3759.3)	2449.9 (1718.7 to 3287.5)	2328.1 (1643.3 to 3112.3)	2564.4 (1637.9 to 3680.6)	1.7 (-21.4 to 39.4)	-6.8 (-30.9 to 36.1)	11.4 (-25 to 79.9)
	YLDs	405.8 (231.3 to 614.3)	486.6 (291.8 to 724.8)	329.2 (173.6 to 526.3)	832 (532.2 to 1180.2)	909 (584.6 to 1285.8)	754.7 (471.8 to 1090)	105 (74.3 to 156.3)	86.8 (60.7 to 130.7)	129.3 (85.6 to 224.5)
Kohgiluyeh and Boyer-Ahmad	Deaths	70.9 (36.9 to 113)	83 (44.4 to 130.9)	57.6 (24.8 to 102.3)	83.4 (54.6 to 117.8)	93.7 (61 to 133.9)	77 (44.9 to 113.6)	17.7 (-13.8 to 81.7)	13 (-22.4 to 83.1)	33.7 (-12.1 to 146.7)
	DALYs	1907.2 (1060.1 to 2931.8)	2193.5 (1267.4 to 3279.4)	1626.4 (749 to 2752.5)	2344.7 (1599.2 to 3174.1)	2412.1 (1716.9 to 3280)	2318.9 (1498.9 to 3308.8)	22.9 (-7.4 to 86.3)	10 (-20 to 63)	42.6 (-5.5 to 156.7)
	YLLs	1619.4 (877.6 to 2507.1)	1829.9 (1027.4 to 2819.4)	1406.4 (628.3 to 2458.4)	1745.3 (1154.8 to 2423.1)	1743.5 (1159.2 to 2457.2)	1782.1 (1096 to 2593.1)	7.8 (-20.8 to 65.3)	-4.7 (-34.3 to 50.7)	26.7 (-18.4 to 131.6)
	YLDs	287.7 (153.4 to 461.6)	363.7 (205.4 to 553.9)	220.1 (100.3 to 380.6)	599.3 (388.1 to 871.6)	668.6 (436.2 to 962.6)	536.9 (334.3 to 792.8)	108.3 (72.6 to 187.6)	83.9 (51.8 to 140.8)	144 (89.1 to 318.1)
Kurdistan	Deaths	83.5 (43.9 to 133)	91.9 (52.9 to 142.4)	76.5 (33.6 to 133.4)	86.1 (56.4 to 117.7)	90.7 (60.4 to 123.7)	83.3 (49.9 to 122.7)	3.2 (-19.9 to 53)	-1.3 (-28.6 to 46.7)	8.8 (-25.2 to 88.6)
	DALYs	2288.7 (1255 to 3545)	2523 (1519.6 to 3729.2)	2096.1 (968.3 to 3562.6)	2399.9 (1644.1 to 3158.3)	2369.8 (1674.4 to 3162.5)	2444.2 (1562.1 to 3439.3)	4.9 (-18.7 to 51.5)	-6.1 (-28.4 to 33.2)	16.6 (-18.4 to 104.4)
	YLLs	1990.7 (1082.8 to 3133.5)	2144 (1252.8 to 3240.6)	1867.8 (838.5 to 3220.4)	1786.1 (1200.7 to 2375.4)	1679.4 (1150.5 to 2232.9)	1905.9 (1186.1 to 2717.6)	-10.3 (-31.4 to 31.5)	-21.7 (-42.8 to 14.8)	2 (-30.6 to 78.8)
	YLDs	297.9 (160.4 to 469.8)	378.9 (215.6 to 581.3)	228.3 (104.4 to 388.5)	613.8 (387.6 to 880.2)	690.4 (441.6 to 976.2)	538.3 (326 to 804.6)	106 (73.5 to 180.5)	82.2 (53 to 139)	135.8 (86.8 to 285.4)

Province	Measure	Attributed age-standardized rate (per 100,000)						% Change (1990 to 2019)		
		1990			2019					
		Both	Female	Male	Both	Female	Male	Both	Female	Male
Lorestan	Deaths	92.5 (51.3 to 140.4)	98 (56.9 to 143.8)	87.1 (41.1 to 144.5)	102.8 (68.1 to 140.7)	88.1 (55.6 to 121.2)	118.3 (75 to 170.5)	11.1 (-13.9 to 56.9)	-10.1 (-36.5 to 33.4)	35.8 (-3.7 to 125.6)
	DALYs	2509.7 (1448.8 to 3724.1)	2653.3 (1609.6 to 3784.1)	2381.8 (1195.5 to 3853.2)	2772.2 (1915 to 3698.9)	2376 (1660.2 to 3167.3)	3190 (2080.8 to 4417.1)	10.5 (-14.8 to 54.9)	-10.5 (-33.7 to 24.3)	33.9 (-5.7 to 120)
	YLLs	2163.7 (1238.2 to 3241.6)	2224.3 (1308.3 to 3243.3)	2107.8 (1035.3 to 3481.7)	2133.1 (1449.9 to 2905.7)	1674.9 (1098.3 to 2291.8)	2614.8 (1671.7 to 3708.7)	-1.4 (-25.8 to 40.7)	-24.7 (-48 to 10.3)	24.1 (-14.9 to 106.5)
	YLDs	346.1 (194.9 to 537)	429 (248 to 642.8)	274 (139 to 448.9)	639.1 (410 to 912.5)	701 (460.6 to 989.9)	575.2 (355.8 to 849.7)	84.7 (57.2 to 135.5)	63.4 (39.9 to 105.2)	109.9 (69.5 to 213.6)
Markazi	Deaths	93.9 (52.5 to 141.2)	99.6 (57.3 to 147.7)	87.8 (41.8 to 144.7)	87 (57.9 to 120.1)	90.8 (60.7 to 125.3)	83.7 (51 to 120.8)	-7.3 (-28.9 to 33.4)	-8.8 (-34.2 to 36.2)	-4.7 (-34.7 to 57.1)
	DALYs	2526.4 (1511.5 to 3713)	2633.3 (1641.8 to 3772.7)	2424.7 (1230.1 to 3888.7)	2454.2 (1717.8 to 3243.1)	2383 (1767.2 to 3132.4)	2519.3 (1621.9 to 3463.2)	-2.9 (-24 to 35.2)	-9.5 (-31.1 to 24.6)	3.9 (-26.3 to 69.3)
	YLLs	2153.2 (1251.1 to 3203.6)	2185.4 (1301.6 to 3178.5)	2124.3 (1054.8 to 3474)	1772.2 (1216.7 to 2403.3)	1631.1 (1162.5 to 2213.3)	1908.1 (1203.2 to 2715.4)	-17.7 (-36.8 to 17.6)	-25.4 (-46.3 to 8)	-10.2 (-37.8 to 49.1)
	YLDs	373.3 (216.5 to 569)	448 (267.9 to 664.9)	300.4 (159.4 to 485)	682 (436.3 to 988.9)	751.9 (499.6 to 1059.2)	611.2 (379.1 to 900.8)	82.7 (56.6 to 132.8)	67.8 (44.7 to 111.8)	103.5 (63.2 to 195.4)
Mazandaran	Deaths	87.1 (50.3 to 130.7)	98 (57.9 to 147.5)	74.6 (38.4 to 124.1)	106.3 (72.8 to 144.6)	111.9 (77.7 to 149.2)	100.5 (63 to 146.3)	22.1 (-5 to 70.3)	14.1 (-16.9 to 65.9)	34.7 (-8.4 to 120.4)
	DALYs	2426.6 (1494.9 to 3457.4)	2732.5 (1735.1 to 3951.6)	2115.3 (1128.5 to 3352.8)	3020 (2141 to 3951.7)	3073.6 (2249 to 4039.9)	2958.4 (1992.4 to 4069.8)	24.5 (-1.6 to 66.7)	12.5 (-14 to 50.8)	39.9 (-2.1 to 119.3)
	YLLs	1958 (1187.2 to 2894.8)	2137.9 (1300 to 3181.6)	1771 (921 to 2882.7)	2182.8 (1546.5 to 2908)	2101.8 (1467.4 to 2803.2)	2257.7 (1464.9 to 3199.7)	11.5 (-13.9 to 54)	-1.7 (-28.6 to 39.7)	27.5 (-14.8 to 109.4)
	YLDs	468.6 (275 to 709.3)	594.6 (357.6 to 888)	344.3 (181.5 to 550.8)	837.2 (543.6 to 1197.1)	971.8 (637.3 to 1386.9)	700.7 (426.3 to 1006.7)	78.7 (53 to 123.2)	63.4 (40 to 101.3)	103.5 (61.7 to 182.4)

Province	Measure	Attributed age-standardized rate (per 100,000)						% Change (1990 to 2019)		
		1990			2019					
		Both	Female	Male	Both	Female	Male	Both	Female	Male
North Khorasan	Deaths	102 (56.5 to 156.7)	117.4 (67.3 to 175.7)	87 (41.3 to 148.7)	101.4 (67.3 to 138.6)	114 (76.1 to 157.2)	91.6 (55 to 131.1)	-0.7 (-22 to 39.1)	-3 (-29.1 to 39.3)	5.3 (-24.1 to 72.1)
	DALYs	2802.3 (1627.5 to 4111.2)	3181.3 (1931.2 to 4550.1)	2456.2 (1222.1 to 4016.6)	2922.2 (2030.4 to 3875.3)	3050 (2186.6 to 4021.7)	2817.1 (1837.7 to 3863.2)	4.3 (-17.3 to 41.8)	-4.1 (-26 to 32.3)	14.7 (-17.2 to 86.6)
	YLLs	2363 (1350.4 to 3521.8)	2652.9 (1557.8 to 3844)	2098.2 (1027 to 3528.5)	2124.8 (1460.4 to 2857.5)	2190 (1528.8 to 2943.1)	2081.8 (1310.5 to 2936.3)	-10.1 (-30.4 to 23.8)	-17.4 (-39.6 to 18.8)	-0.8 (-29.9 to 60.9)
	YLDs	439.4 (248.1 to 682.8)	528.4 (312 to 798.1)	358.1 (175.4 to 587)	797.4 (513 to 1144.2)	860 (561.3 to 1203.1)	735.3 (454.5 to 1087.5)	81.5 (54.8 to 139.7)	62.8 (38.7 to 106.1)	105.4 (64.4 to 234.4)
Qazvin	Deaths	80.3 (45.6 to 122.4)	87 (50.5 to 131.9)	73.1 (36.4 to 123.5)	87.9 (58.1 to 121)	78.6 (52.7 to 108.1)	96.8 (60.5 to 137.7)	9.5 (-15.8 to 55.6)	-9.6 (-37 to 42)	32.4 (-4.8 to 109.4)
	DALYs	2156.6 (1268.5 to 3212.2)	2352.2 (1424 to 3439.3)	1969.1 (1038.4 to 3233.3)	2415 (1680.7 to 3220.8)	2171.7 (1561.8 to 2881)	2652.2 (1758.2 to 3654.9)	12 (-12.3 to 52.5)	-7.7 (-30.9 to 32.5)	34.7 (-3.5 to 110.3)
	YLLs	1816.4 (1057.8 to 2776.5)	1930.6 (1147.9 to 2874)	1706.1 (885.6 to 2838.1)	1763.4 (1194.2 to 2401.6)	1444.9 (999.7 to 1964)	2078.5 (1341.8 to 2913.7)	-2.9 (-26.2 to 35.7)	-25.2 (-47.2 to 15.6)	21.8 (-14.8 to 95.6)
	YLDs	340.1 (192.8 to 523.9)	421.6 (248.6 to 630)	263 (134.6 to 431.4)	651.6 (423.8 to 915)	726.8 (482.1 to 1015.4)	573.7 (356.8 to 832.6)	91.6 (62.8 to 147.4)	72.4 (46.1 to 118.8)	118.2 (76 to 226)
Qom	Deaths	94.7 (54.7 to 145.6)	104.6 (60.4 to 159.9)	83.7 (42.2 to 135.7)	82.9 (55.3 to 112.8)	96.4 (65.3 to 129.6)	74.7 (45.6 to 106.8)	-12.4 (-33.4 to 28.1)	-7.9 (-35.1 to 43.2)	-10.8 (-37.9 to 49)
	DALYs	2508.8 (1515.9 to 3664.6)	2711.7 (1666.7 to 3934.3)	2300.4 (1237.4 to 3626.6)	2321.2 (1628.8 to 3051.5)	2445.8 (1772.1 to 3197.9)	2235.8 (1437.1 to 3098.8)	-7.5 (-28.6 to 33.2)	-9.8 (-32.1 to 32.5)	-2.8 (-32.2 to 62.3)
	YLLs	2107.3 (1230.4 to 3153.8)	2229.7 (1301.6 to 3320.9)	1973.9 (1020.9 to 3165.7)	1632.3 (1113.4 to 2154.3)	1672.5 (1181.4 to 2231.7)	1624.7 (1034.9 to 2290.1)	-22.5 (-42 to 15.7)	-25 (-46.6 to 16.8)	-17.7 (-44.3 to 43.5)
	YLDs	401.5 (231.8 to 609.2)	482 (293.2 to 718.9)	326.5 (172.6 to 517.4)	688.8 (445.4 to 986.5)	773.3 (511.7 to 1087.6)	611.1 (381.1 to 900)	71.6 (44 to 117.6)	60.4 (37.6 to 101.3)	87.1 (50.7 to 163.8)

Province	Measure	Attributed age-standardized rate (per 100,000)						% Change (1990 to 2019)		
		1990			2019					
		Both	Female	Male	Both	Female	Male	Both	Female	Male
Semnan	Deaths	87.8 (49.9 to 134.4)	89.9 (50.8 to 136)	84.5 (42.5 to 138.8)	100.9 (68 to 136.2)	90.9 (62.2 to 122.4)	109.9 (68.5 to 154.6)	14.9 (-11.7 to 66.7)	1.1 (-26.4 to 55.2)	30 (-7.3 to 112.7)
	DALYs	2400.4 (1409.4 to 3568.9)	2466.1 (1503.1 to 3608.3)	2326.1 (1209.1 to 3705.5)	2771.2 (1935.4 to 3643.7)	2520.4 (1852.6 to 3310.3)	3009.2 (2005.7 to 4095.8)	15.4 (-8.7 to 59.8)	2.2 (-21.4 to 43.9)	29.4 (-6.9 to 108.2)
	YLLs	1994.2 (1142.8 to 2995.2)	1974.9 (1138.9 to 2945.6)	2004.5 (1035.5 to 3257.7)	2008.2 (1378.1 to 2652.8)	1664.2 (1186.9 to 2228.5)	2342.4 (1517 to 3233.7)	0.7 (-22.9 to 45.2)	-15.7 (-38.6 to 26.6)	16.9 (-17.5 to 91.4)
	YLDs	406.2 (231.7 to 619.9)	491.1 (291.2 to 729)	321.6 (162.6 to 522)	762.9 (492.2 to 1082.1)	856.2 (560.6 to 1198.1)	666.9 (413.9 to 970)	87.8 (58.6 to 141.2)	74.3 (48.8 to 120.8)	107.3 (65.6 to 202.7)
Sistan and Baluchistan	Deaths	79.1 (39.8 to 128)	86.8 (46.4 to 135.1)	73.3 (31.5 to 130.7)	91.8 (59 to 126.3)	95.5 (61.6 to 134.5)	88.6 (51.6 to 132.4)	16.1 (-13.5 to 83.6)	10.1 (-24.4 to 70.9)	20.9 (-18.4 to 128.1)
	DALYs	2180.6 (1136.5 to 3408)	2410.9 (1347.4 to 3660.1)	2004.8 (881.6 to 3487.5)	2723.3 (1845.6 to 3632.8)	2751.8 (1891.5 to 3713.2)	2703.8 (1666.1 to 3848.9)	24.9 (-6.9 to 92.4)	14.1 (-18.3 to 73.5)	34.9 (-7.9 to 154.9)
	YLLs	1893.4 (960.7 to 3023.4)	2053.8 (1118.7 to 3132.7)	1773.6 (755 to 3154.4)	2175.9 (1436.2 to 2946.8)	2148.9 (1419.4 to 2948.4)	2211.2 (1307.6 to 3202.7)	14.9 (-15.5 to 85.7)	4.6 (-28.5 to 67.6)	24.7 (-17.7 to 148)
	YLDs	287.3 (150.5 to 457.8)	357.1 (201.3 to 551.6)	231.2 (102.6 to 396.2)	547.4 (345.6 to 793.5)	602.8 (389.7 to 860.9)	492.7 (289.8 to 739.5)	90.6 (61.4 to 156.2)	68.8 (43.1 to 119.3)	113.1 (70 to 242)
South Khorasan	Deaths	71 (38.5 to 111)	79.6 (45 to 122.6)	62.9 (28.6 to 109.1)	73.7 (48.2 to 102.4)	75.1 (50.1 to 105.6)	72.5 (44.1 to 106.8)	3.8 (-20 to 50.9)	-5.7 (-31.4 to 36.9)	15.3 (-20 to 106.9)
	DALYs	2043.7 (1163.1 to 3051.8)	2310.9 (1399.8 to 3324.2)	1801.9 (861.1 to 3004.5)	2326.2 (1621.2 to 3077.8)	2375.2 (1712.9 to 3108.4)	2276.9 (1479.1 to 3149.1)	13.8 (-10.2 to 60.3)	2.8 (-19 to 41.5)	26.4 (-10.3 to 115.1)
	YLLs	1593.8 (880.6 to 2458.6)	1744.2 (1012.4 to 2601.7)	1459.1 (673.8 to 2515.4)	1463.4 (994.2 to 1977.8)	1385.9 (976 to 1882.2)	1546.8 (983.9 to 2228.9)	-8.2 (-29 to 31.6)	-20.5 (-41.1 to 12.9)	6 (-27.6 to 84.8)
	YLDs	449.9 (253.5 to 694.9)	566.7 (332.7 to 841.5)	342.9 (162.7 to 576.3)	862.8 (556.8 to 1241.7)	989.2 (655.5 to 1406.1)	730.1 (447.8 to 1069.4)	91.8 (62.2 to 153.1)	74.6 (48.5 to 123.3)	112.9 (67.9 to 249.6)

Province	Measure	Attributed age-standardized rate (per 100,000)						% Change (1990 to 2019)		
		1990			2019					
		Both	Female	Male	Both	Female	Male	Both	Female	Male
Tehran	Deaths	66.5 (40.5 to 99.4)	76.1 (46.4 to 115)	55.2 (30 to 91.6)	63.5 (42.9 to 85.1)	74.1 (50.1 to 100.2)	54.6 (34.2 to 79.2)	-4.5 (-28.3 to 31.2)	-2.6 (-30.1 to 44.5)	-1.2 (-34 to 50)
	DALYs	1900.6 (1222.5 to 2683.6)	2087.6 (1365.3 to 2979.1)	1692.8 (978.4 to 2652.5)	1959 (1395.3 to 2570.4)	2066.7 (1488.4 to 2720.9)	1863.4 (1251.8 to 2563.2)	3.1 (-19.2 to 34)	-1 (-23.6 to 31.4)	10.1 (-22.4 to 60.9)
	YLLs	1422 (890.5 to 2076.8)	1532.7 (976.5 to 2272)	1287.4 (718.9 to 2073.6)	1216.9 (838.2 to 1615)	1264 (875.8 to 1694.8)	1180.3 (744.2 to 1681.8)	-14.4 (-36.6 to 18)	-17.5 (-41.6 to 19.7)	-8.3 (-40.5 to 42.8)
	YLDs	478.5 (284.4 to 711.7)	554.9 (343.1 to 806.2)	405.4 (222.9 to 633.2)	742.1 (473.6 to 1061.9)	802.7 (517.1 to 1135.2)	683 (424.3 to 992.4)	55.1 (32.4 to 88.6)	44.7 (26.7 to 71.8)	68.5 (32.5 to 125.4)
West Azarbayejan	Deaths	95.4 (52.5 to 149.9)	104.7 (59.9 to 161)	86 (41.3 to 143.7)	101.8 (64.9 to 141.2)	108.8 (71 to 154.3)	93.9 (54.6 to 139.3)	6.6 (-17.5 to 51.3)	3.9 (-26.3 to 56.5)	9.2 (-22.3 to 74.4)
	DALYs	2476.4 (1435 to 3747.7)	2698.1 (1634.7 to 3944.3)	2273.7 (1158.7 to 3742.8)	2590.9 (1772.2 to 3492.8)	2622.9 (1863.1 to 3477.6)	2541 (1577.5 to 3618)	4.6 (-17.7 to 43.9)	-2.8 (-26.3 to 36.7)	11.8 (-18.7 to 75.7)
	YLLs	2144.4 (1217.9 to 3287.2)	2283.3 (1350 to 3380.7)	2017.5 (1008 to 3342.3)	1945.1 (1297.8 to 2665.3)	1887.7 (1300.5 to 2565.4)	1988.3 (1214.8 to 2885.5)	-9.3 (-30 to 25.5)	-17.3 (-39.2 to 19.7)	-1.4 (-30.4 to 57.7)
	YLDs	332 (185.3 to 519.3)	414.8 (242.3 to 630.9)	256.2 (126.6 to 425.3)	645.8 (410.2 to 928.5)	735.2 (471.5 to 1052.7)	552.7 (335.4 to 819.1)	94.5 (67.4 to 148.6)	77.3 (52.7 to 126.4)	115.7 (75.8 to 222.1)
Yazd	Deaths	92.4 (53.3 to 137.2)	95.4 (55.8 to 142.3)	86.5 (45.1 to 139.1)	100.7 (68.6 to 138.4)	99.6 (67 to 135.5)	99.8 (62.7 to 145)	8.9 (-16.2 to 56.2)	4.4 (-24.4 to 55.4)	15.4 (-19.8 to 85)
	DALYs	2438.2 (1478.8 to 3496.8)	2505.1 (1568.9 to 3593.9)	2340.7 (1246.1 to 3678.3)	2721.3 (1950.2 to 3580.6)	2576.8 (1866.2 to 3378.1)	2832.9 (1909 to 3911.1)	11.6 (-13.5 to 53.3)	2.9 (-20.6 to 44.3)	21 (-14.5 to 89.2)
	YLLs	2029.7 (1193.9 to 3003.6)	2029.8 (1207.2 to 3001.9)	1999.3 (1050.3 to 3245.6)	1918.1 (1355.5 to 2588)	1721.2 (1199.3 to 2320.7)	2081.8 (1359.1 to 2945.3)	-5.5 (-28.6 to 33.8)	-15.2 (-38.5 to 25.8)	4.1 (-29.2 to 69.7)
	YLDs	408.5 (237.9 to 617.5)	475.3 (284.8 to 700.9)	341.4 (185.1 to 540.3)	803.2 (524.6 to 1145.7)	855.5 (570.1 to 1204.5)	751.1 (473.2 to 1095.3)	96.6 (67.3 to 147.5)	80 (54.8 to 125.2)	120 (74.6 to 214.1)

Province	Measure	Attributed age-standardized rate (per 100,000)						% Change (1990 to 2019)		
		1990			2019					
		Both	Female	Male	Both	Female	Male	Both	Female	Male
Zanjan	Deaths	70.1 (38 to 110.5)	71.8 (39.2 to 109.8)	67.9 (32 to 115.2)	78.7 (51.9 to 108.8)	72.4 (47.2 to 98.3)	85.2 (52.3 to 124)	12.3 (-14.2 to 58.5)	0.8 (-27.1 to 52.4)	25.4 (-10.1 to 104.6)
	DALYs	1921.8 (1090.5 to 2914.9)	1995.6 (1183.9 to 2904.3)	1849.4 (918.8 to 3053.9)	2145.2 (1465.2 to 2828.9)	1970.7 (1399.5 to 2590.6)	2323.6 (1504.4 to 3189.1)	11.6 (-12.6 to 54.7)	-1.2 (-23.7 to 38.4)	25.6 (-9.8 to 101.3)
	YLLs	1606 (899.6 to 2477.2)	1608.1 (918.2 to 2416.4)	1601.3 (777 to 2723.1)	1551.7 (1049.4 to 2109.4)	1313.5 (909.7 to 1762.3)	1797.1 (1146.5 to 2559.5)	-3.4 (-25.8 to 34.9)	-18.3 (-39.5 to 19)	12.2 (-21.7 to 81.3)
	YLDs	315.7 (174.6 to 490.3)	387.5 (226.8 to 589.6)	248 (120.6 to 413.1)	593.6 (378 to 857.3)	657.3 (423.3 to 935.1)	526.5 (326.2 to 777.9)	88 (58.1 to 144.2)	69.6 (42.7 to 116.1)	112.3 (69.2 to 230.4)

*Data in parentheses are 95% Uncertainty Intervals (95% UIs)

Supplementary Table 8

Province	Measure	Attributed age-standardized rate (per 100,000)						% Change (1990 to 2019)		
		1990			2019					
		Both	Female	Male	Both	Female	Male	Both	Female	Male
Alborz	Deaths	135.4 (93 to 182.8)	129.8 (86.5 to 181.9)	138.7 (92.4 to 193.2)	90.5 (64.1 to 121.2)	96.3 (63.8 to 134.9)	91.4 (66.8 to 123.4)	-33.2 (-46 to -13.5)	-25.8 (-43.7 to 3.1)	-34.2 (-50.9 to -8.3)
	DALYs	2800.8 (2024.7 to 3656.3)	2435.5 (1694.9 to 3253.1)	3088.2 (2108.1 to 4249.7)	1709.1 (1363.3 to 2159.9)	1453.1 (1065.7 to 1924.1)	2005 (1550.9 to 2574.8)	-39 (-52.2 to -17.5)	-40.3 (-54.9 to -13.5)	-35.1 (-52.7 to -5.2)
	YLLs	2699.9 (1931.4 to 3511.7)	2325.3 (1599.4 to 3117.6)	2995.5 (2021.6 to 4144.3)	1618.4 (1281.9 to 2040.1)	1360.4 (986.3 to 1804.9)	1916.1 (1463.9 to 2480.3)	-40.1 (-53.4 to -17.5)	-41.5 (-56.2 to -13.8)	-36 (-53.9 to -5.2)
	YLDs	100.9 (66.9 to 145.8)	110.2 (71.9 to 161.1)	92.6 (60.8 to 132.2)	90.7 (60 to 130.4)	92.7 (60.1 to 137)	88.9 (58.9 to 126.6)	-10.1 (-14.9 to -4.6)	-15.8 (-22.7 to -8.3)	-4.1 (-11.4 to 3.9)
Ardebil	Deaths	144.9 (104.9 to 188.8)	124.7 (86.3 to 170)	162.9 (114.2 to 219.2)	100.6 (73.5 to 132.2)	82.7 (58.4 to 111.6)	119.1 (89.1 to 156.9)	-30.5 (-42.7 to -15.3)	-33.7 (-47 to -16.4)	-26.9 (-43.9 to -4.9)
	DALYs	3157 (2407 to 3970.8)	2550.7 (1886.1 to 3325.2)	3700.4 (2672 to 4922.9)	1971 (1580.3 to 2404.8)	1467.7 (1137.8 to 1840.7)	2481.5 (1983.4 to 3093)	-37.6 (-49.9 to -22.7)	-42.5 (-55.1 to -26)	-32.9 (-49.9 to -10.8)
	YLLs	3065 (2323 to 3883.5)	2452.2 (1801.7 to 3206.9)	3614.7 (2609.3 to 4824.9)	1877.8 (1494.2 to 2295.1)	1372.9 (1063.8 to 1729.2)	2389.7 (1910.6 to 2987)	-38.7 (-51.4 to -23.5)	-44 (-57 to -27)	-33.9 (-51 to -11.3)
	YLDs	92 (60.9 to 132)	98.5 (64.9 to 144.3)	85.7 (56.5 to 121.7)	93.2 (62.1 to 134.2)	94.7 (62.6 to 138.2)	91.8 (60.9 to 128.5)	1.3 (-4.3 to 7.2)	-3.9 (-11 to 3.7)	7.1 (-1.5 to 16.2)
Bushehr	Deaths	164.9 (118.8 to 216.3)	141.4 (99.3 to 195.6)	187.3 (131.9 to 251.4)	102.2 (73.8 to 136.4)	88 (61.2 to 121.1)	116 (83.5 to 153.6)	-38 (-48.9 to -24.6)	-37.7 (-49.4 to -22.1)	-38.1 (-52.4 to -19.3)
	DALYs	3580.9 (2675.2 to 4505.7)	2888.9 (2161.6 to 3795.9)	4229.7 (3014.9 to 5562.7)	1961.2 (1538.6 to 2466.5)	1554.4 (1180.5 to 1993.6)	2346.3 (1817 to 2974.6)	-45.2 (-55.7 to -31.7)	-46.2 (-57.3 to -30.4)	-44.5 (-58.3 to -25.2)
	YLLs	3470.4 (2573.8 to 4385.3)	2772.6 (2045.5 to 3662.8)	4124.6 (2934.8 to 5451.2)	1862.4 (1448.6 to 2335.8)	1452.6 (1103.3 to 1861.5)	2250.1 (1736 to 2850.4)	-46.3 (-56.9 to -32.4)	-47.6 (-58.7 to -31.5)	-45.4 (-59.3 to -25.7)
	YLDs	110.6 (73.2 to 160.1)	116.3 (76.5 to 171.6)	105.2 (69.3 to 149.6)	98.9 (65.2 to 142.1)	101.8 (66.2 to 149.5)	96.2 (63.2 to 136.4)	-10.6 (-15.7 to -5.4)	-12.4 (-19.5 to -5)	-8.5 (-15.3 to -1.7)

Province	Measure	Attributed age-standardized rate (per 100,000)						% Change (1990 to 2019)		
		1990			2019					
		Both	Female	Male	Both	Female	Male	Both	Female	Male
Chahar Mahaal and Bakhtiari	Deaths	155.1 (112.3 to 204.2)	131.4 (91.2 to 181.4)	177.4 (125.7 to 243.9)	94 (66.5 to 125.7)	72.1 (48.1 to 102.5)	116.8 (81.3 to 154.7)	-39.4 (-50.1 to -25.7)	-45.2 (-59.1 to -25.1)	-34.1 (-49.3 to -16.3)
	DALYs	3220.3 (2470.6 to 4100.7)	2585.1 (1919.6 to 3402)	3788.8 (2777.9 to 5100.5)	1769.3 (1361.7 to 2212.7)	1243.7 (939 to 1657.5)	2302.8 (1731 to 2922.3)	-45.1 (-55.8 to -31.5)	-51.9 (-64.6 to -34.2)	-39.2 (-54.1 to -19.8)
	YLLs	3131.3 (2407.4 to 3984.2)	2491.4 (1840.8 to 3273.6)	3704.2 (2698.5 to 5005.8)	1685.5 (1292.8 to 2115)	1160.7 (867.6 to 1553.6)	2218.2 (1656.9 to 2825.8)	-46.2 (-57.2 to -32.3)	-53.4 (-66.3 to -35.1)	-40.1 (-55.1 to -20.3)
	YLDs	88.9 (59.6 to 127.6)	93.6 (62.9 to 137.3)	84.5 (56.4 to 119.1)	83.8 (55.1 to 120.1)	83.1 (53.9 to 122.2)	84.7 (55.5 to 119.5)	-5.8 (-11.2 to -0.3)	-11.3 (-18.8 to -3.6)	0.2 (-7.8 to 8.3)
East Azarbayejan	Deaths	154.7 (109.4 to 208.6)	137.6 (91.5 to 195.1)	169.7 (117.2 to 233.5)	88.2 (60.7 to 121.8)	82.4 (54 to 118.6)	94 (64.5 to 127.7)	-43 (-54.2 to -29.4)	-40.2 (-54.5 to -21.9)	-44.6 (-58.5 to -26.3)
	DALYs	3160.8 (2354.3 to 4066.6)	2583.1 (1868 to 3543.6)	3683.9 (2618.3 to 5006.4)	1631.2 (1257.1 to 2113)	1326.5 (974.1 to 1817.8)	1931.4 (1447.3 to 2526.7)	-48.4 (-59.2 to -34.9)	-48.6 (-61.9 to -30.8)	-47.6 (-61.6 to -28.1)
	YLLs	3067.7 (2279.4 to 3977.8)	2482.1 (1777.8 to 3419.2)	3598.3 (2546.7 to 4909.1)	1544.9 (1181.9 to 2000.1)	1238.8 (894.1 to 1710.3)	1846.6 (1376.1 to 2421.6)	-49.6 (-60.6 to -35.8)	-50.1 (-63.5 to -31.8)	-48.7 (-62.9 to -28.8)
	YLDs	93.1 (61.1 to 136.8)	101 (64.8 to 150.8)	85.6 (56.1 to 121.9)	86.2 (56.5 to 124)	87.7 (56.1 to 131.6)	84.8 (55.2 to 121.8)	-7.4 (-13.7 to -1.1)	-13.2 (-20.9 to -4.9)	-0.9 (-9.5 to 8)
Fars	Deaths	171.4 (122.9 to 223.7)	149.4 (99.8 to 205.8)	190.3 (137.2 to 255.9)	87.6 (60.2 to 119.6)	81.4 (53.5 to 115.8)	94.4 (65 to 129.9)	-48.9 (-59.3 to -35.9)	-45.5 (-58.6 to -28.8)	-50.4 (-62.9 to -30.4)
	DALYs	3586.6 (2711.3 to 4586.2)	2834.6 (1976.8 to 3725.2)	4263.3 (3067.8 to 5698.3)	1676.5 (1269.2 to 2165.9)	1340.4 (968.7 to 1793.7)	2012.6 (1511.6 to 2681.7)	-53.3 (-63.3 to -39)	-52.7 (-64.6 to -35.8)	-52.8 (-65.4 to -31.6)
	YLLs	3497.4 (2644.4 to 4472.5)	2738.2 (1897.5 to 3607.4)	4181 (2992.3 to 5605)	1599.5 (1213.1 to 2080.6)	1262 (906 to 1691)	1936.7 (1453.9 to 2595.4)	-54.3 (-64.4 to -40)	-53.9 (-66.1 to -36.4)	-53.7 (-66.3 to -32.2)
	YLDs	89.2 (59 to 130.2)	96.4 (63.4 to 143.7)	82.3 (54.2 to 118.6)	77.1 (50 to 111.5)	78.4 (50.3 to 117.3)	75.9 (49.9 to 109.2)	-13.6 (-19 to -8)	-18.6 (-25.5 to -10.9)	-7.8 (-14.6 to 0.2)

Province	Measure	Attributed age-standardized rate (per 100,000)						% Change (1990 to 2019)		
		1990			2019					
		Both	Female	Male	Both	Female	Male	Both	Female	Male
Gilan	Deaths	186.4 (136.3 to 245.8)	168.6 (118.3 to 229.8)	202.2 (144.6 to 274.9)	102.9 (75.4 to 136.2)	101.9 (70.1 to 139.9)	105 (76.3 to 139.9)	-44.8 (-54.2 to -32.5)	-39.6 (-52.7 to -21.9)	-48 (-61 to -30.8)
	DALYs	3914.7 (3007.6 to 4998.9)	3302.4 (2418.6 to 4354.5)	4493.6 (3316.4 to 5999.3)	1991.3 (1572.6 to 2459.9)	1710.5 (1287.8 to 2221.3)	2281.1 (1787.1 to 2891)	-49.1 (-58.7 to -35.8)	-48.2 (-60.4 to -31)	-49.2 (-63 to -30.1)
	YLLs	3808.7 (2923.1 to 4846.1)	3187.3 (2317.5 to 4193.5)	4396.9 (3246.8 to 5866.5)	1893.2 (1494.5 to 2338.2)	1610.4 (1204.9 to 2104.3)	2185.1 (1696.9 to 2764)	-50.3 (-59.8 to -36.7)	-49.5 (-61.7 to -31.9)	-50.3 (-64 to -30.9)
	YLDs	106 (70.4 to 151.9)	115.1 (75.8 to 167.3)	96.7 (63.7 to 136.3)	98.1 (65.2 to 140.6)	100.1 (66 to 147.3)	96 (64 to 135.4)	-7.5 (-13.2 to -1.6)	-13 (-19.8 to -5.6)	-0.7 (-8.7 to 7.2)
Golestan	Deaths	183.4 (136 to 238.4)	159.3 (112.5 to 215.6)	207.1 (151.5 to 272.9)	115.9 (85.8 to 149.5)	102.6 (73.9 to 136.4)	130.2 (96.3 to 169)	-36.8 (-46.3 to -23.5)	-35.6 (-48.6 to -18.2)	-37.1 (-50.8 to -17.7)
	DALYs	4146.7 (3221.3 to 5196.9)	3439.2 (2530.1 to 4514.9)	4808.7 (3568.6 to 6321.5)	2488.8 (2013.7 to 3036.8)	2055.8 (1600.9 to 2591.5)	2939 (2308.9 to 3668.3)	-40 (-50.5 to -25.5)	-40.2 (-54.1 to -22)	-38.9 (-53.4 to -17.6)
	YLLs	4036.7 (3121.8 to 5048.3)	3317.8 (2421.6 to 4361.6)	4709.8 (3488.9 to 6204.9)	2388 (1928.3 to 2910.5)	1950.3 (1504.1 to 2453.8)	2843.1 (2232.3 to 3550.1)	-40.8 (-51.4 to -26.1)	-41.2 (-55.2 to -22.3)	-39.6 (-54.3 to -17.9)
	YLDs	110 (73.1 to 159.5)	121.4 (79.5 to 178.9)	98.8 (64.9 to 142.4)	100.7 (66.6 to 145.1)	105.6 (68.4 to 155.4)	95.9 (63.3 to 135.9)	-8.4 (-14.1 to -3.3)	-13 (-20.7 to -5.5)	-3 (-10.3 to 5.2)
Hamadan	Deaths	158.1 (113.1 to 209.1)	146.3 (97.7 to 199.2)	167.2 (117.5 to 229.7)	95.5 (68.7 to 125.9)	91.4 (63.1 to 126.3)	102 (73 to 133.8)	-39.6 (-50.4 to -26.9)	-37.5 (-50.8 to -21.7)	-39 (-54.3 to -18.8)
	DALYs	3449.8 (2637.2 to 4416.8)	2869.3 (2037.7 to 3762.2)	3978.6 (2881.2 to 5314.1)	1966.9 (1530.8 to 2425.1)	1578.7 (1184.8 to 2045.1)	2367.7 (1775.9 to 2996.1)	-43 (-53.9 to -29)	-45 (-57.9 to -29.2)	-40.5 (-56.3 to -18.7)
	YLLs	3363.3 (2561.8 to 4296)	2773.3 (1962.6 to 3651.3)	3901.3 (2809.4 to 5231.4)	1884.5 (1459.2 to 2315.4)	1491.9 (1117 to 1955.5)	2289.3 (1717.3 to 2910.5)	-44 (-54.9 to -29.8)	-46.2 (-59.3 to -29.9)	-41.3 (-57.3 to -19.3)
	YLDs	86.4 (57.1 to 124.7)	96 (62.8 to 139.5)	77.3 (50.4 to 110.7)	82.5 (54.1 to 119.2)	86.7 (56.8 to 128.3)	78.3 (51.5 to 112.7)	-4.6 (-10 to 1.1)	-9.6 (-16.8 to -1.4)	1.4 (-6.4 to 9.4)

Province	Measure	Attributed age-standardized rate (per 100,000)						% Change (1990 to 2019)		
		1990			2019					
		Both	Female	Male	Both	Female	Male	Both	Female	Male
Hormozgan	Deaths	168.8 (119.9 to 223.9)	140.6 (95.5 to 190.9)	194.7 (129.2 to 268)	92.2 (67.2 to 119.3)	85.9 (60.2 to 116.2)	100.6 (74.3 to 131.9)	-45.4 (-55.2 to -28.7)	-38.9 (-51 to -22)	-48.3 (-60.9 to -27.3)
	DALYs	3778.3 (2622 to 4850.2)	2985.8 (2117 to 3866.5)	4461.3 (2888.8 to 5973.4)	1939.6 (1539.4 to 2368.9)	1540.1 (1172.6 to 1947.9)	2351 (1799.1 to 2978.4)	-48.7 (-58.7 to -26.6)	-48.4 (-60.2 to -27.2)	-47.3 (-61.3 to -20.8)
	YLLs	3685.1 (2532.6 to 4738.1)	2886.4 (2011.6 to 3775)	4373.4 (2814 to 5866.2)	1852.6 (1464.5 to 2270.5)	1451.9 (1093.5 to 1847.7)	2265 (1723.6 to 2881.8)	-49.7 (-59.9 to -27.7)	-49.7 (-61.5 to -27.9)	-48.2 (-62.3 to -21.2)
	YLDs	93.3 (61.6 to 133.1)	99.4 (64.3 to 145)	87.8 (58 to 125.5)	87 (57.7 to 125)	88.2 (57.4 to 129.5)	86 (57.1 to 122.8)	-6.7 (-12.4 to -1.3)	-11.3 (-18.7 to -3.4)	-2.1 (-9.3 to 6.2)
Ilam	Deaths	171.3 (121.5 to 224.8)	146.4 (100.9 to 195.5)	190.7 (135.5 to 257.2)	103 (74.5 to 137.2)	92.3 (64.8 to 124.6)	112.6 (80.6 to 150.6)	-39.9 (-49.6 to -27.7)	-36.9 (-48.7 to -17.7)	-41 (-53.6 to -25)
	DALYs	3625.2 (2719.2 to 4621.6)	2898.8 (2100.1 to 3746.9)	4195.7 (3093.1 to 5518.4)	1951.9 (1558.2 to 2417.2)	1626.8 (1267 to 2031.4)	2265.7 (1758.5 to 2870.9)	-46.2 (-55.7 to -33.6)	-43.9 (-55.6 to -25.8)	-46 (-58.6 to -28.2)
	YLLs	3543.3 (2653.8 to 4527.7)	2816.7 (2027.7 to 3653)	4114.4 (3018.4 to 5419)	1871.4 (1491.5 to 2316.7)	1547.7 (1200.3 to 1941.6)	2183.9 (1688.7 to 2789)	-47.2 (-56.6 to -34.4)	-45.1 (-56.6 to -26.7)	-46.9 (-59.5 to -28.8)
	YLDs	81.9 (54 to 115.1)	82.2 (54.8 to 116.9)	81.3 (53.7 to 114.5)	80.5 (53.4 to 114.4)	79.1 (52.3 to 114.9)	81.9 (54.2 to 115)	-1.7 (-7.2 to 3.9)	-3.7 (-12 to 4.5)	0.7 (-6 to 7.7)
Isfahan	Deaths	151.2 (105.1 to 201.9)	139 (93.8 to 195.1)	158.9 (106.9 to 217)	84.3 (59.8 to 113.1)	82.6 (54.2 to 116.2)	87.3 (63.1 to 115.8)	-44.2 (-54.7 to -29.1)	-40.5 (-54.2 to -21.3)	-45.1 (-59.5 to -25.9)
	DALYs	3067.2 (2271.4 to 3915.3)	2514.3 (1763.5 to 3452.8)	3541.2 (2512 to 4677.5)	1561.7 (1221 to 1961.8)	1279 (945.9 to 1702.6)	1845.4 (1428.5 to 2362.1)	-49.1 (-59.7 to -33.5)	-49.1 (-61.8 to -29.1)	-47.9 (-63.1 to -24)
	YLLs	2972.9 (2192.7 to 3809)	2415.5 (1684 to 3344.6)	3451.3 (2441.5 to 4576.6)	1481 (1157.4 to 1860.4)	1199.6 (870.9 to 1608.5)	1763.4 (1357.3 to 2271.1)	-50.2 (-60.8 to -34.4)	-50.3 (-63.3 to -29.6)	-48.9 (-64.2 to -24.7)
	YLDs	94.3 (62.1 to 134.4)	98.8 (64.5 to 144.4)	89.8 (59.2 to 127.2)	80.7 (53.4 to 114.1)	79.4 (51.3 to 116.6)	82 (54.3 to 115.3)	-14.4 (-19.9 to -8.7)	-19.6 (-26.8 to -12.1)	-8.7 (-15.9 to -1.7)

Province	Measure	Attributed age-standardized rate (per 100,000)						% Change (1990 to 2019)		
		1990			2019					
		Both	Female	Male	Both	Female	Male	Both	Female	Male
Kerman	Deaths	180.6 (134 to 235.1)	151.4 (108.6 to 203.2)	207.8 (151.9 to 279.2)	88 (61.3 to 119.8)	83.5 (55.8 to 116.7)	92.5 (64.8 to 123.8)	-51.3 (-60.5 to -41)	-44.8 (-57.1 to -30.9)	-55.5 (-66.2 to -41.9)
	DALYs	3925.6 (3034.8 to 4964.3)	3007.4 (2231.8 to 3897.5)	4768.9 (3573.6 to 6256.7)	1663.4 (1303.3 to 2097.6)	1364.7 (1024.4 to 1790.5)	1954 (1487.2 to 2503.9)	-57.6 (-66.1 to -47.4)	-54.6 (-64.6 to -41.1)	-59 (-69.4 to -45.2)
	YLLs	3833.9 (2964.6 to 4855.3)	2910.6 (2154.2 to 3796.8)	4682 (3492.2 to 6176.9)	1585.9 (1227.1 to 2001.3)	1286.6 (958.7 to 1693.1)	1877 (1418 to 2415.1)	-58.6 (-67.1 to -48.1)	-55.8 (-66 to -42)	-59.9 (-70.5 to -46)
	YLDs	91.7 (60.8 to 130)	96.8 (63.1 to 139.5)	86.9 (57.7 to 122.4)	77.5 (51.2 to 110.3)	78.1 (51.3 to 114)	77 (51.2 to 108.2)	-15.5 (-20.6 to -9.9)	-19.2 (-25.9 to -12.1)	-11.4 (-18.3 to -4.1)
Kermanshah	Deaths	182.1 (135.4 to 238.8)	149.5 (103.9 to 202.4)	209.5 (154 to 280.3)	98 (71 to 129.5)	91.5 (62.9 to 124.8)	106 (77 to 140.2)	-46.2 (-56 to -33.2)	-38.8 (-52.8 to -19.4)	-49.4 (-61.3 to -31.9)
	DALYs	4107.4 (3145.2 to 5173.6)	3075.9 (2242.6 to 4023.2)	4993.4 (3681 to 6570.6)	1994.6 (1572.9 to 2484)	1617.1 (1208.1 to 2092.7)	2393.9 (1818.4 to 3042.8)	-51.4 (-60.9 to -38.2)	-47.4 (-60.7 to -29.1)	-52.1 (-64.6 to -33.6)
	YLLs	4012.2 (3072.7 to 5073.9)	2972.7 (2149.7 to 3903.9)	4905.4 (3609.9 to 6466.8)	1904.2 (1502.3 to 2372.5)	1525.6 (1133.7 to 1967)	2304.5 (1744.9 to 2948.5)	-52.5 (-61.9 to -39)	-48.7 (-62.1 to -29.8)	-53 (-65.6 to -34.3)
	YLDs	95.2 (62.8 to 136.7)	103.2 (67.1 to 152.1)	88 (58.1 to 125)	90.4 (59.9 to 129.3)	91.4 (59.7 to 134.6)	89.5 (59.1 to 124.4)	-5 (-10.5 to 0.7)	-11.4 (-18.5 to -4.4)	1.7 (-5.6 to 10.7)
Khorasan-e-Razavi	Deaths	166 (121.9 to 222.7)	148 (102.1 to 204)	181.4 (129.2 to 250.4)	79.7 (56.8 to 107.7)	73.3 (49.5 to 104.1)	86.3 (60.9 to 116.9)	-52 (-61.1 to -42.8)	-50.5 (-61.8 to -36.2)	-52.4 (-64.2 to -37.3)
	DALYs	3555.5 (2714.7 to 4601)	2935.1 (2112.7 to 3904.1)	4116.9 (3048.2 to 5520.5)	1577 (1229.5 to 2012.1)	1278.3 (942 to 1673)	1878.2 (1445 to 2403.7)	-55.6 (-64.9 to -44.9)	-56.4 (-67.5 to -42.1)	-54.4 (-66.2 to -37.4)
	YLLs	3464.1 (2629.1 to 4472.3)	2837.8 (2034.1 to 3766.5)	4031 (2952.7 to 5433.6)	1497.4 (1149.6 to 1902.3)	1199.2 (881.9 to 1577.1)	1798 (1374 to 2312.3)	-56.8 (-65.9 to -45.8)	-57.7 (-69 to -43.2)	-55.4 (-67.3 to -38.2)
	YLDs	91.4 (59.8 to 131.7)	97.3 (63.8 to 146)	85.9 (56.7 to 121.4)	79.6 (52.4 to 113.6)	79.1 (50.9 to 115)	80.2 (52.8 to 113.7)	-13 (-17.7 to -7.7)	-18.7 (-25.2 to -11.4)	-6.7 (-13.3 to 0.7)

Province	Measure	Attributed age-standardized rate (per 100,000)						% Change (1990 to 2019)		
		1990			2019					
		Both	Female	Male	Both	Female	Male	Both	Female	Male
Khuzestan	Deaths	168 (123.3 to 223.6)	147.7 (104.7 to 200.6)	186.2 (131.8 to 257.1)	112.2 (82.6 to 149.6)	105 (71.7 to 143.2)	119.7 (87.8 to 159.6)	-33.2 (-44.6 to -19.3)	-28.9 (-44.2 to -8.7)	-35.7 (-51.4 to -16.1)
	DALYs	3713.3 (2872.2 to 4727.3)	3050.8 (2278.7 to 3978.9)	4309.3 (3131.5 to 5844.9)	2241.5 (1791.4 to 2823)	1867.7 (1399.5 to 2423.1)	2609.3 (2039.6 to 3340.2)	-39.6 (-51.1 to -25.2)	-38.8 (-52.8 to -19.6)	-39.4 (-55.1 to -18.3)
	YLLs	3612.8 (2775.2 to 4604.8)	2943.7 (2196.2 to 3875.7)	4214.9 (3040.3 to 5742.5)	2140.3 (1692.8 to 2704.6)	1765.9 (1308.5 to 2288.7)	2508.5 (1950.5 to 3222.2)	-40.8 (-52.4 to -26)	-40 (-54.2 to -20.4)	-40.5 (-56.3 to -18.9)
	YLDs	100.5 (66.6 to 143.4)	107.1 (70.2 to 154)	94.4 (62.6 to 132.6)	101.2 (67 to 146.4)	101.7 (65.8 to 150.4)	100.8 (66.4 to 143.3)	0.7 (-5.3 to 7.1)	-5 (-13.1 to 3.2)	6.8 (-1.2 to 15.9)
Kohgiluyeh and Boyer-Ahmad	Deaths	150 (107.3 to 199.5)	141.2 (95.7 to 195.1)	154.5 (105.6 to 212.6)	99.5 (70.9 to 134.2)	104.9 (69.5 to 145.7)	97.8 (68.7 to 135.9)	-33.6 (-47.2 to -16.3)	-25.7 (-44.6 to 0.2)	-36.7 (-53.9 to -12.9)
	DALYs	3162.7 (2408.8 to 4026.6)	2720.1 (1996.2 to 3590.2)	3498.4 (2482.1 to 4712.4)	1922.3 (1501.9 to 2440.2)	1778.5 (1282.4 to 2310)	2094.9 (1554.8 to 2793.8)	-39.2 (-52.8 to -21.6)	-34.6 (-52.3 to -9.1)	-40.1 (-57.4 to -14.9)
	YLLs	3072.2 (2326.4 to 3940.5)	2625.6 (1904.2 to 3495.7)	3411.2 (2413.2 to 4609.6)	1832.8 (1412.4 to 2333.5)	1688.2 (1206.8 to 2232.6)	2006.2 (1474.7 to 2690.6)	-40.3 (-54.2 to -22.2)	-35.7 (-53.5 to -9)	-41.2 (-58.6 to -15.5)
	YLDs	90.6 (59.7 to 128.9)	94.5 (62 to 136.7)	87.2 (57.8 to 123.4)	89.5 (59.4 to 127.3)	90.4 (59.9 to 133.1)	88.7 (58.5 to 125.3)	-1.2 (-6.5 to 4.4)	-4.3 (-12.2 to 4.2)	1.6 (-6.3 to 9.5)
Kurdistan	Deaths	156 (114.7 to 204.7)	130.5 (93.1 to 178.9)	177.9 (126.3 to 237.8)	81.4 (58.4 to 107.6)	76.4 (52.1 to 105.7)	87.7 (62.4 to 117.3)	-47.8 (-57.7 to -36.9)	-41.4 (-54.5 to -26.5)	-50.7 (-62.7 to -35.7)
	DALYs	3456.5 (2710.4 to 4415)	2721.1 (2043.6 to 3565.2)	4091.5 (3027.1 to 5430.3)	1624.2 (1284.3 to 2025.8)	1315.5 (990 to 1673.2)	1946 (1491 to 2494.8)	-53 (-62.2 to -42.1)	-51.7 (-63.3 to -38.2)	-52.4 (-64.5 to -36.5)
	YLLs	3373.4 (2634.5 to 4316.4)	2632.9 (1971 to 3476.5)	4013.2 (2965.6 to 5337.1)	1543.2 (1217.1 to 1929.3)	1235.4 (915.1 to 1578.7)	1864.1 (1413.1 to 2398.5)	-54.3 (-63.5 to -43)	-53.1 (-64.5 to -39.3)	-53.6 (-65.7 to -37.4)
	YLDs	83.1 (54.4 to 118.7)	88.2 (57.4 to 129.1)	78.3 (51.7 to 111.4)	81 (53.8 to 115.7)	80.1 (51.9 to 118.5)	82 (55.1 to 114.3)	-2.6 (-8.2 to 3.6)	-9.2 (-16.5 to -0.6)	4.6 (-3.8 to 13.7)

Province	Measure	Attributed age-standardized rate (per 100,000)						% Change (1990 to 2019)		
		1990			2019					
		Both	Female	Male	Both	Female	Male	Both	Female	Male
Lorestan	Deaths	161 (118.9 to 212)	133.3 (92.2 to 181.8)	184.7 (131.3 to 248.8)	104.3 (75.2 to 136.7)	80.7 (55.4 to 109)	128.7 (93.1 to 171.3)	-35.2 (-47.6 to -20.9)	-39.5 (-54 to -22.4)	-30.3 (-47.6 to -8.6)
	DALYs	3513.1 (2712 to 4436.8)	2702.2 (1994.9 to 3543)	4200.2 (3075.1 to 5635.1)	2059.2 (1601.4 to 2585.9)	1438 (1075.4 to 1855.2)	2702.3 (2079.1 to 3461.5)	-41.4 (-53.7 to -26.6)	-46.8 (-61 to -30.3)	-35.7 (-52.4 to -11.9)
	YLLs	3421.8 (2633.4 to 4326.2)	2605.3 (1912.9 to 3438.7)	4114 (3001.3 to 5541.8)	1974 (1531.5 to 2483.9)	1352.1 (1003.2 to 1754.3)	2617.7 (2006.9 to 3370.8)	-42.3 (-54.6 to -27.1)	-48.1 (-62.3 to -31.2)	-36.4 (-53.3 to -12.2)
	YLDs	91.3 (59.9 to 131.5)	96.9 (62.7 to 142.7)	86.2 (56.7 to 122)	85.2 (56.3 to 122.6)	85.9 (56.3 to 125)	84.6 (55.6 to 120)	-6.7 (-12.4 to -1.2)	-11.3 (-18.5 to -3.5)	-1.8 (-10 to 6.2)
Markazi	Deaths	145.9 (104.6 to 192.9)	126.3 (87.1 to 176.2)	164.1 (116.4 to 224.4)	70.6 (50.1 to 95.9)	63.9 (42.3 to 88.6)	77.8 (54.8 to 105.7)	-51.6 (-60.9 to -41)	-49.4 (-61.2 to -33.7)	-52.6 (-64.3 to -36.3)
	DALYs	3156.6 (2382.9 to 4079.9)	2478 (1781.8 to 3285.9)	3817.6 (2748.4 to 5144.2)	1442.6 (1109.4 to 1827.1)	1102.4 (819.2 to 1439.9)	1779.3 (1343.2 to 2299.4)	-54.3 (-63.1 to -42.9)	-55.5 (-66.2 to -41.2)	-53.4 (-65.6 to -36.7)
	YLLs	3060.6 (2306.8 to 3945.2)	2375.7 (1700.7 to 3172.2)	3727.9 (2673.4 to 5050)	1355.3 (1040.2 to 1723.1)	1015.9 (747.6 to 1322.8)	1691.3 (1268.6 to 2212.6)	-55.7 (-64.6 to -44.1)	-57.2 (-68.1 to -42.4)	-54.6 (-66.8 to -37.8)
	YLDs	96 (62.5 to 137.6)	102.2 (66.6 to 149.7)	89.7 (59.2 to 129.1)	87.2 (58 to 123)	86.5 (56.4 to 126.5)	88 (58.9 to 124.3)	-9.2 (-15.2 to -3.1)	-15.4 (-22.9 to -7.7)	-1.9 (-10 to 7.5)
Mazandaran	Deaths	148.2 (106.7 to 197.8)	140.9 (97.8 to 193.8)	152.6 (108.2 to 211.8)	78.7 (56.3 to 104.8)	73.1 (50.4 to 101.7)	84.4 (60.2 to 113.7)	-46.9 (-56.6 to -35.1)	-48.2 (-59.9 to -31.9)	-44.7 (-58.7 to -25.8)
	DALYs	3061.3 (2352.3 to 3860)	2707.9 (2002.1 to 3588.9)	3378.9 (2481.6 to 4494.9)	1602.1 (1258 to 2007.9)	1310.5 (991.3 to 1706.1)	1894.1 (1450.3 to 2413.8)	-47.7 (-57.3 to -34.9)	-51.6 (-63.1 to -35.4)	-43.9 (-58.8 to -22.7)
	YLLs	2958 (2275.5 to 3736.7)	2596.3 (1910.9 to 3474.6)	3283.8 (2394.1 to 4423.9)	1511.5 (1186.7 to 1902.4)	1219.9 (921.5 to 1606.6)	1803.4 (1371.5 to 2300.6)	-48.9 (-58.7 to -35.8)	-53 (-64.8 to -36.7)	-45.1 (-60.1 to -23.3)
	YLDs	103.3 (69 to 148.2)	111.6 (74.4 to 162.1)	95.1 (63.3 to 135.2)	90.6 (60.2 to 129.5)	90.7 (59.3 to 132.5)	90.7 (60.5 to 129.4)	-12.2 (-17.9 to -6.5)	-18.8 (-25.3 to -11.2)	-4.5 (-11.7 to 3.6)

Province	Measure	Attributed age-standardized rate (per 100,000)						% Change (1990 to 2019)		
		1990			2019					
		Both	Female	Male	Both	Female	Male	Both	Female	Male
North Khorasan	Deaths	145.5 (104 to 193.5)	132.1 (92.7 to 180.9)	156.2 (111.9 to 217.3)	87.5 (62 to 115.2)	91.4 (63.4 to 123.4)	86 (62.2 to 114.6)	-39.8 (-50.7 to -27.4)	-30.8 (-46 to -11)	-45 (-57.8 to -28.4)
	DALYs	3275.1 (2529.8 to 4164.7)	2785.8 (2036.9 to 3665.2)	3710.5 (2785.8 to 5046.3)	1804.9 (1409.3 to 2299.5)	1697 (1294.1 to 2172.2)	1935.7 (1504.8 to 2502)	-44.9 (-55 to -33)	-39.1 (-53.2 to -20.9)	-47.8 (-60.3 to -30.7)
	YLLs	3181.5 (2463.1 to 4079)	2682.3 (1955.2 to 3544.8)	3626.4 (2717.6 to 4954.3)	1716.9 (1331.8 to 2176.7)	1604.4 (1205.1 to 2070.7)	1852.4 (1446.8 to 2413.6)	-46 (-56.2 to -33.8)	-40.2 (-54.4 to -21.6)	-48.9 (-61.6 to -31.5)
	YLDs	93.6 (61.3 to 134.6)	103.4 (67.5 to 152.3)	84.1 (54.7 to 121)	88 (58.2 to 126.3)	92.7 (61 to 139)	83.3 (55.8 to 116.7)	-5.9 (-11.2 to -0.1)	-10.4 (-17.6 to -2.6)	-0.9 (-9 to 7.6)
Qazvin	Deaths	124.5 (88.3 to 165.6)	105.1 (70.4 to 144.8)	144.8 (100.5 to 197.4)	81.1 (57.2 to 107.8)	62.4 (41.6 to 86.5)	100.6 (71.6 to 133.3)	-34.9 (-47.9 to -20.2)	-40.6 (-54.8 to -22.2)	-30.5 (-47.8 to -8.8)
	DALYs	2652 (1999.5 to 3400.9)	2104.4 (1529.4 to 2764.7)	3178.7 (2302.8 to 4303.7)	1562.9 (1211.9 to 1960.7)	1070.6 (791.2 to 1411)	2060.2 (1591 to 2613.7)	-41.1 (-53.3 to -25.5)	-49.1 (-61.5 to -32.5)	-35.2 (-52.2 to -12.8)
	YLLs	2568.7 (1936.7 to 3309.5)	2017.5 (1445.1 to 2664.4)	3098.8 (2228.4 to 4240.6)	1482.8 (1144.2 to 1867.3)	992.3 (724.8 to 1305.5)	1978 (1523 to 2524.7)	-42.3 (-54.6 to -26.2)	-50.8 (-63.3 to -33.6)	-36.2 (-53.4 to -13.3)
	YLDs	83.4 (54.8 to 118.7)	87 (57.4 to 126.2)	80 (52.3 to 112.5)	80.2 (52.8 to 113.9)	78.4 (51 to 116.1)	82.1 (53.9 to 115.9)	-3.8 (-9.7 to 2)	-9.9 (-17.4 to -2.5)	2.7 (-4.9 to 10.5)
Qom	Deaths	129 (88.7 to 176.2)	119.7 (76.6 to 165.5)	135.7 (89.9 to 187.5)	77 (53.8 to 103.3)	85.9 (56.6 to 121.6)	74 (52.2 to 99.9)	-40.3 (-52.9 to -22.9)	-28.3 (-44.6 to -2.5)	-45.5 (-60.4 to -22.5)
	DALYs	2683.9 (1918.5 to 3526.7)	2240.4 (1498.2 to 2999.7)	3067.2 (2055.4 to 4158.2)	1392.2 (1077.3 to 1760)	1307.3 (952.2 to 1716.2)	1510.1 (1127.3 to 1950.9)	-48.1 (-59.2 to -29.9)	-41.6 (-55.5 to -17.5)	-50.8 (-64.3 to -28.1)
	YLLs	2587 (1825.8 to 3390.7)	2139 (1417.1 to 2872.2)	2974.2 (1961.2 to 4051)	1312.2 (998.4 to 1659.3)	1228.1 (888.2 to 1619.8)	1429.2 (1067.9 to 1831.6)	-49.3 (-60.3 to -30.4)	-42.6 (-56.9 to -17.1)	-51.9 (-65.5 to -28.9)
	YLDs	96.9 (64.2 to 137.6)	101.4 (66.5 to 147.4)	93 (61.8 to 130.8)	80 (53.1 to 113.4)	79.2 (51.7 to 115.8)	80.8 (54.3 to 115.1)	-17.4 (-23 to -11.9)	-21.9 (-29 to -14.6)	-13.1 (-20.4 to -5.4)

Province	Measure	Attributed age-standardized rate (per 100,000)						% Change (1990 to 2019)		
		1990			2019					
		Both	Female	Male	Both	Female	Male	Both	Female	Male
Semnan	Deaths	137.6 (99.2 to 181.8)	111.7 (74.8 to 153.3)	165.7 (118.4 to 221.7)	82.1 (57.9 to 108.3)	63.5 (42.8 to 87.4)	101.2 (72.2 to 130.8)	-40.3 (-51.1 to -26.9)	-43.1 (-56.5 to -23.8)	-38.9 (-53.3 to -19.5)
	DALYs	3021.3 (2249.2 to 3831.8)	2265.9 (1587.4 to 2997.3)	3784.1 (2784.2 to 4981.3)	1620.6 (1258.9 to 2001.8)	1122 (833.8 to 1454.8)	2120.9 (1630.1 to 2639.2)	-46.4 (-56.7 to -32.5)	-50.5 (-62.6 to -33.5)	-44 (-57.7 to -23.2)
	YLLs	2919.7 (2170.3 to 3719.9)	2159.3 (1479.3 to 2879.9)	3687.4 (2703 to 4887.9)	1530.5 (1184.4 to 1898.6)	1032.6 (755 to 1346.7)	2029.8 (1549.2 to 2531.5)	-47.6 (-58 to -33.4)	-52.2 (-64.2 to -34.9)	-45 (-58.6 to -23.6)
	YLDs	101.6 (67.8 to 146.1)	106.7 (71 to 155.7)	96.7 (64.7 to 138.7)	90.2 (59.3 to 129.3)	89.4 (58.6 to 131.3)	91 (60.2 to 129.6)	-11.3 (-16.6 to -5.8)	-16.2 (-23.5 to -8.6)	-5.9 (-12.7 to 1.6)
Sistan and Baluchistan	Deaths	131.7 (91.2 to 175.4)	112 (75.8 to 156.1)	148.7 (97.1 to 204.3)	77.6 (56.6 to 101.3)	71.5 (50.6 to 96.9)	84 (58.9 to 112.7)	-41.1 (-53.3 to -21.8)	-36.2 (-53.2 to -12.4)	-43.5 (-58.3 to -18.2)
	DALYs	3013.8 (1987.5 to 3914)	2474.6 (1715.4 to 3363.8)	3455 (2167.7 to 4696.1)	1816.8 (1424 to 2252.6)	1588.2 (1207 to 2044.1)	2050.4 (1520.4 to 2682.8)	-39.7 (-53.5 to -12)	-35.8 (-53.8 to -6.6)	-40.7 (-57.6 to -5.7)
	YLLs	2932.6 (1930.5 to 3820.1)	2387 (1625.4 to 3269)	3379.4 (2109.2 to 4628.7)	1743.8 (1352.1 to 2169.1)	1513.4 (1137.8 to 1939)	1979.3 (1463.6 to 2598.5)	-40.5 (-54.6 to -12.2)	-36.6 (-55 to -5.8)	-41.4 (-58.3 to -5.8)
	YLDs	81.2 (53.6 to 114.4)	87.6 (56.9 to 126)	75.6 (49.9 to 106.5)	73 (48.1 to 104)	74.8 (49.2 to 108.3)	71.1 (46.5 to 100.5)	-10.1 (-15.5 to -4.3)	-14.6 (-21.1 to -7.1)	-6 (-13.7 to 1.9)
South Khorasan	Deaths	103.5 (74.6 to 138)	88.1 (59.8 to 122.2)	117.7 (82.6 to 160.7)	65.2 (45.9 to 88.2)	57.6 (39 to 80.3)	74.2 (52.1 to 101.5)	-37 (-48.8 to -23.6)	-34.6 (-49.3 to -17)	-36.9 (-51.1 to -17.7)
	DALYs	2215.5 (1698 to 2885)	1770.2 (1295.3 to 2301.4)	2619.7 (1917.8 to 3498.8)	1235.6 (961.6 to 1564.2)	998.7 (746.6 to 1323.6)	1492.7 (1152.9 to 1930.1)	-44.2 (-54.8 to -32.5)	-43.6 (-56.5 to -27.6)	-43 (-56.8 to -24.3)
	YLLs	2136.3 (1630.4 to 2769.3)	1686 (1215.8 to 2207)	2545 (1846.8 to 3403)	1160.3 (899.8 to 1478.4)	924.5 (685.8 to 1221)	1416.2 (1084.9 to 1856.2)	-45.7 (-56.3 to -33.4)	-45.2 (-58.3 to -28.6)	-44.4 (-58.1 to -25.1)
	YLDs	79.3 (51.7 to 114.2)	84.1 (53.8 to 124.4)	74.6 (49 to 106.5)	75.3 (49.4 to 108.5)	74.3 (48.1 to 110.6)	76.5 (50 to 109.5)	-5 (-10.5 to 0.9)	-11.7 (-19 to -3.5)	2.5 (-5.2 to 10.5)

Province	Measure	Attributed age-standardized rate (per 100,000)						% Change (1990 to 2019)		
		1990			2019					
		Both	Female	Male	Both	Female	Male	Both	Female	Male
Tehran	Deaths	81 (55.8 to 111)	77.7 (50.4 to 112.7)	82.1 (55.9 to 114.4)	42.8 (29.9 to 59)	45.3 (29.4 to 65.6)	41.5 (30.1 to 57.1)	-47.1 (-58.9 to -32.7)	-41.6 (-56.9 to -19.4)	-49.4 (-63.8 to -29.2)
	DALYs	1644.7 (1231.9 to 2144.4)	1370.1 (964 to 1885.8)	1868.7 (1348.3 to 2539.5)	825.1 (640.8 to 1058.2)	722.4 (520.7 to 995.9)	935.9 (721.3 to 1234.2)	-49.8 (-61.2 to -35.6)	-47.3 (-61.4 to -28.2)	-49.9 (-64.2 to -30.3)
	YLLs	1549.3 (1159.6 to 2033.4)	1274 (877.4 to 1787)	1773.8 (1248.1 to 2441.6)	744.4 (575.5 to 963.1)	645 (457.1 to 897.6)	852 (647.9 to 1133.8)	-52 (-63.9 to -37.2)	-49.4 (-64 to -29.5)	-52 (-66.6 to -31.2)
	YLDs	95.4 (63.4 to 135.1)	96.1 (63.5 to 137.2)	94.9 (63 to 134.5)	80.7 (53.5 to 114.3)	77.5 (51.2 to 112.3)	83.9 (55.3 to 117.4)	-15.4 (-19.9 to -10.8)	-19.4 (-25.9 to -12.9)	-11.6 (-17.6 to -4.8)
West Azarbayejan	Deaths	180.4 (131.8 to 237)	157.8 (108.2 to 215.5)	201.1 (144.2 to 271.3)	105.7 (72.7 to 146.4)	100.5 (67.6 to 141.4)	110.9 (75.9 to 151.7)	-41.4 (-52 to -30)	-36.3 (-50.3 to -19.6)	-44.8 (-57.9 to -28.4)
	DALYs	3710.6 (2946.1 to 4622.8)	3010.4 (2240.2 to 3889.9)	4353.1 (3281.7 to 5723.9)	1879.6 (1435.1 to 2379.2)	1573.2 (1175.4 to 2096.5)	2184.1 (1619 to 2802.8)	-49.3 (-59.4 to -37.7)	-47.7 (-59.7 to -32.1)	-49.8 (-62.6 to -32.8)
	YLLs	3620.1 (2865.5 to 4531.5)	2911.7 (2160.4 to 3769.1)	4270.4 (3201.9 to 5618.6)	1794.7 (1363.8 to 2273.1)	1486.7 (1106.3 to 1982)	2100.6 (1555.6 to 2701.6)	-50.4 (-60.6 to -38.5)	-48.9 (-61.1 to -33.1)	-50.8 (-63.6 to -33.5)
	YLDs	90.5 (59.4 to 130.3)	98.6 (64.9 to 147.6)	82.7 (54.3 to 117.1)	84.9 (56.1 to 122.5)	86.6 (56.3 to 126.1)	83.4 (55.4 to 121.1)	-6.1 (-11.6 to -0.6)	-12.2 (-19.5 to -5)	0.8 (-7.6 to 9.1)
Yazd	Deaths	138.5 (99.3 to 185.8)	119.6 (78.3 to 168.6)	156.7 (111.5 to 215.2)	79.5 (56.6 to 108.3)	69.8 (45.6 to 98)	88.9 (62.2 to 121)	-42.6 (-54.1 to -29.4)	-41.6 (-55.2 to -22.9)	-43.3 (-58 to -23)
	DALYs	2864.7 (2173.8 to 3691.6)	2259 (1605 to 3031.3)	3446.6 (2499.3 to 4635.7)	1472.1 (1150.2 to 1898.4)	1132.1 (815 to 1509.9)	1785.6 (1357 to 2343.7)	-48.6 (-59.1 to -34.5)	-49.9 (-62 to -31.9)	-48.2 (-62.3 to -27.3)
	YLLs	2770 (2087 to 3590.1)	2159.7 (1508.9 to 2911.3)	3356.1 (2425.8 to 4541)	1381.2 (1074.1 to 1794.9)	1042.1 (734.9 to 1413.2)	1693.9 (1271.9 to 2252.7)	-50.1 (-60.7 to -35.6)	-51.7 (-64 to -33.4)	-49.5 (-63.7 to -28.4)
	YLDs	94.7 (62.7 to 135.4)	99.4 (64.6 to 143.9)	90.5 (59.7 to 128.6)	90.9 (59.9 to 129.9)	90 (58.8 to 132.9)	91.8 (61.7 to 127.6)	-4 (-9.6 to 1.7)	-9.5 (-16.8 to -1.5)	1.5 (-5.6 to 9.9)

Province	Measure	Attributed age-standardized rate (per 100,000)						% Change (1990 to 2019)		
		1990			2019					
		Both	Female	Male	Both	Female	Male	Both	Female	Male
Zanjan	Deaths	132.6 (94.4 to 178.3)	110.7 (76.4 to 152.5)	155.9 (109.2 to 215.4)	82.5 (58.4 to 110.5)	68.5 (47 to 94.3)	97.5 (67.4 to 130.5)	-37.8 (-47.5 to -26.5)	-38.1 (-51.1 to -23.5)	-37.4 (-50.8 to -20.8)
	DALYs	2780.3 (2102.7 to 3593.4)	2189.3 (1629.1 to 2866.9)	3345.2 (2459.6 to 4500.7)	1521.7 (1173.3 to 1915.1)	1160.8 (862.7 to 1506.2)	1897.8 (1448.1 to 2390.6)	-45.3 (-55 to -34.3)	-47 (-59.3 to -33.4)	-43.3 (-56.5 to -26.2)
	YLLs	2688.7 (2039.5 to 3482.5)	2091.8 (1548.9 to 2767.5)	3259.4 (2391.4 to 4422.8)	1437.4 (1101.6 to 1802.7)	1075.2 (794.9 to 1411)	1814.8 (1380.9 to 2289.5)	-46.5 (-56.2 to -35.3)	-48.6 (-61.1 to -34.4)	-44.3 (-57.5 to -26.9)
	YLDs	91.7 (61.1 to 132.5)	97.5 (63.8 to 141.5)	85.9 (56.4 to 123.8)	84.3 (55.6 to 121)	85.6 (55.6 to 125.4)	83 (54.8 to 117.3)	-8.1 (-13.5 to -2.2)	-12.3 (-19.9 to -4.3)	-3.3 (-10.8 to 5.1)

*Data in parentheses are 95% Uncertainty Intervals (95% UIs)

Authors' Contributions

Providing data or critical feedback on data sources

Ali M Ahmadi, Sepideh Ahmadi, Davood Anvari, Jalal Arabloo, Armin Aryannejad, Elaheh Askari, Seyyed Shamsadin Athari, Sina Azadnajafabad, Samad Azari, Sara Bagherieh, Azadeh Bashiri, Mostafa Dianatinasab, Milad Dodangeh, Hossein Farrokhpour, Farshad Farzadfar, Ali Fatehizadeh, Ahmad Ghashghaee, Pouya Goleij, Nima Hafezi-Nejad, Soheil Hassanipour, Rana Irilouzadian, Tannaz Jamialahmadi, Morteza A Khafaie, Shaghayegh Khanmohammadi, Sorour Khateri, Ata Mahmoodpoor, Mohammad Ali Mansournia, Abdoljalal Marjani, Sara Momtazmanesh, Soroush Moradi, Maryam Moradi, Maziar Moradi-Lakeh, Mohsen Naghavi, Hasti Nouraei, Mehran Rahimi, Shayan Rahmani, Sina Rashedi, Sahar Saeedi Moghaddam, Mehran Shams-Beyranvand, Parnian Shobeiri, Majid Taheri, Iman Zare, and Zahra Zareshahrabadi.

Developing methods or computational machinery

Sina Azadnajafabad, Soroush Moradi, Mohsen Naghavi, and Sahar Saeedi Moghaddam.

Providing critical feedback on methods or results

Amirali Aali, Shima Abdollahi, Abdorrahim Absalan, Siamak Afaghi, Ali M Ahmadi, Amir Ahmadi, Meisam Akhlaghdoust, Sudabeh Alatab, Yousef Alimohamadi, Sohrab Amiri, Davood Anvari, Jalal Arabloo, Armin Aryannejad, Elaheh Askari, Seyyed Shamsadin Athari, Abolfazl Avan, Sina Azadnajafabad, Samad Azari, Hassan Babamohamadi, Nayereh Baghcheghi, Sara Bagherieh, Hamid Reza Baradaran, Azadeh Bashiri, Mostafa Dianatinasab, Shirin Djalalinia, Milad Dodangeh, Sareh Edalati, Zahra Esfahani, Hossein Farrokhpour, Farshad Farzadfar, Ali Fatehizadeh, Nima Hafezi-Nejad, Soheil Hassanipour, Mahsa Heidari-Foroozan, Kamran Hessami, Kaveh Hosseini, Mohammad-Salar Hosseini, Seyed Kianoosh Hosseini, Soodabeh Hoveidamanesh, Farideh Iravanpour, Rana Irilouzadian, Zahra Jamalpoor, Tannaz Jamialahmadi, Ali Kabir, Sina Kazemian, Mohammad Keykhaei, Morteza A Khafaie, Shaghayegh Khanmohammadi, Farzad Kompani, Hamid Reza Koohestani, Bagher Larijani, Ata Mahmoodpoor, Marzieh Mahmoudimanesh, Elaheh Malakan Rad, Mohammad-Reza Malekpour, Reza Malekzadeh, Mohammad Ali Mansournia, Abdoljalal Marjani, Sara Momtazmanesh, Soroush Moradi, Maryam Moradi, Maziar Moradi-Lakeh, Farhad Moradpour, Negar Morovatdar, Mohsen Naghavi, Seyed Aria Nejadghaderi, Maryam Noori, Ali Nowroozi, Hassan Okati-Aliabad, Amirhossein Parsaei, Akram Pourshams, Mehran Rahimi, Shayan Rahmani, Vahid Rahmanian, Sina Rashedi, Mohammad-Mahdi Rashidi, Iman Razeghian-Jahromi, Malihe Rezaee, Nazila Rezaei, Negar Rezaei, Leila Sabzmakan, Erfan Sadeghi, Saeid Sadeghian, Sahar Saeedi Moghaddam, Hamideh Salimzadeh, Saman Sargazi, Nizal Sarrafzadegan, Sadaf G Sepanlou, Mahan Shafie, Ataollah Shahbandi, Mehran Shams-Beyranvand, Athena Sharifi-Razavi, Parnian Shobeiri, Seyed Afshin Shorofi, Seyed-Amir Tabatabaeizadeh, Alireza Tahamtan, Majid Taheri, Seyed Mohammad Vahabi, Siavash Vaziri, Fereshteh Yazdanpanah, Mazyar Zahir, and Moein Zangiabadian.

Drafting the work or revising is critically for important intellectual content

Amirali Aali, Mohsen Abbasi-Kangevari, Zeinab Abbasi-Kangevari, Shima Abdollahi, Siamak Afaghi, Ali M Ahmadi, Sepideh Ahmadi, Marjan Ajami, Meisam Akhlaghdoust, Mehrdad Amir-Behghadami, Sohrab Amiri, Jalal Arabloo, Armin Aryannejad, Elaheh Askari, Abolfazl Avan, Sina Azadnajafabad, Hassan

Babamohamadi, Sara Bagherieh, Azadeh Bashiri, Mostafa Dianatinasab, Milad Dodangeh, Mahsa Dolatshahi, Zahra Esfahani, Farshad Farzadfar, Ali Fatehizadeh, Fataneh Ghadirian, Ali Gholami, Nima Hafezi-Nejad, Hamidreza Hasani, Kamran Hessami, Kaveh Hosseini, Mohammad-Salar Hosseini, Farideh Iravanpour, Rana Irilouzadian, Tannaz Jamialahmadi, Ali Kabir, Sina Kazemian, Shaghayegh Khanmohammadi, Farzad Kompani, Bagher Larijani, Soleiman Mahjoub, Ata Mahmoodpoor, Elaheh Malakan Rad, Mohammad-Reza Malekpour, Reza Malekzadeh, abdoljalal marjani, Baharnaz Mashinchi, Esmaeil Mohammadi, Sara Momtazmanesh, Soroush Moradi, Maryam Moradi, Maziar Moradi-Lakeh, Negar Morovatdar, Mohsen Naghavi, Seyed Aria Nejadghaderi, Ali Nowroozi, Amirhossein Parsaei, Mehran Rahimi, Shayan Rahmani, Sina Rashedi, Iman Razeghian-Jahromi, Malihe Rezaee, Nazila Rezaei, Negar Rezaei, Leila Sabzmakan, Masoumeh Sadeghi, Sahar Saeedi Moghaddam, Amirhossein Sahebkar, Hamideh Salimzadeh, Sadaf G Sepanlou, Melika Shafeghat, Mahan Shafie, Fariba Shahraki-Sanavi, Mehran Shams-Beyranvand, Parnian Shobeiri, Seyed Afshin Shorofi, Seyed-Amir Tabatabaeizadeh, Majid Taheri, Mazyar Zahir, and Iman Zare.

Managing the estimation or publications process

Sina Azadnajafabad, Farshad Farzadfar, Bagher Larijani, Soroush Moradi, Mohsen Naghavi, Negar Rezaei, and Sahar Saeedi Moghaddam.
